# Supplementary material for: Mediation and moderation analysis of the association between physical capability and quality of life among stroke patients
Source: PLoS One. 2025 Jun 10;20(6):e0320833. doi: 10.1371/journal.pone.0320833 (PMC12151468; doi:10.1371/journal.pone.0320833)
Supplement: Attachment — Submitted filename: PONE-D-24-45706_reviewer.pdf [file pone.0320833.s003.pdf]

PLOS ONE

Mediation and moderation analysis of the association between physical capability and quality of life among stroke patients

--Manuscript Draft--

**Manuscript Number:**	PONE-D-24-45706
**Article Type:**	Research Article
**Full Title:**	Mediation and moderation analysis of the association between physical capability and quality of life among stroke patients
**Short Title:**	SEM of the association between physical capability and quality of life among stroke patients
**Corresponding Author:**	Liqing Yao<br>The Secomd Affiliated Hospital of Kunming Medical University<br>Kunming, CHINA
**Keywords:**	stroke, mediation, moderation, self-efficacy, quality of life
**Abstract:**	Purpose: To estimate the extent to which physical capacity, depressive symptoms, balanced self-efficacy (BSE), and other risk factors interrelated with stroke to influence quality of life (QoL) in stroke patients. A theoretical model based on Wilson and Cleary model tested the specific hypotheses: (1) physical capability has a direct and indirect effect on QoL mediated by BSE and depressive symptoms; (2) stroke risk factors (hypertension/diabetes/gender) moderate the above relationship.<br>Method: Six hundred and seventy stroke patients were enrolled from ten different hospitals at Yunnan province from 2019 to 2021. Patients' mental and physical function was assessed using Brunnstrom recovery stage (BRS), mini-balance evaluation system test (Mini-BEST), Barthel index (BI), Activities-specific Balance Confidence scale (ABC), and Hamilton depression scale (HAM-D). Structural equation model (SEM) was used to test the moderated mediation model in Mplus 8.0 software.<br>Results: The model showed good fit (RMSEA = 0.075, SRMR = 0.010). BSE significantly mediated the relationship between physical capability and QoL (β = 0.322, P = 0.002). Hypertension was found a significant moderator of all the direct paths from 'physical capability to QoL through depressive symptoms (B = 0.412, P = 0.015; B = 0.831, P = 0.020, respectively).<br>Conclusions: This study provides a better insight into the relationship between physical capability and QoL via BSE in stroke patients, which may help establish appropriate treatment for these individuals.
**Order of Authors:**	Liqing Yao
	Xue Yang
	hongmei Zhang
	qian Liu
	Yihuan Lu
**Additional Information:**	
**Question**	**Response**
**Financial Disclosure**<br><br>Enter a financial disclosure statement that describes the sources of funding for the work included in this submission. Review the submission guidelines for detailed requirements. View published research articles from *PLOS ONE* for specific examples.	Yes

Powered by Editorial Manager® and ProduXion Manager® from Aries Systems Corporation

This statement is required for submission and **will appear in the published article** if the submission is accepted. Please make sure it is accurate.<br><br>**Funded studies**<br>Enter a statement with the following details:<br>• Initials of the authors who received each award<br>• Grant numbers awarded to each author<br>• The full name of each funder<br>• URL of each funder website<br>• Did the sponsors or funders play any role in the study design, data collection and analysis, decision to publish, or preparation of the manuscript?<br><br>Did you receive funding for this work?	
Please add funding details.<br>as follow-up to "**Financial Disclosure**<br><br>Enter a financial disclosure statement that describes the sources of funding for the work included in this submission. Review the submission guidelines for detailed requirements. View published research articles from *PLOS ONE* for specific examples.<br><br>This statement is required for submission and **will appear in the published article** if the submission is accepted. Please make sure it is accurate.<br><br>**Funded studies**<br>Enter a statement with the following details:<br>• Initials of the authors who received each award<br>• Grant numbers awarded to each author<br>• The full name of each funder<br>• URL of each funder website<br>• Did the sponsors or funders play any role in the study design, data collection and analysis, decision to publish, or preparation of the manuscript?	The work was supported by the Major Science and Technology Project of Yunnan Province (Grant numbers[2018zf016]), Rehabilitaion Clinical Medical Centre of Yunnan Province (Grant numbers[zx2019-04-02]), National Key Research and Development Program (Grant numbers[2018YFC2002301]), and Jiajie Expert Workstation of Yunnan Province (Grant numbers[2019IC034]), and Kunming Medical University Postgraduate Innovation Fund (Grant numbers[2022B11]).

Powered by Editorial Manager® and ProduXion Manager® from Aries Systems Corporation

Did you receive funding for this work?"	
Please select the country of your main research funder (please select carefully as in some cases this is used in fee calculation).<br>as follow-up to "**Financial Disclosure**<br><br>Enter a financial disclosure statement that describes the sources of funding for the work included in this submission. Review the submission guidelines for detailed requirements. View published research articles from *PLOS ONE* for specific examples.<br><br>This statement is required for submission and **will appear in the published article** if the submission is accepted. Please make sure it is accurate.<br><br>**Funded studies**<br>Enter a statement with the following details:<br>• Initials of the authors who received each award<br>• Grant numbers awarded to each author<br>• The full name of each funder<br>• URL of each funder website<br>• Did the sponsors or funders play any role in the study design, data collection and analysis, decision to publish, or preparation of the manuscript?<br><br>Did you receive funding for this work?"	CHINA - CN
**Competing Interests**<br><br>Use the instructions below to enter a competing interest statement for this submission. On behalf of all authors, disclose any competing interests that could be perceived to bias this work—acknowledging all financial support and any other relevant financial or non-financial competing interests.<br><br>This statement is required for submission and **will appear in the published article** if	The authors have declared that no competing interests exist.

the submission is accepted. Please make sure it is accurate and that any funding sources listed in your Funding Information later in the submission form are also declared in your Financial Disclosure statement.<br><br>View published research articles from *PLOS ONE* for specific examples.<br><br>**NO authors have competing interests**<br><br>Enter: *The authors have declared that no competing interests exist.*<br><br>**Authors with competing interests**<br><br>Enter competing interest details beginning with this statement:<br><br>*I have read the journal's policy and the authors of this manuscript have the following competing interests: [insert competing interests here]*<br><br>* typeset	
**Ethics Statement**<br><br>Enter an ethics statement for this submission. This statement is required if the study involved:<br><br>• Human participants<br>• Human specimens or tissue<br>• Vertebrate animals or cephalopods<br>• Vertebrate embryos or tissues<br>• Field research<br><br>Write "N/A" if the submission does not require an ethics statement.<br><br>General guidance is provided below. Consult the submission guidelines for detailed instructions. **Make sure that all information entered here is included in the Methods section of the manuscript.**	Ethics Committee of the Second Affiliated Hospital of Kunming Medical University<br>PJ-2019-04

Powered by Editorial Manager® and ProduXion Manager® from Aries Systems Corporation

**Format for specific study types**<br><br>**Human Subject Research (involving human participants and/or tissue)**<br>• Give the name of the institutional review board or ethics committee that approved the study<br>• Include the approval number and/or a statement indicating approval of this research<br>• Indicate the form of consent obtained (written/oral) or the reason that consent was not obtained (e.g. the data were analyzed anonymously)<br><br>**Animal Research (involving vertebrate animals, embryos or tissues)**<br>• Provide the name of the Institutional Animal Care and Use Committee (IACUC) or other relevant ethics board that reviewed the study protocol, and indicate whether they approved this research or granted a formal waiver of ethical approval<br>• Include an approval number if one was obtained<br>• If the study involved *non-human primates*, add *additional details* about animal welfare and steps taken to ameliorate suffering<br>• If anesthesia, euthanasia, or any kind of animal sacrifice is part of the study, include briefly which substances and/or methods were applied<br><br>**Field Research**<br><br>Include the following details if this study involves the collection of plant, animal, or other materials from a natural setting:<br>• Field permit number<br>• Name of the institution or relevant body that granted permission	
**Data Availability**<br><br>Authors are required to make all data underlying the findings described fully available, without restriction, and from the time of publication. PLOS allows rare exceptions to address legal and ethical concerns. See the PLOS Data Policy and FAQ for detailed information.	Yes - all data are fully available without restriction

A Data Availability Statement describing where the data can be found is required at submission. Your answers to this question constitute the Data Availability Statement and **will be published in the article**, if accepted.<br><br>**Important:** Stating 'data available on request from the author' is not sufficient. If your data are only available upon request, select 'No' for the first question and explain your exceptional situation in the text box.<br><br>Do the authors confirm that all data underlying the findings described in their manuscript are fully available without restriction?	
**Describe where the data may be found in full sentences. If you are copying our sample text, replace any instances of XXX with the appropriate details.**<br><br>• If the data are **held or will be held in a public repository**, include URLs, accession numbers or DOIs. If this information will only be available after acceptance, indicate this by ticking the box below. For example: *All XXX files are available from the XXX database (accession number(s) XXX, XXX.).*<br>• If the data are all contained **within the manuscript and/or Supporting Information files**, enter the following: *All relevant data are within the manuscript and its Supporting Information files.*<br>• If neither of these applies but you are able to provide **details of access elsewhere**, with or without limitations, please do so. For example:<br><br>*Data cannot be shared publicly because of [XXX]. Data are available from the XXX Institutional Data Access / Ethics Committee (contact via XXX) for researchers who meet the criteria for access to confidential data.*<br><br>*The data underlying the results presented in the study are available from (include the name of the third party*	All relevant data are within the manuscript and its Supporting Information files.

and contact information or URL).<br>• This text is appropriate if the data are owned by a third party and authors do not have permission to share the data.<br><br>* typeset	
Additional data availability information:	

Manuscript
Click here to access/download;Manuscript;Manuscript - revised.docx

Mediation and moderation analysis of the association between physical capability and quality of life among stroke patients

Authors' Name:

Xue Yang (M.D.)[1,#], Hongmei Zhang (M.M.)[1,#], Yihuan Lu (M.M.)[1], Qian Liu (M.M.)[1], Liqing Yao (M.D.)[1,*]

Authors' Affiliation:

1 Department of rehabilitation medicine, the Second Affiliated Hospital of Kunming Medical University, No. 374, Dianmian Road, 650101 Kunming, Yunnan, China.

Authors' E-mail:

Xue Yang (M.D.): yangxuebetty@163.com

Hongmei Zhang (M.M.): zhm9803@163.com

Yihuan Lu (M.M.): liuqian20160821@163.com

Qian Liu (M.M.): luyihuan950717@163.com

Liqing Yao (M.D.): yaoliqing98731@163.com

First Author:

# Xue Yang (yangxuebetty@163.com) and Hongmei Zhang (zhm9803@163.com) contributed equally to this work.

Corresponding Author:

* Liqing Yao (E-mail: yaoliqing98731@163.com), Department of rehabilitation medicine, the Second Affiliated Hospital of Kunming Medical University, No. 374, Dianmian Road, 650101 Kunming, Yunnan, China.

Key words: stroke, mediation, moderation, self-efficacy, quality of life

Abstract

Purpose: To estimate the extent to which physical capacity, depressive symptoms, balanced self-efficacy (BSE), and other risk factors interrelated with stroke to influence quality of life (QoL) in stroke patients. A theoretical model based on Wilson and Cleary model tested the specific hypotheses: (1) physical capability has a direct and indirect effect on QoL mediated by BSE and depressive symptoms; (2) stroke risk factors (hypertension/diabetes/gender) moderate the above relationship.

Method: Six hundred and seventy stroke patients were enrolled from ten different hospitals at Yunnan province from 2019 to 2021. Patients' mental and physical function was assessed using Brunnstrom recovery stage (BRS), mini-balance evaluation system test (Mini-BEST), Barthel index (BI), Activities-specific Balance Confidence scale (ABC), and Hamilton depression scale (HAM-D). Structural equation model (SEM) was used to test the moderated mediation model in Mplus 8.0 software.

Results: The model showed good fit (RMSEA = 0.075, SRMR = 0.010). BSE significantly mediated the relationship between physical capability and QoL (β = 0.322, P = 0.002). Hypertension was found a significant moderator of all the direct paths from 'physical capability to QoL through depressive symptoms (B = 0.412, P = 0.015; B = 0.831, P = 0.020, respectively).

Conclusions: This study provides a better insight into the relationship between physical capability and QoL via BSE in stroke patients, which may help establish appropriate treatment for these individuals.

Key words: stroke, mediation, moderation, self-efficacy, quality of life

Introduction

Stroke is a primary cause of long-term neurological disability; there were >80 million stroke survivors worldwide in 2016 [1]. Compared to Europe, America, and Australia, Asia has the largest stroke burden, especially in China due to its large and aging population [2, 3]. Stroke decreases the survivors' quality of life (QoL) and therefore is a serious health problem in Asia [4]. The reduction in QoL often accompanies the

increased risk of lifestyle diseases, including hypertension, diabetes, and an array of physical dysfunctions. Thus, improving QoL can improve the overall health and physical ability of stroke survivors. Various factors, including physical [5] and psychical [6], affect the QoL of stroke survivors, which must be taken into account by rehabilitation professionals to improve the QoL.

A recent study showing a strong relationship between physical capability and QoL in stroke patients has raised serious concerns [7]. Physical capability denotes one's capacity to completely undertake a task. Normally, it is evaluated based on the assessment of an individual's balance and motor functions, such as the MiniBest scale [8], and Brunnstrom stages of stroke recovery [9]. Earlier research has showed that the better physical capacity is an important predictor of stroke survivors' QoL [5]. Additionally, physical capacity is associated with balance function [10], which may also pose risks to patients' QoL [11, 12].

Balance impairment could lead stroke patients to develop anxiety-related or depression-related distress behaviors such as fear of falling [13, 14]. Fear of falling could lower quality of life due to the sedentary lifestyle and physical disability after stroke [14]. Therefore, enhancing the balance confidence may improve patients' QoL [15]. Balance confidence can be assessed using the "Activities-specific Balance Confidence Scale (ABC)" questionnaire [16]. It also called Balance self-efficacy (BSE), which means an individual's belief in his or her ability to accomplish a task [17]. Studies in the last few decades have produced fairly convergent results ~~that~~ higher balance self-efficacy (BSE) ~~to be~~ linked to better stroke patients' QoL [18-20]. French et al. proposed a theoretical model to explain the mediating role of BSE on the relationship between the patient's physical capability and participation [17]. ~~Furthermore, participation has a direct relationship with QoL [21]~~. ~~Therefore, based on~~ the above theoretical model, ~~here,~~ we propose the first hypothesis, considering the mediation effect of BSE on the relationship between physical capability and QoL.

Additionally, poor QoL has been found strongly associated with severe depressive symptoms [22, 23]. Evidence suggests that depressive symptoms after stroke has a

negative impact on motor outcomes [24]. One research shows that there is negative relationship between depression and perception of quality of life [25]. Conversely, physical impairment contributes to post-stroke depressive disorders [26]. Thus, we propose the second hypothesis that the relationship between physical capabilities and QoL may also be mediated by depressive symptoms.

Besides, other risk factors such as hypertension, diabetes, and gender may further impact these relationships among physical capacity, depressive symptoms, BSE, and QoL [27, 28]. Hypertension is a leading stroke risk factor in Asia [3, 4], significantly affecting daily lives [29]. Hypertension with other comorbid conditions lowers the patient's QoL and physical capability [30, 31]. Interestingly, one study demonstrated that enhancing the self-efficacy of stroke patients can help them improve blood pressure [32]. Concerning diabetes, ischemic stroke is a well-known macrovascular complication of diabetes. A study reported that diabetic stroke patients have poorer outcomes than non-diabetic stroke patients [33]. Notably, one study indicated gender association with health-related QoL [33]. Therefore, this study also examined the effect of these moderators on the relationships among physical capability, BSE, depressive symptoms, and QoL. Accordingly, here, we propose the third hypothesis that hypertension/diabetes/gender may act as moderators affecting the mediated relationship in stroke patients [34].

To fully elucidate the complex relationships among factors that contribute to QoL in stroke, Wilson and Cleary's 1995 model (W-C model) is applied to link physical function (physical capability), general health perception (BSE, depressive symptoms) and QoL [35]. General health perception refers to self-reported health assessment [36]. A hypothetic and theoretic model of the expected moderated mediation is described in Fig. 1. The model tests specific hypotheses: (1) physical capability has a direct and indirect effect on QoL mediated by BSE and depressive symptoms; (2) stroke risk factors (hypertension/diabetes/gender) moderate the above relationship.

Methods

Study design

The research was a multicenter cross-sectional study conducted from April 2019 to April 2020. In total, 1,765 participants were recruited using the consecutive sampling method, consisting of inpatients from ten different rehabilitation institutions. Ethical approval was obtained from the local ethics committees (approval No. PJ-2019-04) and the research was performed according to the Declaration of Helsinki [37]. Written informed consent was obtained from all patients before participation.

Participants

Patients' participation criteria were as follows: (I) age, >18 years; (II) diagnosis of hemorrhagic or ischemic stroke for > 1 month; (III) stable vital signs, and (IV) signed informed consent. The participant exclusion criteria were as follows: (I) several mental disorders before stroke; (II) malignant tumors, and/or (III) dementia, loss of consciousness, or severe cardiopulmonary, liver, or kidney dysfunctions. Out of the 1,765 participants, 245 did not meet the inclusion criteria, and 850 were excluded based on exclusion criteria. Finally, 670 participants were included in the structural equation model analysis. A flow chart indicating the inclusion/exclusion of participants is shown in Fig. 2.

Procedure

Data were collected by four adequately trained data collectors. All participants had to complete relevant neurological assessments (demographic data, clinic data, neurologic and neuropsychologic function assessments) after they signed informed consent. Assessments outcomes and patient information were entered into the electronic database of our hospital department (Department of Rehabilitation, the Second Affiliated Hospitals of Kunming Medical University). The quality controls are explained in our previously published paper [10].

Measurement

Physical capability: The Brunnstrom recovery stage (BRS) [38] is conducted to evaluate post-stroke motor recovery in the affected limbs [9]. The evaluation consists of six levels, Stage 1 (flaccid paresis) to Stage 6 (approximately normal paresis). The total score is the summation of all scores of the limbs. A high score suggests a positive recovery. The Rasch reliability coefficient was above 0.90 [39]. Mini-BEST, a mini-

balance evaluation system test [8], assesses the dynamic balance of the patient. The mini-BESTest had high internal consistency and interrater reliability [40]. It includes 14 items, each rated from 0 (severe) to 2 (normal). The maximum score is 28 points. A higher score indicates better balance and postural control.

Quality of life (QoL): The Barthel index (BI) [41], a functionally independent tool, evaluates the patient's performance in daily life activities, with a total score ranging from 0 to 100. A high score indicates a better ability to live independently. BI demonstrates good validity and reliability [42].

Balance self-efficacy (BSE): ABC, an Activities-specific Balance Confidence scale, evaluates individuals' balance confidence while carrying out specific activities [43]. ABC shows excellent internal consistency and test-retest reliability [44]. This 11-point scale ranges from 0 (no confidence) to 100 (complete confidence). The total score denotes the average score.

Depressive symptoms: Hamilton depression scale (HAM-D) is widely used to assess the severity of depression [45]. HAM-D demonstrates satisfactory reliability [46]. It included 17 items that are scored on a scale of 0 to 4. A higher score suggests higher severity of depression.

Statistics analysis

Descriptive statistics and bivariate correlation analysis were conducted with IBM SPSS 22.0. Normal data distribution was confirmed from the normal probability plots. Continuous data, with normal or near normal distribution, are expressed as mean ± SD; median and inter-quartile ranges [P_{25}-P_{75}] are reported in case of nonnormal data distribution. Categorical data are expressed as rates and percentages. Pearson or Spearman correlation analyses were conducted to examine the correlations among BRS, Mini-BEST, QoL, BSE, and depressive symptoms.

The Mplus 8.0 program [47] was used to employ structural equation modeling (SEM) to test the hypothesized model. Some variables (<5%) had missing data. Missing data imputation used a process of multiple imputations assuming data missing at random [48]. The maximum likelihood estimation method was applied to assess the structural equation model. The goodness-of-fit indices are widely used for structural equation models [49]. $\chi^2/df \leq 5$ (the ratio of χ^2 to the degree of freedom) indicates an acceptable fitting of the data. The comparative five indexes (CFI) and the Tucker-Lewis index (TLI)

of > 0.95 represent an excellent model fit. The SRMR (standardized root mean square residual) and RMSEA (root mean square error of approximation) indices of ≤ 0.08 indicate an acceptable fit of the model. $P < 0.05$ denotes the statistical significance of the data.

Additionally, a recommended bias-corrected bootstrapped method was employed to examine the mediators of BSE and depressive symptoms on the relationship between physical capability and QoL, and generate 95% bias-corrected confidence intervals (CIs) [50], with the procedure of 5,000 samples. A multi-group comparison test was employed to examine the indirect effects of physical capacity and QoL differed between BSE and depressive symptoms. Also, the possible moderating effects of hypertension, diabetes, and gender among simple paths in the multiple-mediation model were examined. A statistically significant difference in the path coefficients between subgroups indicates the moderating effect of the path [34].

Results

Demographic characteristics

Six hundred and seventy participants (438 males, 232 females, mean age 61.4 ± 11.9 years) were involved in this study. The majority of participants (556, 83.0%) had an ischemic stroke, while the hemorrhagic and others were in 101 (15.1%) and 13 (1.9%) patients, respectively. Of these, 292 (43.6%) had a left stroke, and 297 (44.3%) had a right stroke. Patients with hypertension accounted for 68.5% (459), while patients with diabetes accounted for 11.3% (76). All clinical assessment scales are presented in Table 1.

Correlations

We found significant correlations between BRS, Mini-BEST, QoL, BSE, and depressive symptoms ($P < 0.001$) (Table 2). BRS showed positive correlation with Mini-BEST ($r = 0.696$), QoL ($r = 0.633$), BSE ($r = 0.644$), but was negatively correlated with depressive symptoms ($r = -0.438$). Additionally, Mini-BEST showed a significantly positive correlation with BSE, QoL ($r =0.789$, $r = 0.701$), but negative correlation with depressive symptoms ($r = -0.492$). BSE was positively correlated with QoL ($r = 0.758$) and negatively correlated with depressive symptoms ($r = -0.496$). QoL

was negatively associated with depressive symptoms (r = -0.535).

Fitting of the theoretical model

The SEM analysis showed that the study model fit indices indicated satisfactory fit (χ^2 = 14.233, χ^2/df = 4.744, P =0.002. RMSEA = 0.075, SRMR = 0.010, CFI = 0.994, TLI = 0.981).

Path analysis

As shown in Fig. 1, physical capability was the dependent variable; BSE and depressive symptoms were the mediators and QoL was the independent variable in the multiple mediation model. The standardized path coefficients, interpreted similarly to regression coefficients, are presented in Fig. 3. All the pathways between physical capability and QoL were significant in the model. This model explained a total of 66.4% of the variance in QoL. Physical capability was a positive predictive factor of QoL (β = 0.470, P < 0.000). BSE contributed most to the total variance of QoL (71.8%), while Depressive symptoms accounted for 31.6% of the variance of QoL. The mediator of BSE positively predicted QoL (β = 0.299, P < 0.000). However, depressive symptoms were the negative predictor of QoL (β = -0.122, P < 0.000). Additionally, physical capability positively predicted self-efficacy (β = 0.847, P < 0.000) but was a negative ~~predictive~~ of depressive symptoms (β = -0.562, P < 0.000) (Table 3).

Mediation effect analysis

As shown in Table 4, we found significant total, direct and indirect effects in the multiple mediation model. The specific indirect effect of physical capability on QoL via BSE was statistically significant (β = 0.254, P = 0.003). However, the other mediations of depressive symptoms were not statistically significant (β = 0.069, P = 0.136). These findings negated the hypothesis that depressive symptoms mediate the relationship between physical capability and QoL. Thus, BSE significantly mediate the association between physical capability and QoL (β = 0.322, P = 0.002).

Moderation effect analysis

The analysis presented in Table 5 shows that hypertension significantly moderated the indirect effect of physical capability on QoL (B = -0.953, P = 0.014). We examined the

moderation effect using the pick-a-point approach to estimate the conditional direct effects of physical capability on QoL via BSE and depressive symptoms on QoL at different levels of hypertension. The results indicated that the interaction terms (physical capability◊hypertension and depressive◊hypertension) in the pathways from physical capability to QoL through depressive symptoms were significant (B = 0.412, P = 0.015; B = 0.831, P = 0.020, respectively). The plots of the interactions between physical capability, depressive symptoms, and QoL are presented in Fig. 4a-c. In all, hypertension significantly moderates the association between physical capability and QoL toward depressive symptoms, supporting our hypothesis. However, diabetes and gender showed no significant effect on the indirect path (B = 0.943, P = 0.570; B = 0.022, P = 0.961, respectively). Thus, these findings do not support the hypothesis that diabetes/gender moderate the aforementioned association.

Discussion

This study primarily explored a multiple mediation model of the 'physical capability to QoL via BSE and depressive symptoms' pathway in a group of stroke patients from China based on the Wilson and Cleary model. Our findings are consistent with a previous study, showing the significant mediation effect of BSE on the relationship between physical capability and QoL [17]. As hypothesized, the physical capability showed an indirect positive correlation with QoL through BSE; a higher level of physical capability indicated higher BSE, thereby improving the patent's QoL.

However, we found no mediation effect of depressive symptoms on the same relationship. According to French et al. [16], depressive symptoms moderate the relationships between physical capability, self-efficacy, and participation in stroke patients. Although the hypothesis about depressive symptoms mediating the relationship between physical capability and QoL was not supported, all the paths in our model were significant. The present findings suggest that a high level of physical capability can predict the ~~high~~ severity of depressive symptoms and a better QoL, while a high severity of depressive symptoms predicts a lower QoL, which supported our

hypothesis.

Our results also demonstrated that hypertension moderated the relationship between physical capability, depressive symptoms, and QoL in stroke patients. The patients with hypertension had decreased physical capability, which in turn increased depressive symptoms. This result is consistent with Sok SR et al. showing a strong association between depressive symptoms and hypertension [33]. Accordingly, another study reported that depressed patients with hypertension had poor control of blood pressure [51]. It could be that antidepressant therapy interferes with blood pressure control in hypertension patients. Additionally, a recent study showed that increased walking (physical) activity lowers hypertension risk in postmenopausal women [52], which is consistent with our findings. Conversely, patients with high blood pressure were found less active than those without hypertension. Furthermore, QoL negatively correlates with stroke risk factors such as hypertension [53, 54]. In general, hypertension is an important moderator of relationships between physical capability, depressive symptoms, and QoL.

We found no evidence that hypertension moderated the 'physical capability to QoL via BSE' pathway. However, there is a lack of literature examining the relationship between hypertension and BSE, which must be explored in both a qualitative and quantitative manner.

The theoretical model in this study was built based on previous model suggesting that BSE is a significant mediator and hypertension has a moderating effect on the 'physical capability to QoL through depressive symptoms' pathway. The mediating effect of BSE and the moderating effect of hypertension may help interpret the mechanisms of the relationship between physical capability and QoL. Our conceptual model facilitates the understanding of these associations to make targeted strategies promoting QoL and rehabilitation. Health professionals should incorporate interventions of enhancing balance confidence and the control of blood pressure in their managements to physical and mental rehabilitation.

Limitations

There are some limitations to our research. First, we did not consider the complex nature of the QoL index, which may complicate relationships among the various missing data of clinical variables. Second, we did not take into account the impact of stroke subtypes, a possible confounding parameter in this study. Third, the cross-sectional study design restricts the interpretation and generalization of our results. An additional longitudinal study may help verify the conclusions of this study.

Conclusion

This study provides a better insight into the relationship between physical capability and QoL via BSE in stroke patients, which may help establish appropriate treatment for these individuals. Our results uncover the underlying moderating effect of hypertension on relationships between physical capability, depressive symptoms, and QoL. An effective rehabilitation strategy can achieve concrete targets for stroke risk patients via BSE, improving their QoL [55].

Statements & Declarations

Acknowledgement The authors would like to thank all the participants in the study and MJEditor (www.mjeditor.com) for its linguistic assistance during the preparation of this manuscript.

Funding The work was supported by the Major Science and Technology Project of Yunnan Province (Grant numbers[2018zf016]), Rehabilitaion Clinical Medical Centre of Yunnan Province (Grant numbers[zx2019-04-02]), National Key Research and Development Program (Grant numbers[2018YFC2002301]), and Jiajie Expert Workstation of Yunnan Province (Grant numbers[2019IC034]), and Kunming Medical University Postgraduate Innovation Fund (Grant numbers[2022B11]).

Author Contributions All authors contributed to the study conception and design. Material preparation, data collection and analysis were performed by [Xue Yang], [Hongmei Zhang] and [Qian Liu]. Administrative support was conducted by [Liqing

Yao]. The first draft of the manuscript was written by [Xue Yang] and all authors commented on previous versions of the manuscript. All authors read and approved the final manuscript. Xue Yang and Hongmei Zhang contributed equally to this work.

Ethics approval Ethical approval was obtained from the Ethics Committee of the Second Affiliated Hospital of Kunming Medical University (approval No. PJ-2019-04) and the research was performed according to the Declaration of Helsinki.

Consent to participate Written informed consent was obtained from all patients before participation.

Reference

[1] Global, regional, and national burden of stroke, 1990-2016: a systematic analysis for the Global Burden of Disease Study 2016 [J]. Lancet Neurology 2019; 18: 439-458.

[2] Chen Z, Jiang B, Ru X, et al. Mortality of Stroke and Its Subtypes in China: Results from a Nationwide Population-Based Survey [J]. Neuroepidemiology 2017; 48: 95-102.

[3] Kim AS, Cahill E and Cheng NT. Global Stroke Belt: Geographic Variation in Stroke Burden Worldwide [J]. Stroke 2015; 46: 3564-3570.

[4] Turana Y, Tengkawan J, Chia YC, et al. Hypertension and stroke in Asia: A comprehensive review from HOPE Asia [J]. J Clin Hypertens (Greenwich) 2021; 23: 513-521.

[5] Nordin N. A. M, Aziz N. A. A., Sulong S, et al. Functional limitation and health related quality of life and associated factors among long term stroke survivors in a Malaysian community [J]. Med J Malaysia 2016; 71: 313-321.

[6] Haghgoo HA, Pazuki ES, Hosseini AS, et al. Depression, activities of daily living and quality of life in patients with stroke [J]. J Neurol Sci 2013; 328: 87-91.

[7] Muren MA, Hutler M and Hooper J. Functional capacity and health-related quality of life in individuals post stroke [J]. Top Stroke Rehabil 2008; 15: 51-58.

[8] Franchignoni F, Horak F, Godi M, et al. Using psychometric techniques to improve the Balance Evaluation Systems Test: the mini-BESTest [J]. J Rehabil Med 2010; 42: 323-331.

[9] Naghdi S, Ansari NN, Mansouri K, et al. A neurophysiological and clinical study of Brunnstrom recovery stages in the upper limb following stroke [J]. Brain injury 2010; 24: 1372-1378.
[10] Yang X, Fu YT, Fan H, et al. Exploring the complex pathways between somatosensory impairment, physical ability and affective symptoms following stroke: mediation analyses [J]. Annals of Palliative Medicine 2022; 11: 2395-2409.
[11] Di Monaco M, Trucco M, Di Monaco R, et al. The relationship between initial trunk control or postural balance and inpatient rehabilitation outcome after stroke: a prospective comparative study [J]. Clinical rehabilitation 2010; 24: 543-554.
[12] Schmid AA, Van Puymbroeck M, Altenburger PA, et al. Balance is associated with quality of life in chronic stroke [J]. Top Stroke Rehabil 2013; 20(4): 340-346.
[13] Schmid AA, Van Puymbroeck M, Knies K, et al. Fear of falling among people who have sustained a stroke: a 6-month longitudinal pilot study [J]. The American journal of occupational therapy : official publication of the American Occupational Therapy Association 2011; 65: 125-132.
[14] Schmid AA, Arnold SE, Jones VA, et al. Fear of falling in people with chronic stroke [J]. The American journal of occupational therapy : official publication of the American Occupational Therapy Association 2015; 69: 6903350020.
[15] Torkia C, Best KL, Miller WC, et al. Balance Confidence: A Predictor of Perceived Physical Function, Perceived Mobility, and Perceived Recovery 1 Year After Inpatient Stroke Rehabilitation [J]. Archives of physical medicine and rehabilitation 2016; 97: 1064-1071.
[16] French MA, Miller A, Pohlig RT, et al. Depressive Symptoms Moderate the Relationship Among Physical Capacity, Balance Self-Efficacy, and Participation in People After Stroke [J]. Physical therapy 2021; 101: 1-10.
[17] French MA, Moore MF, Pohlig R, et al. Self-efficacy Mediates the Relationship between Balance/Walking Performance, Activity, and Participation after Stroke [J]. Top Stroke Rehabil 2016; 23: 77-83.
[18] Liang YY, Wang L, Yang Y, et al. Association Between Behavioral Dysexecutive

Syndrome and the Health-Related Quality of Life Among Stroke Survivors [J]. Frontiers in psychiatry 2020; 11: 563930.
[19] Hellstrom K, Lindmark B, Wahlberg B, et al. Self-efficacy in relation to impairments and activities of daily living disability in elderly patients with stroke: a prospective investigation [J]. J Rehabil Med 2003; 35: 202-207.
[20] Szczepanska-Gieracha J and Mazurek J. The Role of Self-Efficacy in the Recovery Process of Stroke Survivors [J]. Psychol Res Behav Manag 2020; 13: 897-906.
[21] van Mierlo ML, van Heugten CM, Post MW, et al. Quality of Life during the First Two Years Post Stroke: The Restore4Stroke Cohort Study [J]. Cerebrovascular diseases (Basel, Switzerland) 2016; 41: 19-26.
[22] Koleck M, Gana K, Lucot C, et al. Quality of life in aphasic patients 1 year after a first stroke [J]. Quality of life research : an international journal of quality of life aspects of treatment, care and rehabilitation 2017; 26: 45-54.
[23] Lin WQ, Huang TY, Liu L, et al. Prevalence and related factors of depression and falls among the elderly living in rural communities of Guangzhou [J]. Psychology, health & medicine 2020; 25: 980-988.
[24] Lin C, Babiker A, Srdanovic N, et al. Depressive symptoms after stroke are associated with worse recovery [J]. International journal of psychiatry in medicine 2020; 55: 227-238.
[25] León LPd, Mangin JPL and Ballesteros S. Psychosocial Determinants of Quality of Life and Active Aging. A Structural Equation Model [J]. International Journal of Environmental Research and Public Health 2020; 17, 6023.
[26] Robinson RG, Kubos KL, Price TR. A Two-Year Longitudinal Study of Post-Stroke Mood Disorders: Findings During the Initial Evaluation [J]. Stroke 1983; 14: 736-741.
[27] Heiland EG, Welmer AK, Wang R, et al. Cardiovascular Risk Factors and the Risk of Disability in Older Adults: Variation by Age and Functional Status [J]. J Am Med Dir Assoc 2019; 20: 208-212 e203.
[28] Tusa BS, Geremew BM and Tefera MA. Heath related quality of life and associated factors among adults with and without diabetes in Adama city East Shewa, Ethiopia

2019; using generalized structural equation modeling [J]. Health Qual Life Outcomes 2020; 18: 83.
[29]Li ZH, Lv YB, Kraus VB, et al. Trends in the Incidence of Activities of Daily Living Disability Among Chinese Older Adults From 2002 to 2014 [J]. J Gerontol A Biol Sci Med Sci 2020; 75: 2113-2118.
[30]Qian J and Ren X. Association between comorbid conditions and BADL/IADL disability in hypertension patients over age 45: Based on the China health and retirement longitudinal study (CHARLS) [J]. Medicine (Baltimore) 2016; 95: e4536.
[31]Katan M and Luft A. Global Burden of Stroke [J]. Seminars in neurology 2018; 38: 208-211.
[32]Zhang XP, Pan JH, Wan LH, et al. Factors influencing health behaviour, blood pressure control, and disability in hypertensive ischaemic stroke patients after a comprehensive reminder intervention [J]. J Adv Nurs 2020; 76: 1384-1393.
[33]Sok SR, Yim E, Sim H, et al. Relationships among Self-Care Competency, Presence of Depressive Symptom, and Health-Related Quality of Life of Korean Stroke Patients [J]. Clin Nurs Res 2021; 30: 670-679.
[34]Preacher KJ, Rucker DD, Hayes AF. Addressing moderated mediation hypotheses. Theory, methods, and prescriptions [J]. Multivariate Behav Res 2007; 42: 185-227.
[35]Wilson BI, Cleary PD. Linking Clinical Variables With Health-Related Quality of Life [J]. JAMA 1995; 273: 59-65.
[36]Saengsiri AO and Hacker ED. Conducting quality of life research in people with coronary artery disease in non-English-speaking countries: conceptual and operationalization issues [J]. J Cardiovasc Nurs 2015; 30: 74-84.
[37]Association WM. World Medical Association Declaration of Helsinki Ethical Principles for Medical Research Involving Human Subjects [J]. JAMA 2013; 310: 2191-2194.
[38]Sawner K JL. *Brunnstrom movement therapy in hemiplegia: a neurophysilogical approach. 2nd ed.* Philadelphia: JB Lippincott, 1992.
[39]Huang CY, Lin GH, Huang YJ, et al. Improving the utility of the Brunnstrom

recovery stages in patients with stroke: Validation and quantification [J]. Medicine (Baltimore) 2016; 95: e4508.
[40] Tsang CS, Liao LR, Chung RC, et al. Psychometric properties of the Mini-Balance Evaluation Systems Test (Mini-BESTest) in community-dwelling individuals with chronic stroke [J]. Physical therapy 2013; 93: 1102-1115.
[41] Florence I. Mahoney DWB. Functional evaluation: the Barthel index [J]. Maryland State Medical Journal 1965; 14: 56-61.
[42] Collin C, Wade DT, Davies S, et al. The Barthel ADL Index: a reliability study [J]. Int Disabil Srudies 1985; 10: 61-63.
[43] Powell LE, Myers AM. The Activities-specific Balance Confidence (ABC) Scale [J]. Journal of Gerontology 1995; 50A: M28-M34.
[44] Zhang D, Tian F, Gao W, et al. The Chinese Short Version of the Activities-Specific Balance Confidence Scale: Its Validity, Reliability, and Predictive Value for Future Falls in Community-Dwelling Older Adults [J]. Clin Interv Aging 2022; 17: 1483-1491.
[45] Fava GA, Kellner R, Munari F, et al. The Hamilton Depression Rating Scale in normals and depressives [J]. Acta psychiatrica Scandinavica 1982; 66: 26-32.
[46] Mu¨ller MJ, Dragicevic A. Standardized rater training for the Hamilton Depression Rating Scale (HAMD-17) in psychiatric novices [J]. Journal of affective disorders 2003; 77: 65 – 69.
[47] Muthen LK, Muthen BO. *Mplus User's Guide*. Los Angeles: CA: Muth´en & Muth´en, 2012.
[48] Yiran Dong C-YJP. Principled missing data methods for researchers [J]. SpringerPlus 2013; 2: 222.
[49] Hooper D, Coughlan J, Mullen MR. Structural Equation Modelling Guidelines for Determining Model Fit [J]. Journal of Business Research Methods 2008; 6: 53-60.
[50] Kline RB. *Principles and Practice of Structual Equation Modeling, 4th ed* 4th ed. New York: Guilford Press, 2016.
[51] Scalco AZ, Scalco MZ, Azul JBS, et al. Hypertension and depression [J]. Clinics 2005; 60: 241-250.

[52] Miller CR, Wactawski-Wende J, Manson JE, et al. Walking Volume and Speed Are Inversely Associated With Incidence of Treated Hypertension in Postmenopausal Women [J]. Hypertension 2020; 76: 1435-1443.
[53] Yao H, Zhang J, Wang Y, et al. Stroke risk and its association with quality of life: a cross-sectional study among Chinese urban adults [J]. Health Qual Life Outcomes 2021; 19: 236.
[54] Alotaibi SM, Alotaibi HM, Alolyani AM, et al. Assessment of the stroke-specific quality-of-life scale in KFHU, Khobar: A prospective cross-sectional study [J]. Neurosciences (Riyadh) 2021; 26: 171-178.
[55] Silva SM, Correa FI, Faria CD, et al. Reproducibility of the items on the Stroke Specific Quality of Life questionnaire that evaluate the participation component of the International Classification of Functioning, Disability and Health [J]. Disability and rehabilitation 2016; 38: 2413-2418.

Tables

Table 1. Demographic and clinical information

**Variables**	**N (%)/mean±SD**
**Age**	61.4 (11.9)
**Gender**	
Male	438 (65.4)
Female	232 (34.6)
**Time since stroke**	143.8 (530.2)
**Type of stroke**	
Ischemic	556 (83.0)
Hemorrhagic	101 (15.1)
Others	13 (1.9)
**Paretic side**	
Left	292 (43.6)
Right	297 (44.3)
Unknown	81 (12.1)
**Dysfunction**	
Limb functional disorder	571 (85.2)
Speech dysfunction	223 (33.3)
Cognitive disorder	15 (2.2)
Balance disorder	52 (7.8)
**Complication**	
Hypertension	459 (68.5)
Diabetes	76 (11.3)
Cancer	2 (0.3)
**Previous history**	
Operation history	6 (0.9)
Stroke history	153 (22.8)
**Assessment scales**	

MiniBest	24.17（7.247）
Brunnstrom	15.18 (3.346)
ABC	88.13（64.38 95.63）*
Barthel index	91.21（13.740）
HAM-D	3.67 (4.68)

ABC: Activity-specific Balance Confidence; HAM-D: Hamilton Depression Scale; * Median and inter-quartile range

Table 2. Correlations among variables

	**BRS**	**Mini-BEST**	**BSE**	**QoL**	**Depressive Symptoms**	**Mean**	**SD**
BRS	1					15.18	3.346
Mini-BEST	0.696**	1				24.17	7.247
BSE	0.644**◊	0.789**◊	1			76.90	25.61
QoL	0.633**	0.701**	0.758**	1		91.21	13.74
Depressive Symptoms	-0.438**	-0.492**	-0.496**	0.535**	1	3.67	4.68

***P* <0.001; ◊: Spearman correlation.

BRS: Brunnstrom; BSE: Balance Self-efficacy; QoL: Quality of Life; Mini-BEST: Mini-Balance Evaluation Systems Test; SD: Standard Deviation.

Table 3. Path analysis

**Variables**	**Point estimate**		**Product of coefficients**		***P***
	**B**	***β***	**SE**	**Z**	
Physical capability---> QoL	0.989	0.470	0.143	6.916	0.000**
Physical capability---> BSE	3.324	0.847	0.124	26.806	0.000**

Physical capability---> Depressive symptoms	-0.403	-0.562	0.026	-15.500	0.000**
BSE---> QoL	0.16	0.299	0.031	5.161	0.000**
Depressive symptoms---> QoL	-0.357	-0.122	0.087	-4.103	0.000**

B: Unstandardized coefficients; *β*: Standardized coefficients; SE: Standard error; QoL: Quality of Life; BSE: Balance Self-efficacy; ***P* <0.001.

Table 4. The total, direct and indirect effect

**Effects**	**Variables**	**Point estimate**		**Product of coefficients**		**Bias-corrected percentile method**		***P***
		**B**	***β***	**SE**	**Z**	**Lower bounds**	**Upper bounds**	
Total effect	Physical capability--->QoL	4.242	0.793	0.265	16.108	3.756	4.805	0.000**
Direct effect	Physical capability--->QoL	2.517	0.470	0.610	4.125	1.325	3.720	0.000**
Total indirect effect	Physical capability---> BSE--->QoL Physical capability--->Depressive symptoms--->QoL	1.725	0.322	0.559	3.086	0.693	2.887	0.002*

Specific indirect effect	Physical capability--->BSE--->QoL	1.357	0.254	0.456	2.976	0.497	2.291	0.003*
Specific indirect effect	Physical capability--->Depressive symptoms--->QoL	0.368	0.069	0.247	1.490	-0.069	0.891	0.136

B: Unstandardized coefficients; *β*: Standardized coefficients; SE: Standard error; QoL: Quality of Life; BSE: Balance Self-efficacy; **P*<0.05; ***P*<0.001.

Table 5. Moderated analysis

**Variables**	**Point estimate**	**Product of coefficients**		**Bias-corrected percentile method**		***P***
	**B**	**SE**	**Z**	**Lower bounds**	**Upper bounds**	
Hypertension	-0.953	0.404	-2.359	-1.793	-0.215	0.014*
Physical capability--->BSE	-0.956	0.925	-1.034	-2.765	0.844	0.301
BSE--->QoL	-0.192	0.103	-1.862	-0.406	0.192	0.063
Physical capability--->Depressive symptoms	0.412	0.169	2.442	0.08	0.132	0.015*
Depressive symptoms--->QoL	0.831	0.357	2.325	0.2	1.575	0.020*
Physical capability--->QoL	2.897	1.239	2.339	0.574	5.462	0.019*
Diabetes	0.943	1.660	0.568	-1.455	3.683	0.570

Gender	0.022	0.476	0.046	-0.835	1.015	0.961

B: Unstandardized coefficients; β: Standardized coefficients; SE: Standard error; QoL: Quality of Life; BSE: Balance Self-efficacy; *P<0.05.

Figure

Click here to access/download;Figure;Fig1.tif

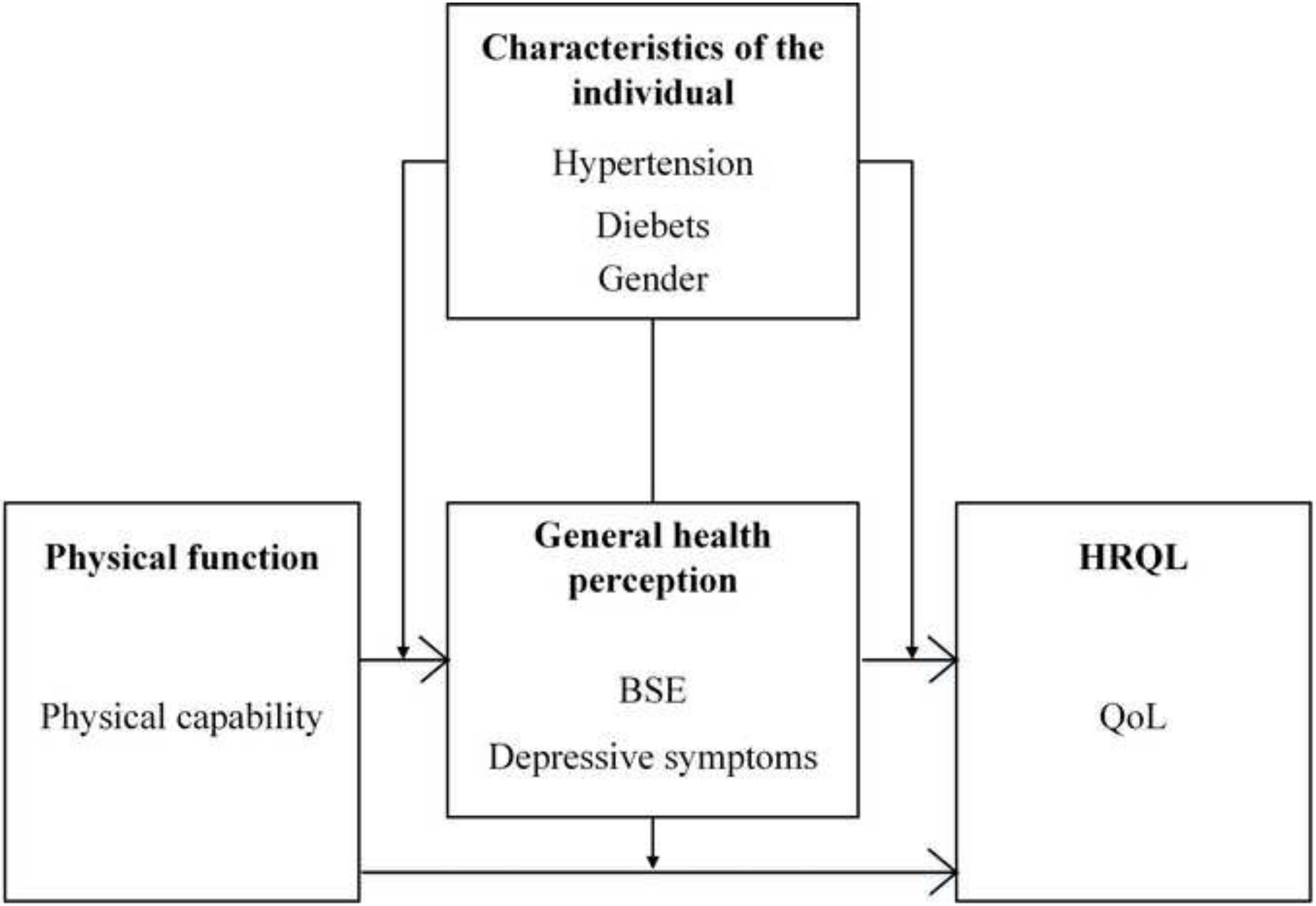

Fig. 1 The theoretical model based on Wilson and Cleary model. BSE: Balance self-efficacy; HROL: Health-related quality of life, QoL: Quality of Life. The arrows in the figure suggest the flow of the hypothesized influence of the variables in the study.

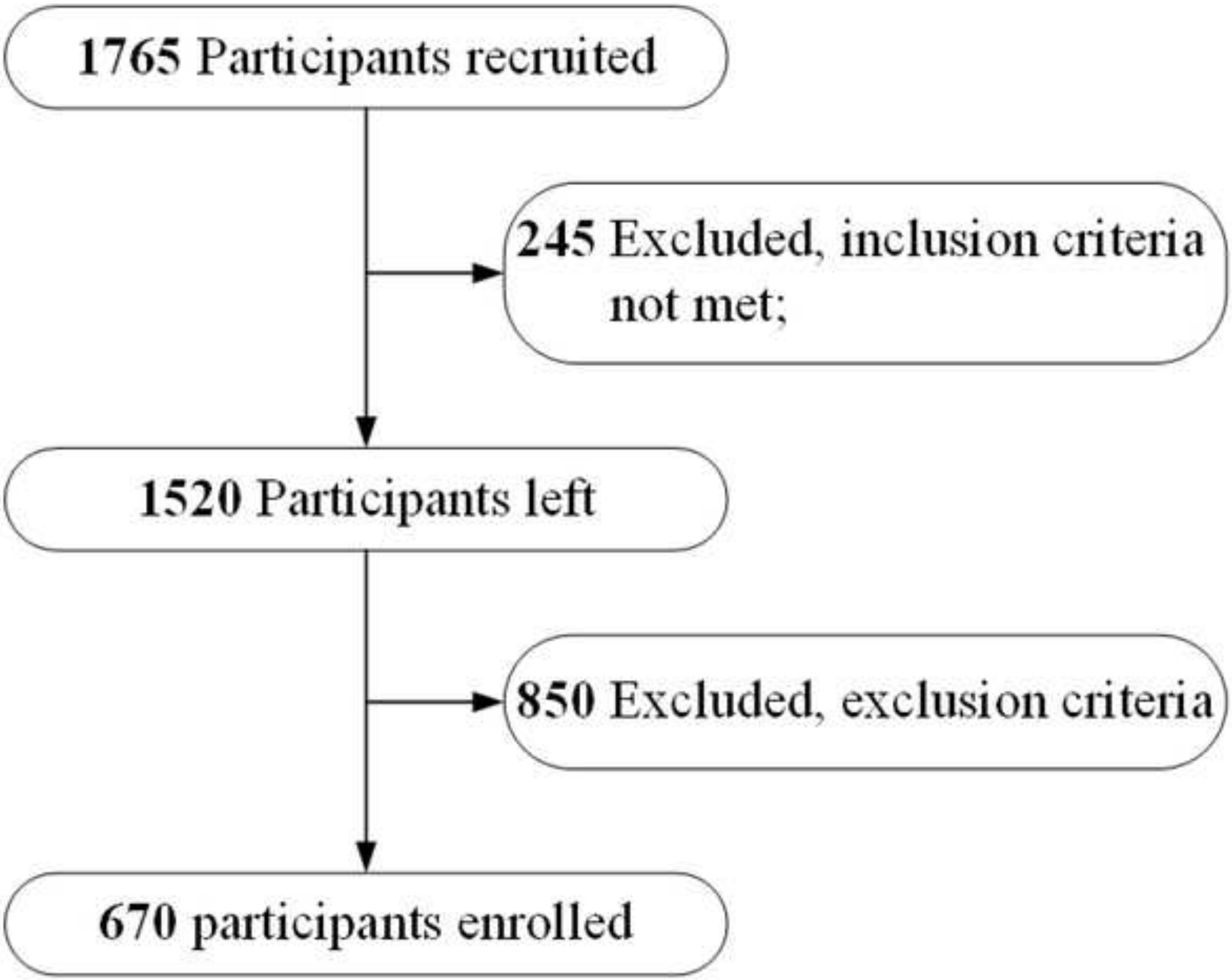

Fig. 2 Flow diagram.

 Click here to access/download;Figure;fig3.tif

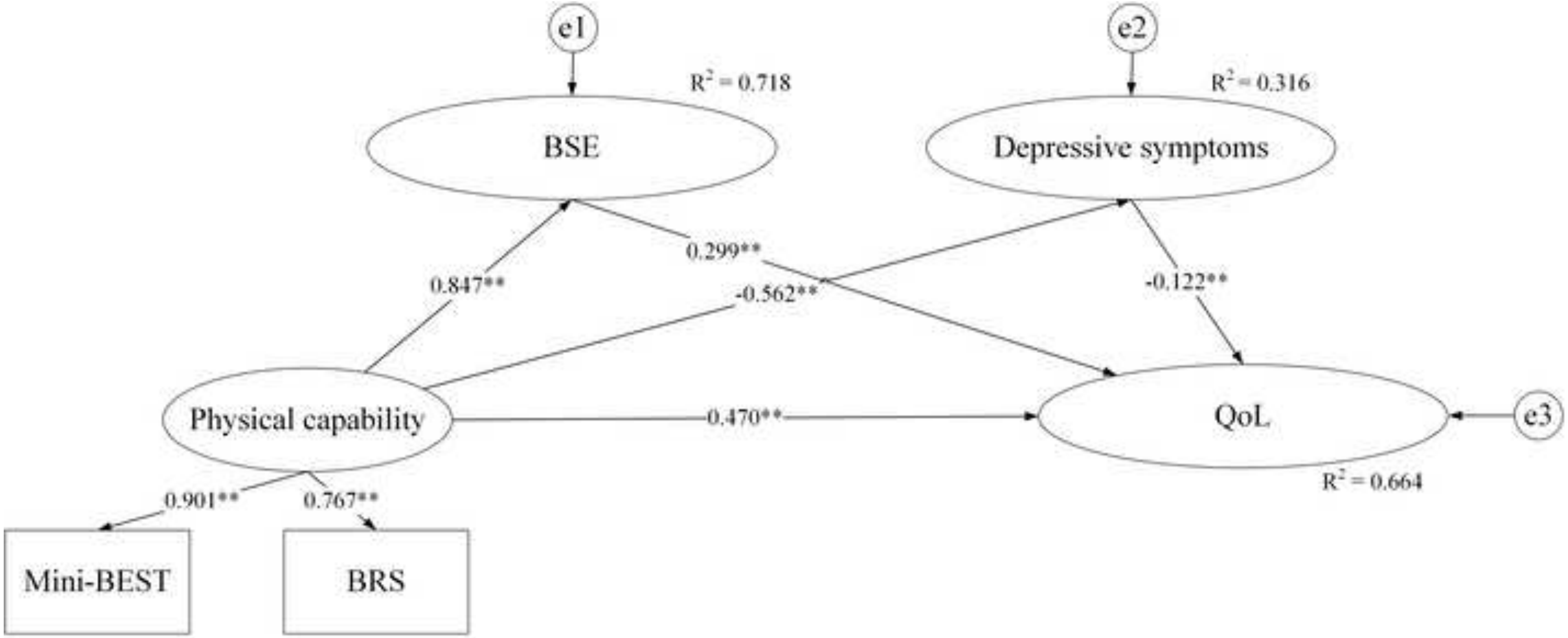

Fig. 3 Model fit (x^2=14.233, x^2/df=4.744, P=0.002. RMSEA=0.075, SRMR=0.010, TLI=0.981, CFI=0.994). Mini-BEST: Mini-Balance Evaluation Systems Test; BRS: Brunnstrom; BSE: Balance self-efficacy; QoL: Quality of Life.

 Click here to access/download;Figure;fig4.tif

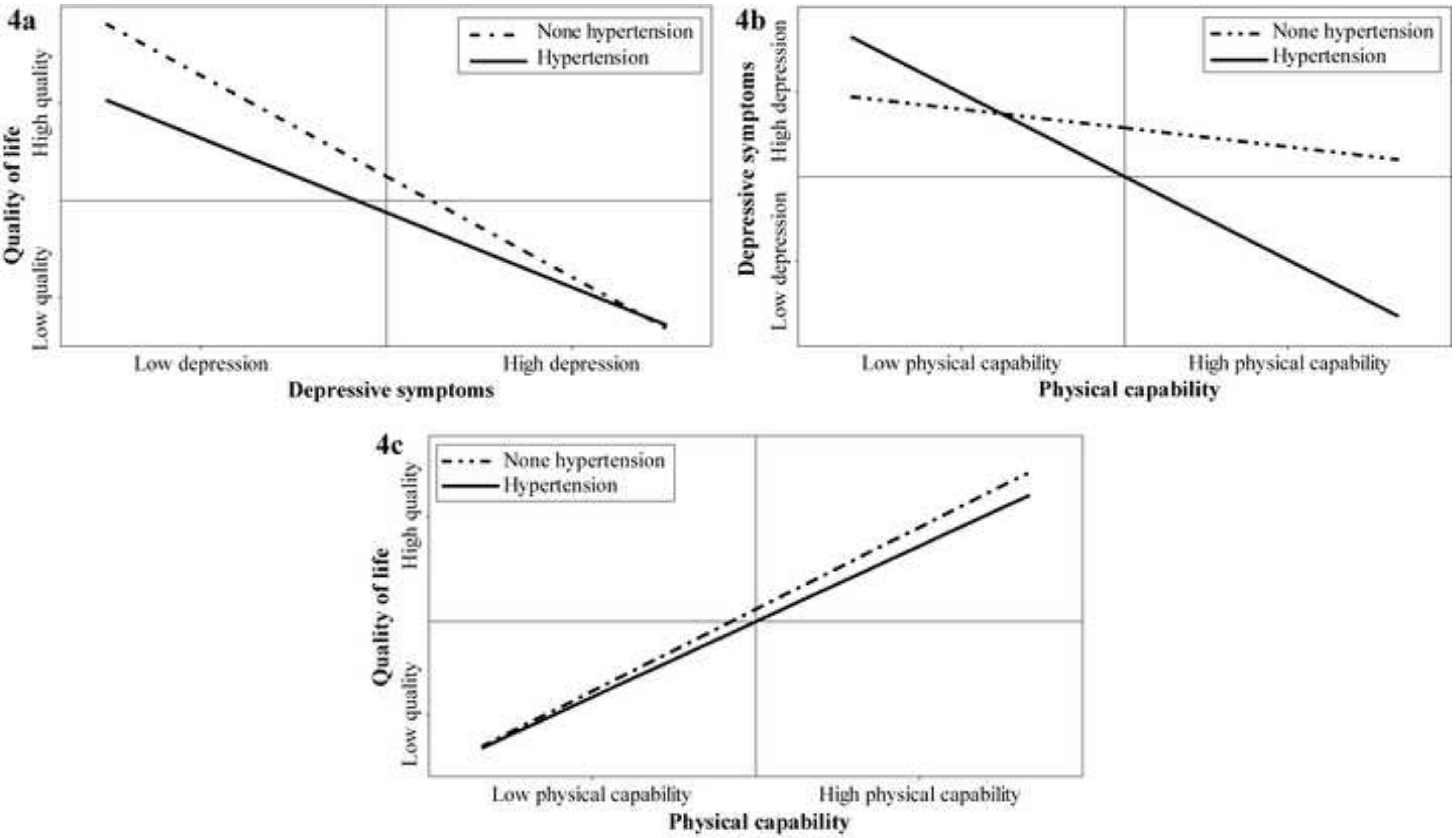

Fig. 4 Interactive effect of hypertension on the relationship between physical capability, quality of life and depressive symptoms.
