# Supplementary material for: Mediation and moderation analysis of the association between physical capability and quality of life among stroke patients
Source: PLoS One. 2025 Jun 10;20(6):e0320833. doi: 10.1371/journal.pone.0320833 (PMC12151468; doi:10.1371/journal.pone.0320833)
Supplement: Attachment — Submitted filename: PONE-D-24-45706_R1- reviewed.pdf [file pone.0320833.s005.pdf]

PLOS ONE

Mediation and moderation analysis of the association between physical capability and quality of life among stroke patients

--Manuscript Draft--

**Manuscript Number:**	PONE-D-24-45706R1
**Article Type:**	Research Article
**Full Title:**	Mediation and moderation analysis of the association between physical capability and quality of life among stroke patients
**Short Title:**	SEM of the association between physical capability and quality of life among stroke patients
**Corresponding Author:**	Liqing Yao<br>The Secomd Affiliated Hospital of Kunming Medical University<br>Kunming, CHINA
**Keywords:**	stroke, mediation, moderation, self-efficacy, quality of life
**Abstract:**	To estimate the extent to which physical capability, depressive symptoms, balanced self-efficacy (BSE), and other risk factors, that are interrelated with stroke, influence the quality of life (QoL) in stroke patients. A theoretical model based on Wilson and Cleary's model tested the specific hypotheses: 1) physical capability has an indirect effect on QoL mediated by BSE; 2) physical capability has an indirect effect on QoL by depressive symptoms; 3) stroke risk factors (hypertension/diabetes/gender) moderate the above relationship. Six hundred and seventy stroke patients were enrolled from ten different hospitals in Yunnan province from 2019 to 2021. Patients' mental and physical function was assessed using the Brunnstrom recovery stage (BRS), mini-balance evaluation system test (Mini-BEST), Barthel index (BI), Activities-specific Balance Confidence scale (ABC), and Hamilton depression scale (HAM-D). The structural equation model (SEM) was used to test the moderated mediation model in Mplus 8.0 software. The model showed a good fit (RMSEA = 0.075, SRMR = 0.010). BSE significantly mediated the relationship between physical capability and QoL (β = 0.322, p = 0.002). Hypertension was found a significant moderator of all the direct paths from physical capability to QoL through depressive symptoms (B = 0.412, p = 0.015; B = 0.831, p = 0.020, respectively). This study provides a better insight into the relationship between physical capability and QoL via BSE in stroke patients, which may help establish appropriate treatment for these individuals.
**Order of Authors:**	Liqing Yao
	Xue Yang
	hongmei Zhang
	qian Liu
	Yihuan Lu
**Response to Reviewers:**	Dear editors and reviewers:<br><br>Re: Manuscript ID: PONE-D-24-45706 and Title: Mediation and moderation analysis of the association between physical capability and quality of life among stroke patients<br><br>Thank you for your letter and the reviewers' comments concerning our manuscript entitled "PONE-D-24-45706" (ID). Those comments are valuable and very helpful. We have read through the comments carefully and have made corrections. Based on the instructions provided in your letter, we uploaded the file of the revised manuscript. Revisions in the main text are highlighted in yellow as additions or modifications. The responses to the reviewer's comments are marked in yellow and presented following.<br><br>We would love to thank you for allowing us to resubmit a revised copy of the manuscript and we highly appreciate your time and consideration.<br><br>Sincerely.

Powered by Editorial Manager® and ProduXion Manager® from Aries Systems Corporation

Liqing-Yao

Editorial opinion:
1) *If applicable, we recommend that you deposit your laboratory protocols in protocols.io to enhance the reproducibility of your results. Protocols.io assigns your protocol its identifier (DOI) so that it can be cited independently in the future. For instructions see: https://journals.plos.org/plosone/s/submission-guidelines#loc-laboratory-protocols. Additionally, PLOS ONE offers an option for publishing peer-reviewed Lab Protocol articles, which describe protocols hosted on protocols.io. Read more information on sharing protocols at https://plos.org/protocols?utm_medium=editorial-email&utm_source=authorletters&utm_campaign=protocols.*

Response: Thanks for the editorial suggestions.

Journal Requirements:

1)*Please ensure that your manuscript meets PLOS ONE's style requirements, including those for file naming. The PLOS ONE style templates can be found at

https://journals.plos.org/plosone/s/file?id=wjVg/PLOSOne_formatting_sample_main_body.pdf and https://journals.plos.org/plosone/s/file?id=ba62/PLOSOne_formatting_sample_title_authors_affiliations.pdf

Response: Thanks to the editorial advice, I have downloaded the PLOS ONE style template and have modified it in this way, e.g. the title page in the manuscript as well.

2)* Thank you for stating the following financial disclosure:

"The work was supported by the Major Science and Technology Project of Yunnan Province (Grant numbers[2018zf016]), Rehabilitation Clinical Medical Centre of Yunnan Province (Grant numbers[zx2019-04-02]), National Key Research and Development Program (Grant numbers[2018YFC2002301]), and Jiajie Expert Workstation of Yunnan Province (Grant numbers[2019IC034]), and Kunming Medical University Postgraduate Innovation Fund (Grant numbers[2022B11])."

Please state what role the funders took in the study. If the funders had no role, please state: "The funders had no role in study design, data collection and analysis, decision to publish, or preparation of the manuscript." If this statement is not correct you must amend it as needed.

Please include this amended Role of Funder statement in your cover letter; we will change the online submission form on your behalf.

Response: We have clarified the role of the funder in the study and have removed the funding section from the manuscript and added a funding section to the cover letter. This corresponds to lines 12 to 19 of the cover letter.

3)*Please note that funding information should not appear in the Acknowledgments section or other areas of your manuscript. We will only publish funding information present in the Funding Statement section of the online submission form. Please remove any funding-related text from the manuscript.

Response: We have removed any fund-related text from the manuscripts. We have added a funding section to the cover letter. This corresponds to lines 12 to 19 of the cover letter. This corresponds to lines 12 to 19 of the cover letter.

4)* We note that your Data Availability Statement is currently as follows:

“All relevant data are within the manuscript and its Supporting Information files.”

Please confirm at this time whether or not your submission contains all the raw data required to replicate the results of your study. Authors must share the “minimal data set” for their submission. PLOS defines the minimal data set to consist of the data required to replicate all study findings reported in the article, as well as related metadata and methods (https://journals.plos.org/plosone/s/data-availability#loc-minimal-data-set-definition).

For example, authors should submit the following data:

- The values behind the means, standard deviations, and other measures reported;
- The values used to build graphs;
- The points extracted from images for analysis.

Authors do not need to submit their entire data set if only a portion of the data was used in the reported study.

If your submission does not contain these data, please either upload them as Supporting Information files or deposit them to a stable, public repository and provide us with the relevant URLs, DOIs, or accession numbers. For a list of recommended repositories, please see https://journals.plos.org/plosone/s/recommended-repositories.

If there are ethical or legal restrictions on sharing a de-identified data set, please explain them in detail (e.g., data contain potentially sensitive information, data are owned by a third-party organization, etc.) and who has imposed them (e.g., an ethics committee). Please also provide contact information for a data access committee, ethics committee, or other institutional body to which data requests may be sent. If data are owned by a third party, please indicate how others may request data access.

Response: Thanks to the suggestion of the editorial team, we have uploaded the supporting data files named “data.xlsx” and “Mplus_moderation.xlsx”. The “data.xlsx” file is the full information of the 670 patients we included, while “Mplus_moderation.xlsx” is used to draw the patient information for structural equation modeling.

5)*PLOS requires an ORCID iD for the corresponding author in Editorial Manager on papers submitted after December 6th, 2016. Please ensure that you have an ORCID iD and that it is validated in Editorial Manager. To do this, go to ‘Update my Information’ (in the upper left-hand corner of the main menu), and click on the Fetch/Validate link next to the ORCID field. This will take you to the ORCID site and allow you to create a new ID or authenticate a pre-existing iD in Editorial Manager.

Response: Thanks to the editorial board's suggestion, we submitted this manuscript using the corresponding author's ORCID iD login to the PLOS ONE website.

6)*Your ethics statement should only appear in the Methods section of your manuscript. If your ethics statement is written in any section besides the Methods, please delete it from any other section.

Response: I have removed any part of the ethics statement written outside of the method.

7)* Please include a separate caption for each figure in your manuscript.

Response: I have captioned each figure separately in the manuscript as shown in Figure 1.

8)* Please review your reference list to ensure that it is complete and correct. If you have cited papers that have been retracted, please include the rationale for doing so in

the manuscript text, or remove these references and replace them with relevant current references. Any changes to the reference list should be mentioned in the rebuttal letter that accompanies your revised manuscript. If you need to cite a retracted article, indicate the article's retracted status in the References list and also include a citation and full reference for the retraction notice.

Response: I have checked the reference list.

Reviewer #1:
1)*The manuscript was well written. There are a few minor corrections to be made. These are in the track-changed document attached.
It will be great to discuss how stroke affects the quality of life of stroke survivors. Also, the results on BRS, MiniBest, QoL, BSE, and depression have to be presented first before the correlations.

Response: We thank the reviewers for their suggestions and we have revised this section.

Reviewer #2:
1)*Throughout the document, 'capacity' and 'capability' are used interchangeably. Although these words do have the same meaning, it will be easier to read and process the information if the same term is used throughout (I would recommend using 'capability' as indicated in the title). If the researchers did not intend to use the words as synonyms, it would be helpful to include a definition or description of the difference between the terms in the introduction section.

Response: I thank the reviewers for their careful suggestions. I have replaced "physical capacity" with "physical capability" throughout the manuscript and highlighted the changes in yellow.

2)*In the abstract, under the results heading there is a ' before physical capability (last sentence of the results section). Please either remove the ' or complete the statement by adding an ' after depressive symptoms.

Response:
I would like to thank the reviewers for their careful reading. I have revised and highlighted the text in yellow. This corresponds to lines 44 to 46 of the manuscript.

3)*In the introduction section, the first sentence is a bit difficult to read because of the punctuation used, my suggestion would be to change the sentence to: Stroke is a primary cause of long-term neurological disability, with >80 million stroke survivors globally in 2016. (so remove the , and change worldwide to globally to enhance the flow of the sentence).

Response: Thank you to the reviewers for their honest advice. I have revised and highlighted the text in yellow. This corresponds to lines 50 to 51 of the manuscript.

4)*In the second paragraph in the introduction (balance), the second sentence is a bit unclear (i.e., is the sedentary lifestyle a result of the stroke or as a result of the fear of falling)? Please rephrase for clarity.

Response: I would like to thank the reviewers for their careful reading. I have revised and highlighted the text in yellow. This corresponds to lines 71 to 73 of the manuscript.

5) *In the balance paragraph, in the sentence after the ABC questionnaire, please change it to it is (i.e., it is also called)

Response: I would like to thank the reviewers for their careful reading. I have revised and highlighted the text in yellow. This corresponds to lines 75 to 76 of the manuscript.

6)*Please revise the sentence in the balance paragraph (after reference nr 17) for ease of reading, my suggestion would be: Studies in the last few decades have produced fairly convergent results, indicating that higher balance self-efficacy is linked to better QOL of stroke patients.

Response: I would like to thank the reviewers for their careful reading. I have revised and highlighted the text in yellow. This corresponds to lines 77 to 79 of the manuscript.

7)*In the sentence after reference 21, please remove the word 'here'.

Response: I would like to thank the reviewers for their careful reading. I have revised and highlighted the text in yellow. This corresponds to lines 81 to 82 of the manuscript.

8)*In the first sentence on page 11 (after reference nr 24), please change to 'One research study'. In the following sentence, please change contributes to contribute.

Response: I would like to thank the reviewers for their careful reading. I have revised and highlighted the text in yellow. This corresponds to line 87 of the manuscript.

9)*In the introductory paragraph, regarding hypertension, please elaborate a bit more in terms of what you mean by 'significantly affecting daily lives' - are these activities of daily living?

Response: We thank the reviewers for their careful suggestions, and we have revised them here. This corresponds to lines 92 to 93 of the manuscript.

10)*At the conclusion of the introduction section, the information is a bit confusing (although I do see that you merged hypothesis one and two and reported it as one) - but this can be very confusing. I would recommend that you either adhere to the two hypotheses (as indicated at the end of the introduction section throughout) or that you write out all three (without merging the described hypotheses 1 and 2). Whichever you decide, please remember to adapt the abstract accordingly.

Response: We thank the reviewers for their careful suggestions, and we have revised them here. We have added hypothesis 3. This corresponds to lines 109 to 112 of the manuscript.

11)*In Figure 1, it would be nice to add either color or a bit more information, so that it is clear which factors are hypothesized to be mediators and which factors are moderators (to align more clearly with the written information).

Response: We thank the reviewers for their careful suggestions and we have revised Figure 1. This corresponds to lines 113 to 118 of the manuscript.

Powered by Editorial Manager® and ProduXion Manager® from Aries Systems Corporation

12)*Regarding the exclusion criteria, please be a bit more specific regarding 'several mental disorders' - are these disorders classified on the DSM, and how many were considered to be too many (for example, if a patient had anxiety and depression were they excluded, or were more than 2 diagnoses required for exclusion?). Were previous disability excluded as well? For example, if the patient had an amputation / severe orthopedic condition? (as these factors may influence the results of the study)

Response: I thank the reviewers for their honest advice. I have revised the exclusion criteria and highlighted the text in yellow. We refined the inclusion-exclusion criteria. This corresponds to lines 130 to 136 of the manuscript.

13)*Figure 2 does not indicate any additional information from what was provided in the text and the researchers can consider omitting it from the manuscript.

Response: Thank you to the reviewers for their suggestions. Since we have described the participant inclusion process in the Participants section of the methodology, the original Figure 2 has been removed.

14)*In the Procedure section (under the Methods heading) I referred to the referenced paper regarding quality controls, but it is not clear what the researchers are referring to - i.e., what quality controls were used? (This information is from the referenced study: Subsequently, 10 hospitals were invited to participate in relevant training programs every 3 months, and two trained investigators made irregular visits to different hospitals to collect information and conduct quality control. - is this what the researchers are referring to? Because it is not a lot of information, it might be useful to write it out in the current study (under review).

Response: I would like to thank the reviewers for their careful reading. We describe the data collection quality control process in detail. I have revised and highlighted the text in yellow. This corresponds to lines 140 to 147 of the manuscript.

15)*In the results section, please revise the second sentence, I suspect it is supposed to read: The majority of participants had an ischemic stroke, while hemorrhagic- and other strokes were reported in 101 and 13 patients respectively.

Response: I would like to thank the reviewers for their careful reading. I have revised and highlighted the text in yellow. This corresponds to lines 205 to 208 of the manuscript.

16)*Please clarify whether left stroke and right stroke refer to left/right hemiplegia/paresis or left or right cerebral involvement.

Response: Thank you to the reviewers for their suggestions. Left/right hemiparesis in the manuscript implies left/right paralysis or right/left stroke. This corresponds to lines 207 to 208 of the manuscript.

17)*Just for noting - I found the path analysis very interesting and Figure 3 assisted with interpreting and understanding all the information (which can sometimes be a bit overwhelming)

Response: Thank you to the reviewers for their suggestions. We have revised this figure and added a note to explain it. This corresponds to lines 247 to 253 of the manuscript.

18)*I think your interpretation of the diabetes/gender effect may be a bit harsh, i.e., that your hypothesis was rejected. I would recommend that you add that although the results from your study didn't support your hypothesis, the smaller (significantly so?) number of females (compared to males) and the small number of patients with diabetes may have influenced the results.

Response: We thank the reviewers for their suggestions and we add a note of clarification. This corresponds to lines 326 to 329 of the manuscript.

19)*In the discussion section, in the first sentence of the second paragraph (however, we found no...) please elaborate the sentence a bit more so that the 'relationship' you are referring to is clear.

Response: Thanks to the reviewer's suggestion, we have revised this sentence. This corresponds to lines 299 to 300 of the manuscript.

20)*Discussion section: When using in-text references, you don't have to include the initials (so it will become Sok et al.)

Response: I thank the reviewers for their honest advice. I have revised and highlighted the text in yellow. This corresponds to lines 311 to 312 of the manuscript.

21)*Please reference the sentence on page 17, i.e., 'it could be that antidepressant therapy interferes with blood pressure control in hypertension patients' - alternatively, if this is not a proven fact, indicate it (for example, the possibility exists that antidepressant therapy interferes with blood pressure control in hypertensive patients, but further research on the topic is warranted).

Response: We thank the reviewers for their suggestions and we apologize for the lack of rigor in the language of the manuscript. We have revised the language of the manuscript. This corresponds to lines 313 to 315 of the manuscript.

22)*The sentence directly after the one referred to in comment 21 needs revision - so that the link between postmenopausal women and the study becomes clear (for example: Additionally, a recent study showed that increased walking (physical) activity lowers hypertension risk in postmenopausal women [52]. Our study also indicated that physical activity positively influences hypertension risk, even in a mixed-gender population.

Response: Thanks to the reviewer's suggestion, we have revised this sentence. This corresponds to lines 317 to 318 of the manuscript.

23)*The final sentence of the discussion needs to be preceded by a sentence regarding balance so that the linkage becomes clear.

Response: Thanks to the careful suggestions of the reviewers, we have added a sentence on balanced functioning and QoL before the last sentence of the discussion to make the whole more logical and naturally articulated. This corresponds to lines 336 to 337 of the manuscript.

	24)*Throughout the discussion section OR (alternatively) in the limitations section, the researchers should make some recommendations for future research (either to say how limitations can be minimized in future research or recommendations for future research based on the findings of the study.<br><br>Response: We thank the reviewers for their suggestions and we have revised the limitations section. This corresponds to lines 341 to 351 of the manuscript.<br><br>25)*Please ensure the correct tense is used in the conclusion paragraph (the study has been conducted, so the section should be in the past tense).<br><br>Response: I thank the reviewers for their careful reading. I have modified the tense of the conclusion section to past tense. This corresponds to lines 353 to 358 of the manuscript.
Additional Information:	
Question	Response
**Financial Disclosure**<br><br>Enter a financial disclosure statement that describes the sources of funding for the work included in this submission. Review the submission guidelines for detailed requirements. View published research articles from *PLOS ONE* for specific examples.<br><br>This statement is required for submission and **will appear in the published article** if the submission is accepted. Please make sure it is accurate.<br><br>**Funded studies**<br>Enter a statement with the following details:<br>• Initials of the authors who received each award<br>• Grant numbers awarded to each author<br>• The full name of each funder<br>• URL of each funder website<br>• Did the sponsors or funders play any role in the study design, data collection and analysis, decision to publish, or preparation of the manuscript?<br><br>Did you receive funding for this work?	Yes
Please add funding details.<br>as follow-up to "**Financial Disclosure**<br><br>Enter a financial disclosure statement that	The work was supported by the Major Science and Technology Project of Yunnan Province (Grant numbers[2018zf016]), Rehabilitation Clinical Medical Centre of Yunnan Province (Grant numbers[zx2019-04-02]), National Key Research and Development Program (Grant numbers[2018YFC2002301]), and Jiajie Expert

Powered by Editorial Manager® and ProduXion Manager® from Aries Systems Corporation

describes the sources of funding for the work included in this submission. Review the submission guidelines for detailed requirements. View published research articles from *PLOS ONE* for specific examples.<br><br>This statement is required for submission and **will appear in the published article** if the submission is accepted. Please make sure it is accurate.<br><br>**Funded studies**<br>Enter a statement with the following details:<br>• Initials of the authors who received each award<br>• Grant numbers awarded to each author<br>• The full name of each funder<br>• URL of each funder website<br>• Did the sponsors or funders play any role in the study design, data collection and analysis, decision to publish, or preparation of the manuscript?<br><br>Did you receive funding for this work?"	Workstation of Yunnan Province (Grant numbers[2019IC034]), and Kunming Medical University Postgraduate Innovation Fund (Grant numbers[2022B11]). The funders had no role in study design, data collection and analysis, decision to publish, or preparation of the manuscript.
Please select the country of your main research funder (please select carefully as in some cases this is used in fee calculation).<br>as follow-up to "**Financial Disclosure**<br><br>Enter a financial disclosure statement that describes the sources of funding for the work included in this submission. Review the submission guidelines for detailed requirements. View published research articles from *PLOS ONE* for specific examples.<br><br>This statement is required for submission and **will appear in the published article** if the submission is accepted. Please make sure it is accurate.	CHINA - CN

**Funded studies**<br>Enter a statement with the following details:<br>• Initials of the authors who received each award<br>• Grant numbers awarded to each author<br>• The full name of each funder<br>• URL of each funder website<br>• Did the sponsors or funders play any role in the study design, data collection and analysis, decision to publish, or preparation of the manuscript?<br><br>Did you receive funding for this work?"	
**Competing Interests**<br><br>Use the instructions below to enter a competing interest statement for this submission. On behalf of all authors, disclose any competing interests that could be perceived to bias this work—acknowledging all financial support and any other relevant financial or non-financial competing interests.<br><br>This statement is required for submission and **will appear in the published article** if the submission is accepted. Please make sure it is accurate and that any funding sources listed in your Funding Information later in the submission form are also declared in your Financial Disclosure statement.<br><br>View published research articles from *PLOS ONE* for specific examples.	The authors have declared that no competing interests exist.

**NO authors have competing interests**<br><br>Enter: *The authors have declared that no competing interests exist.*<br><br>**Authors with competing interests**<br><br>Enter competing interest details beginning with this statement:<br><br>*I have read the journal's policy and the authors of this manuscript have the following competing interests: [insert competing interests here]*<br><br>* typeset	
**Ethics Statement**<br><br>Enter an ethics statement for this submission. This statement is required if the study involved:<br><br>• Human participants<br>• Human specimens or tissue<br>• Vertebrate animals or cephalopods<br>• Vertebrate embryos or tissues<br>• Field research<br><br>Write "N/A" if the submission does not require an ethics statement.<br><br>General guidance is provided below. Consult the submission guidelines for detailed instructions. **Make sure that all information entered here is included in the Methods section of the manuscript.**	Ethics Committee of the Second Affiliated Hospital of Kunming Medical University<br>PJ-2019-04

**Format for specific study types**<br><br>**Human Subject Research (involving human participants and/or tissue)**<br>• Give the name of the institutional review board or ethics committee that approved the study<br>• Include the approval number and/or a statement indicating approval of this research<br>• Indicate the form of consent obtained (written/oral) or the reason that consent was not obtained (e.g. the data were analyzed anonymously)<br><br>**Animal Research (involving vertebrate animals, embryos or tissues)**<br>• Provide the name of the Institutional Animal Care and Use Committee (IACUC) or other relevant ethics board that reviewed the study protocol, and indicate whether they approved this research or granted a formal waiver of ethical approval<br>• Include an approval number if one was obtained<br>• If the study involved *non-human primates*, add *additional details* about animal welfare and steps taken to ameliorate suffering<br>• If anesthesia, euthanasia, or any kind of animal sacrifice is part of the study, include briefly which substances and/or methods were applied<br><br>**Field Research**<br><br>Include the following details if this study involves the collection of plant, animal, or other materials from a natural setting:<br>• Field permit number<br>• Name of the institution or relevant body that granted permission	
**Data Availability**<br><br>Authors are required to make all data underlying the findings described fully available, without restriction, and from the time of publication. PLOS allows rare exceptions to address legal and ethical concerns. See the PLOS Data Policy and FAQ for detailed information.	Yes - all data are fully available without restriction

A Data Availability Statement describing where the data can be found is required at submission. Your answers to this question constitute the Data Availability Statement and **will be published in the article**, if accepted.<br><br>**Important:** Stating 'data available on request from the author' is not sufficient. If your data are only available upon request, select 'No' for the first question and explain your exceptional situation in the text box.<br><br>Do the authors confirm that all data underlying the findings described in their manuscript are fully available without restriction?	
**Describe where the data may be found in full sentences. If you are copying our sample text, replace any instances of XXX with the appropriate details.**<br><br>• If the data are **held or will be held in a public repository**, include URLs, accession numbers or DOIs. If this information will only be available after acceptance, indicate this by ticking the box below. For example: *All XXX files are available from the XXX database (accession number(s) XXX, XXX.)*.<br>• If the data are all contained **within the manuscript and/or Supporting Information files**, enter the following: *All relevant data are within the manuscript and its Supporting Information files.*<br>• If neither of these applies but you are able to provide **details of access elsewhere**, with or without limitations, please do so. For example:<br><br>*Data cannot be shared publicly because of [XXX]. Data are available from the XXX Institutional Data Access / Ethics Committee (contact via XXX) for researchers who meet the criteria for access to confidential data.*<br><br>*The data underlying the results presented in the study are available from (include the name of the third party*	All relevant data are within the manuscript and its Supporting Information files.

and contact information or URL).<br>• This text is appropriate if the data are owned by a third party and authors do not have permission to share the data.<br><br>* typeset	
Additional data availability information:	

Powered by Editorial Manager® and ProduXion Manager® from Aries Systems Corporation

Manuscript
Click here to access/download;Manuscript;Manuscript.docx

Mediation and moderation analysis of the association between physical capability and quality of life among stroke patients

Xue Yang [1¶], Hongmei Zhang [1¶], Yihuan Lu [1], Qian Liu [1], Liqing Yao [1*]

1 Department of Rehabilitation Medicine, the Second Affiliated Hospital of Kunming Medical University, Kunming, Yunnan, China

* Corresponding author
E-mail: yaoliqing98731@163.com(LY)

¶These authors contributed equally to this work.

Abstract

To estimate the extent to which physical capability, depressive symptoms, balanced self-efficacy (BSE), and other risk factors, that are interrelated with stroke, influence the quality of life (QoL) in stroke patients. A theoretical model based on Wilson and Cleary's model tested the specific hypotheses: 1) physical capability has an indirect effect on QoL mediated by BSE; 2) physical capability has an indirect effect on QoL by depressive symptoms; 3) stroke risk factors (hypertension/diabetes/gender) moderate the above relationship. Six hundred and seventy stroke patients were enrolled from ten different hospitals in Yunnan province from 2019 to 2021. Patients' mental and physical function was assessed using the Brunnstrom recovery stage (BRS), mini-balance evaluation system test (Mini-BEST), Barthel index (BI), Activities-specific Balance Confidence scale (ABC), and Hamilton depression scale (HAM-D). The structural equation model (SEM) was used to test the moderated mediation model in Mplus 8.0 software. The model showed a good fit (RMSEA = 0.075, SRMR = 0.010). BSE significantly mediated the relationship between physical capability and QoL (β = 0.322, p = 0.002). Hypertension was found a significant moderator of all the direct paths from physical capability to QoL through depressive symptoms (B = 0.412, p = 0.015; B = 0.831, p = 0.020, respectively). This study provides a better insight into the relationship between physical capability and QoL via BSE in stroke patients, which may help establish appropriate treatment for these individuals.

Introduction

Stroke is a primary cause of long-term neurological disability, with more than 80 million stroke survivors globally in 2016 [1]. Compared to Europe, America, and Australia, Asia has the largest stroke burden, especially in China due to its large and aging population [2, 3]. Stroke decreases the survivors' quality of life (QoL) because it

can cause physical and cognitive impairments, emotional distress, and fatigue[4, 5]. Additionally, it can also lead to social isolation and a loss of independence[6]. Therefore, it is a serious health problem worldwide, especially in Asia [7]. The reduction in QoL often accompanies the increased risk of lifestyle diseases, including hypertension, diabetes, and an array of physical dysfunctions. Thus, improving QoL can improve the overall health and physical ability of stroke survivors. Various factors, including physical [8] and psysical [9], affect the QoL of stroke survivors, which must be taken into account by rehabilitation professionals to improve the QoL.

A recent study showing a strong relationship between physical capability and QoL in stroke patients has raised serious concerns [10]. Physical capability denotes one’s capacity to completely undertake a task. Normally, it is evaluated based on the assessment of an individual’s balance and motor functions, such as the MiniBest scale [11], and Brunnstrom stages of stroke recovery [12]. Earlier research has shown that better physical capability is an important predictor of stroke survivors’ QoL [8]. Additionally, physical capability is associated with balance function [13], which may also pose risks to patients’ QoL [14, 15].

Balance impairment could lead stroke patients to develop anxiety-related or depression-related distress behaviors such as fear of falling [16, 17]. The fear of falling stroke survivors frequently leads to activity and participation limitations as a consequence of poststroke physical disability [17]. Therefore, enhancing the balance confidence may improve patients’ QoL [18]. Balance confidence can be assessed using the “Activities-specific Balance Confidence Scale (ABC)” questionnaire [19]. It is also called Balance self-efficacy (BSE), which means an individual’s belief in his or her ability to accomplish a task [20]. Studies in the last few decades have produced fairly convergent results, indicating that higher BSE is linked to better QoL of stroke patients [21-23]. French et al. proposed a theoretical model to explain the mediating role of BSE on the relationship between the patient's physical capability and participation [20]. Using the above theoretical model, we propose the first hypothesis, considering the mediation effect of BSE on the relationship between physical capability and QoL.

Additionally, poor QoL has been found ~~strongly~~ associated with severe depressive symptoms [24, 25]. Evidence suggests that depressive symptoms after stroke harm motor outcomes [26]. ~~One research study~~ that there is a negative relationship between depression and the perception of QoL [27]. Conversely, physical impairments contribute to post-stroke depressive disorders [28]. Thus, we propose the second hypothesis that the relationship between physical capabilities and QoL may also be mediated by depressive symptoms.

Besides, other risk factors such as hypertension, diabetes, and gender may further impact these relationships among physical capability, depressive symptoms, BSE, and QoL [29, 30]. Hypertension is a leading risk factor for stroke in Asia [3, 7], significantly increasing the risk of ADL disability [31]. Hypertension with other comorbid conditions lowers the patient's QoL and physical capability [32, 33]. Interestingly, one study demonstrated that enhancing the self-efficacy of stroke patients can help them improve blood pressure [34]. Concerning diabetes, ischemic stroke is a well-known macrovascular complication of diabetes. A study reported that diabetic stroke patients have poorer outcomes than non-diabetic stroke patients [35]. Notably, one study indicated gender association with health-related QoL [35]. Therefore, this study also examined the effect of these moderators (hypertension, diabetes, gender) on the relationships among physical capability, BSE, depressive symptoms, and QoL. Accordingly, here, we propose the third hypothesis that hypertension/diabetes/gender may act as moderators affecting the mediated relationship in stroke patients [36].

To fully elucidate the complex relationships among factors that contribute to QoL in stroke, Wilson and Cleary’s 1995 model (W-C model) is applied to link physical function (physical capability), general health perception (BSE, depressive symptoms), and QoL [37]. General health perception refers to self-reported health assessment [38]. A hypothetic and theoretic model of the expected moderated mediation is described in Fig 1. The model tests specific hypotheses: (1) physical capability has an indirect effect on QoL mediated by BSE; (2) physical capability has an indirect effect on QoL by depressive symptoms; (3) stroke risk factors (hypertension/diabetes/gender) moderate

the above relationship.

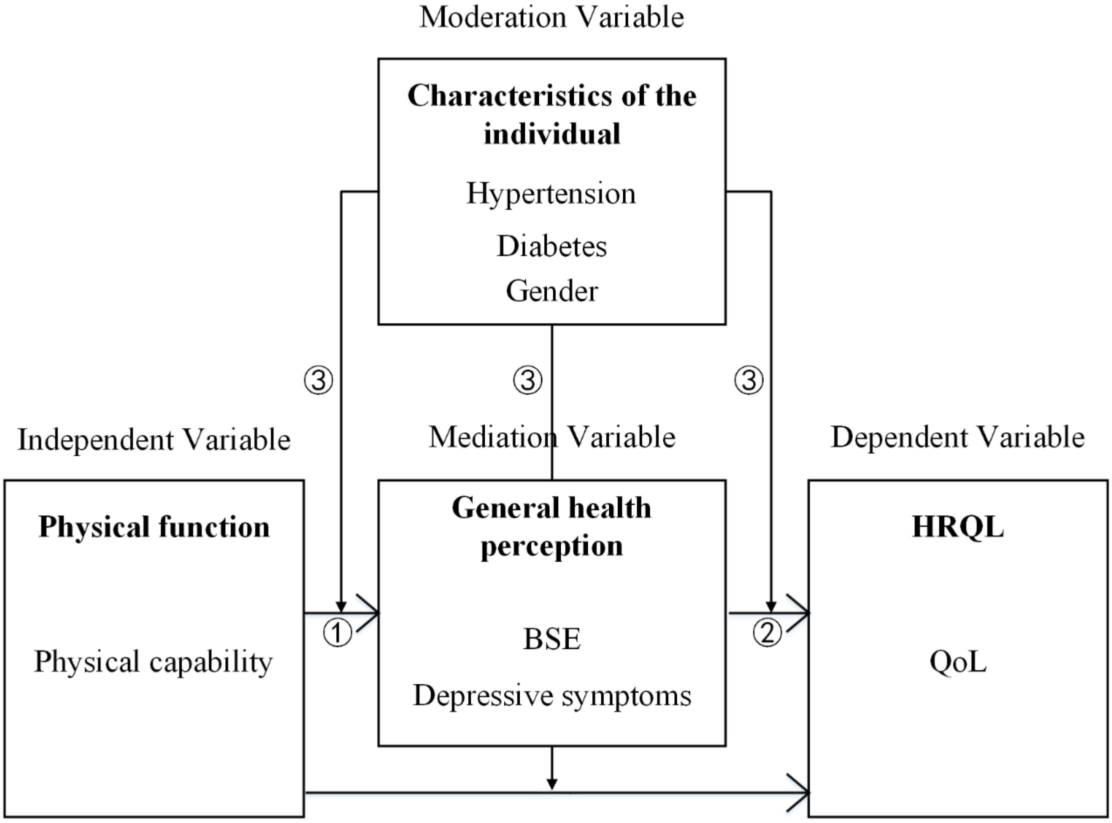

Fig 1. The theoretical model is based on Wilson and Cleary's model. ①: Hypothesis 1 of the current study; ②: Hypothesis 2 of the current study; ③: Hypothesis 3 of the current study. BSE: Balance Self-Efficacy; HRQL: Health-Related Quality of Life; QoL: Quality of Life. The arrows represent the hypothesized directional relationships between the variables in the model.

Methods

Study design

The research was a multicenter cross-sectional study conducted from April 2019 to April 2020. In total, 1,765 participants were recruited using the consecutive sampling method, consisting of inpatients from ten different rehabilitation institutions. Ethical approval was obtained from the local ethics committees (approval No. PJ-2019-04) and the research was performed according to the Declaration of Helsinki [39]. Written

informed consent was obtained from all patients before participation.

Participants

Patients' participation criteria were as follows: (I) age, >18 years; (II) diagnosis of hemorrhagic or ischemic stroke for > 1 month; (III) stable vital signs, and (IV) signed informed consent. The participant exclusion criteria were as follows: (I) presence of any mental diagnosis before stroke according to the Diagnostic and Statistical Manual of Mental Disorders classification (i.e., major depressive disorder, bi-directional affective disorder, and obsessive-compulsive disorder, etc.); (II) malignant tumors, and/or; (III) dementia, loss of consciousness, or severe cardiopulmonary, liver, or kidney dysfunctions; (IV) pre-existing physical disability from any cause, such as fracture, osteoarthritis, etc. Out of the 1,765 participants, 245 did not meet the inclusion criteria, and 850 were excluded based on exclusion criteria. Finally, 670 participants were included in the structural equation model analysis.

Procedure

The case data used in this cross-sectional study were obtained from stroke patients across ten rehabilitation facilities in Yunnan Province, China. Initially, ten hospitals were invited to participate in a training program conducted every three months. Subsequently, two trained researchers visited various hospitals periodically to collect data and perform quality control [13]. After patients provided informed consent, data collectors conducted a standardized assessment, which included a neurological evaluation covering demographic information, clinical data, and both neurological and neuropsychological functional assessments. Assessment outcomes and patient information were entered into the electronic database of our hospital department (Department of Rehabilitation, the Second Affiliated Hospitals of Kunming Medical University).

Measurement

Physical capability: The Brunnstrom recovery stage (BRS) [40] is conducted to

evaluate post-stroke motor recovery in the affected limbs [12]. The evaluation consists of six levels, Stage 1 (flaccid paresis) to Stage 6 (approximately normal paresis). The total score is the summation of all scores of the limbs. A high score suggests a positive recovery. The Rasch reliability coefficient was above 0.90 [41]. Mini-BEST, a mini-balance evaluation system test [11], assesses the dynamic balance of the patient. The mini-BEST had high internal consistency and interrater reliability [42]. It includes 14 items, each rated from 0 (severe) to 2 (normal). The maximum score is 28 points. A higher score indicates better balance and postural control.

Quality of life (QoL): The Barthel index (BI) [43], a functionally independent tool, evaluates the patient's performance in daily life activities, with a total score ranging from 0 to 100. A high score indicates a better ability to live independently. BI demonstrates good validity and reliability [44].

Balance self-efficacy (BSE): ABC, an Activities-specific Balance Confidence scale, evaluates individuals' balance confidence while carrying out specific activities [45]. ABC shows excellent internal consistency and test-retest reliability [46]. This 11-point scale ranges from 0 (no confidence) to 100 (complete confidence). The total score denotes the average score.

Depressive symptoms: The Hamilton depression scale (HAM-D) is widely used to assess the severity of depression [47]. HAM-D demonstrates satisfactory reliability [48]. It included 17 items that were scored on a scale of 0 to 4. A higher score suggests a higher severity of depression.

Statistics analysis

Descriptive statistics and bivariate correlation analysis were conducted with IBM SPSS 22.0. Normal data distribution was confirmed from the normal probability plots. Continuous data, with normal or near normal distribution, are expressed as mean ± SD; median and inter-quartile ranges [P_{25}-P_{75}] are reported in case of nonnormal data distribution. Categorical data are expressed as rates and percentages. Pearson or Spearman correlation analyses were conducted to examine the correlations among BRS, Mini-BEST, QoL, BSE, and depressive symptoms.

The Mplus 8.0 program [49] was used to employ structural equation modeling (SEM) to test the hypothesized model. Some variables (<5%) had missing data. Missing data

imputation uses a process of multiple imputations assuming data missing at random [50]. The maximum likelihood estimation method was applied to assess the structural equation model. The goodness-of-fit indices are widely used for structural equation models [51]. $\chi^2/df \leq 5$ (the ratio of χ^2 to the degree of freedom) indicates an acceptable fitting of the data. The comparative five indexes (CFI) and the Tucker-Lewis index (TLI) of > 0.95 represent an excellent model fit. The SRMR (standardized root mean square residual) and RMSEA (root mean square error of approximation) indices of ≤ 0.08 indicate an acceptable fit of the model. $p < 0.05$ denotes the statistical significance of the data.

Additionally, a recommended bias-corrected bootstrapped method was employed to examine the mediators of BSE and depressive symptoms on the relationship between physical capability and QoL and generate 95% bias-corrected confidence intervals (CIs) [52], with the procedure of 5,000 samples. A multi-group comparison test was employed to examine the indirect effects of physical capability and QoL differed between BSE and depressive symptoms. Also, the possible moderating effects of hypertension, diabetes, and gender among simple paths in the multiple-mediation model were examined. A statistically significant difference in the path coefficients between subgroups indicates the moderating effect of the path [36].

Results

Demographic characteristics

Six hundred and seventy participants (438 males, 232 females, mean age 61.4 ± 11.9 years) were involved in this study. The majority of participants (556, 83.0%) had an ischemic stroke, while hemorrhagic- and other strokes were reported in 101 (15.1%) and 13 (1.9%) patients respectively. Of the total number, 292 (43.6%) had a left-sided stroke (right hemiparesis), and 297 (44.3%) had a right-sided stroke (left hemiparesis). Patients with hypertension accounted for 68.5% (459), while patients with diabetes accounted for 11.3% (76).

Correlations

The median score of the ABC was 88.13, whereas the mean assessment scales of mini-BEST, BRS, BI, and HAM-D are outlined in Table 1. We found significant correlations between BRS, Mini-BEST, QoL, BSE, and depressive symptoms ($p < 0.001$) (Table 2). BRS showed a positive correlation with Mini-BEST (r = 0.696), QoL (r = 0.633), and BSE (r = 0.644), but was negatively correlated with depressive symptoms (r = -0.438). Additionally, Mini-BEST showed a significantly positive correlation with BSE, and QoL (r =0.789, r = 0.701), but a negative correlation with depressive symptoms (r = -0.492). BSE was positively correlated with QoL (r = 0.758) and negatively correlated with depressive symptoms (r = -0.496). QoL was negatively associated with depressive symptoms (r = -0.535).

Table 1. Demographic and clinical information.

**Variables**	**N (%)/mean±SD**
**Age, mean (SD)**	61.4 (11.9)
**Gender, N (%)**	
Male	438 (65.4)
Female	232 (34.6)
**Time since stroke, mean (SD)**	143.8 (530.2)
**Type of stroke, N (%)**	
Ischemic	556 (83.0)
Hemorrhagic	101 (15.1)
Others	13 (1.9)
**Paretic side, N (%)**	
Left	292 (43.6)
Right	297 (44.3)
Unknown	81 (12.1)
**Dysfunction, N (%)**	
Limb functional disorder	571 (85.2)

Speech dysfunction	223 (33.3)
Cognitive disorder	15 (2.2)
Balance disorder	52 (7.8)
**Complication, N (%)**	
Hypertension	459 (68.5)
Diabetes	76 (11.3)
Cancer	2 (0.3)
**Previous history, N (%)**	
Operation History	6 (0.9)
Stroke History	153 (22.8)
**Assessment scales, mean (SD)**	
Mini-BEST	24.17（7.247）
BRS	15.18 (3.346)
ABC, Median (inter-quartile range)	88.13（64.38 95.63）[a]
Barthel index	91.21（13.740）
HAM-D	3.67 (4.68)

ABC: Activity-specific Balance Confidence; HAM-D: Hamilton Depression Scale; Mini-BEST: Mini-Balance Evaluation Systems Test; BRS: Brunnstrom; [a]: Median and inter-quartile range.

Table 2. Correlations among variables.

	**BRS**	**Mini-BEST**	**BSE**	**QoL**	**Depressive Symptoms**	**Mean**	**SD**
**BRS**	1					15.18	3.346
**Mini-BEST**	0.696[aa]	1				24.17	7.247
**BSE**	0.644[aab]	0.789[aab]	1			76.90	25.61
**QoL**	0.633[aa]	0.701[aa]	0.758[aa]	1		91.21	13.74
**Depressive Symptoms**	-0.438[aa]	-0.492[aa]	-0.496[aa]	0.535[aa]	1	3.67	4.68

[aa]: p <0.001; [b]: Spearman correlation.

BRS: Brunnstrom; BSE: Balance Self-efficacy; QoL: Quality of Life; Mini-BEST: Mini-Balance Evaluation Systems Test; SD: Standard Deviation.

Fitting of the theoretical model

The SEM analysis showed that the study model fit indices indicated satisfactory fit ($\chi^2 = 14.233$, $\chi^2/df = 4.744$, p =0.002. RMSEA = 0.075, SRMR = 0.010, CFI = 0.994, TLI = 0.981).

Path analysis

As shown in Fig 1, physical capability was the independent variable; BSE and depressive symptoms were the mediators and QoL was the dependent variable in the multiple mediation model. The standardized path coefficients, interpreted similarly to regression coefficients, are presented in Fig 2. All the pathways between physical capability and QoL were significant in the model. This model explained a total of 66.4% of the variance in QoL. Physical capability was a positive predictive factor of QoL ($\beta = 0.470$, $p < 0.000$). BSE contributed most to the total variance of QoL (71.8%), while Depressive symptoms accounted for 31.6% of the variance of QoL. The mediator of BSE positively predicted QoL ($\beta = 0.299$, $p < 0.000$). However, depressive symptoms were the negative predictor of QoL ($\beta = -0.122$, $p < 0.000$). Additionally, physical capability positively predicted self-efficacy ($\beta = 0.847$, $p < 0.000$) but was a negative of depressive symptoms ($\beta = -0.562$, $p < 0.000$) (Table 3).

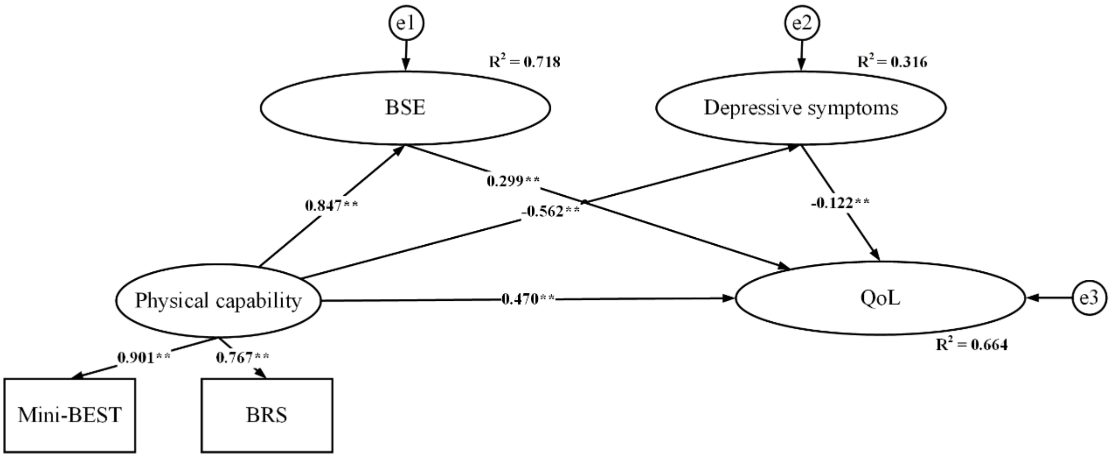

Fig 2. Model fit (χ^2 = 14.233, χ^2/df = 4.744, *p* = 0.002. RMSEA = 0.075, SRMR = 0.010, TLI = 0.981, CFI = 0.994). Mini-BEST: mini-Balance Evaluation Systems Test; BRS: Brunnstrom; BSE: Balance self-efficacy; QoL: Quality of Life. This model is consistent with hypotheses 1 and 2 of the theoretical model. BSE and Depressive symptoms are mediating variables between Physical capability and QoL.

Table 3. Path analysis.

**Variables**	**Point estimate**		**Product of coefficients**		***p***
	B	***β***	**SE**	**Z**	
Physical capability---> QoL	0.989	0.470	0.143	6.916	0.000 [aa]
Physical capability---> BSE	3.324	0.847	0.124	26.806	0.000 [aa]
Physical capability---> Depressive symptoms	-0.403	-0.562	0.026	-15.500	0.000 [aa]
BSE---> QoL	0.16	0.299	0.031	5.161	0.000 [aa]
Depressive symptoms---> QoL	-0.357	-0.122	0.087	-4.103	0.000 [aa]

B: Unstandardized coefficients; *β*: Standardized coefficients; SE: Standard error; QoL: Quality of Life; BSE: Balance Self-efficacy; [aa]: *p* <0.001.

Mediation effect analysis

As shown in Table 4, we found significant total, direct, and indirect effects in the multiple mediation model. The specific indirect effect of physical capability on QoL via BSE was statistically significant (*β* = 0.254, *p* = 0.003). However, the other mediations of depressive symptoms were not statistically significant (*β* = 0.069, *p* =

0.136). These findings negated the hypothesis that depressive symptoms mediate the relationship between physical capability and QoL. Thus, BSE significantly mediates the association between physical capability and QoL (β = 0.322, p = 0.002).

Table 4. The total, direct, and indirect effects.

**Effects**	**Variables**	**Point estimate**		**Product of coefficients**		**Bias-corrected percentile method**		***p***
		**B**	***β***	**SE**	**Z**	**Lower bounds**	**Upper bounds**	
Total effect	Physical capability--->QoL	4.242	0.793	0.265	16.108	3.756	4.805	0.000[aa]
Direct effect	Physical capability--->QoL	2.517	0.470	0.610	4.125	1.325	3.720	0.000[aa]
Total indirect effect	Physical capability---> BSE--->QoL; Physical capability--->Depressive symptoms--->QoL	1.725	0.322	0.559	3.086	0.693	2.887	0.002[a]
Specific indirect effect	Physical capability--->BSE--->QoL	1.357	0.254	0.456	2.976	0.497	2.291	0.003[a]
Specific indirect effect	Physical capability--->Depressive symptoms--->QoL	0.368	0.069	0.247	1.490	-0.069	0.891	0.136

B: Unstandardized coefficients; β: Standardized coefficients; SE: Standard error; Z: Z-

value; QoL: Quality of Life; BSE: Balance Self-efficacy; [a]: p<0.05; [aa]: p<0.001.

Moderation effect analysis

The analysis presented in Table 5 shows that hypertension significantly moderated the indirect effect of physical capability on QoL (B = -0.953, p = 0.014). We examined the moderation effect using the pick-a-point approach to estimate the conditional direct effects of physical capability on QoL via BSE and depressive symptoms on QoL at different levels of hypertension. The results indicated that the interaction terms (physical capability × hypertension and depressive × hypertension) in the pathways from physical capability to QoL through depressive symptoms were significant (B = 0.412, p = 0.015; B = 0.831, p = 0.020, respectively). The plots of the interactions between physical capability, depressive symptoms, and QoL are presented in Fig 3. In all, hypertension significantly moderates the association between physical capability and QoL toward depressive symptoms, supporting our hypothesis. However, diabetes and gender showed no significant effect on the indirect path (B = 0.943, p = 0.570; B = 0.022, p = 0.961, respectively). Thus, these findings do not support the hypothesis that diabetes/gender moderates the aforementioned association.

Table 5. Moderated analysis.

**Variables**	**Point estimate**	**Product of coefficients**		**Bias-corrected percentile method**		***p***
	B	**SE**	**Z**	**Lower bounds**	**Upper bounds**	
Hypertension	-0.953	0.404	-2.359	-1.793	-0.215	0.014 [a]
Physical capability-->BSE	-0.956	0.925	-1.034	-2.765	0.844	0.301

BSE--->QoL	-0.192	0.103	-1.862	-0.406	0.192	0.063
Physical capability--->Depressive symptoms	0.412	0.169	2.442	0.08	0.132	0.015[a]
Depressive symptoms--->QoL	0.831	0.357	2.325	0.2	1.575	0.020[a]
Physical capability--->QoL	2.897	1.239	2.339	0.574	5.462	0.019[a]
Diabetes	0.943	1.660	0.568	-1.455	3.683	0.570
Gender	0.022	0.476	0.046	-0.835	1.015	0.961

B: Unstandardized coefficients; *β*: Standardized coefficients; SE: Standard error; Z: Z-value; QoL: Quality of Life; BSE: Balance Self-efficacy; [a]: p<0.05.

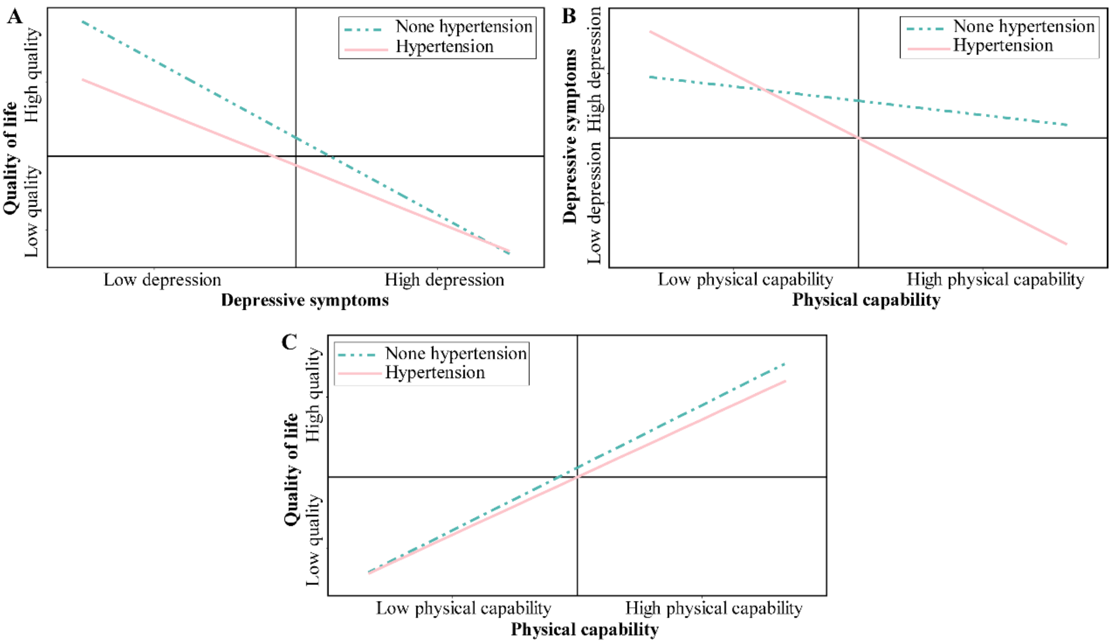

Fig 3. Interactive effect of hypertension on the relationship between physical capability, QoL, and depressive symptoms. This is consistent with the assumptions of Theoretical Model 3. Hypertension acts as a moderating variable between Physical capability and QoL.

Discussion

This study primarily explored a multiple mediation model of the ‘physical capability to QoL via BSE and depressive symptoms’ pathway in a group of stroke patients from China based on the Wilson and Cleary model. Our findings are consistent with a previous study, showing the significant mediation effect of BSE on the relationship between physical capability and QoL [20]. As hypothesized, the physical capability showed an indirect positive correlation with QoL through BSE; a higher level of physical capability indicated higher BSE, thereby improving the patient’s QoL.

However, we found no mediation effect of depressive symptoms on the relationship between physical capability and QoL. According to French et al. [19], depressive symptoms moderate the relationships between physical capability, self-efficacy, and participation in stroke patients. Although the hypothesis about depressive symptoms mediating the relationship between physical capability and QoL was not supported, all the paths in our model were significant. The present findings suggest that a high level of physical capability can predict a lower severity of depressive symptoms and a better QoL, while a high severity of depressive symptoms predicts a lower QoL. This supports our hypothesis.

Our results also demonstrated that hypertension moderated the relationship between physical capability, depressive symptoms, and QoL in stroke patients. The patients with hypertension had decreased physical capability, which in turn increased depressive symptoms. This result is consistent with Sok et al. showing a strong association between depressive symptoms and hypertension [35]. Accordingly, another study reported that depressed patients with hypertension had poor control of blood pressure [53]. The possibility exists that antidepressant therapy interferes with blood pressure control in ~~hypertension patients~~, but further research on this topic is warranted. Additionally, a recent study showed that increased walking (physical) activity lowers hypertension risk in postmenopausal women [54]. Our study also indicated that physical activity positively influences hypertension risk, even in a mixed-gender population. Conversely,

patients with high blood pressure were found to be less active than those without hypertension. Furthermore, QoL negatively correlates with stroke risk factors such as hypertension [55, 56]. In general, hypertension is an important moderator of relationships between physical capability, depressive symptoms, and QoL.

However, there is a lack of literature examining the relationship between hypertension and BSE, which must be explored in both a qualitative and quantitative manner. Similarly, we found no evidence that hypertension moderated the 'physical capability to QoL via BSE' pathway. Although our findings did not show that diabetes and gender moderated the relationship between physical capability and QoL, this may be attributable to the relatively small number of individuals with diabetes and females in this cohort. Therefore, further research is needed with a larger and more diverse sample.

The theoretical model in this study was built based on a previous model suggesting that BSE is a significant mediator and hypertension has a moderating effect on the 'physical capability to QoL through depressive symptoms' pathway. The mediating effect of BSE and the moderating effect of hypertension may help interpret the mechanisms of the relationship between physical capability and QoL. Our conceptual model facilitates the understanding of these associations to make targeted strategies promoting QoL and rehabilitation. Patients show improved balance function, which naturally enhances balance confidence, leading to a significant improvement in QoL [18]. Health professionals should incorporate interventions to enhance balance confidence and the control of blood pressure in their management of physical and mental rehabilitation.

Limitations

There are some limitations to our research. First, we did not consider the complex nature of the QoL index, which may complicate relationships among the various missing data of clinical variables. Researchers should implement rigorous strategies to minimize missing data and place greater emphasis on exploring the intricacies of the QoL index in more detail, taking into account its multiple dimensions. Second, we did

not take into account the impact of stroke subtypes, a possible confounding parameter in this study. Future research should consider stratifying stroke patients by subtype, which would facilitate a more precise analysis of how different stroke types may influence the outcomes. Third, the cross-sectional study design restricts the interpretation and generalization of our results. An additional longitudinal study may help verify the conclusions of this study.

Conclusion

This study provided a better insight into the relationship between physical capability and QoL via BSE in stroke patients, which may help establish appropriate treatment for these individuals. Our results uncovered the underlying moderating effect of hypertension on relationships between physical capability, depressive symptoms, and QoL. An effective rehabilitation strategy can achieve concrete targets for stroke risk patients via BSE, improving their QoL [57].

Acknowledgment

The authors would like to thank all the participants in the study and MJEditor (www.mjeditor.com) for their linguistic assistance during the preparation of this manuscript.

Author Contributions

Conceptualization: Xue Yang.

Formal analysis: Qian Liu.

Investigation: Yihuan Lu.

Methodology: Xue Yang.

Supervision: Liqing Yao.

Validation: Xue Yang, Hongmei Zhang, Qian Liu.

Writing – original draft: Xue Yang.

Writing – review & editing: Xue Yang, Hongmei Zhang.

Consent to participate

Written informed consent was obtained from all patients before participation.

Reference

1. Global, regional, and national burden of stroke, 1990-2016: a systematic analysis for the Global Burden of Disease Study 2016 Lancet Neurol, 2019. 18 (5): p.439-58. https://10.1016/s1474-4422(19)30034-1 PMID: 30871944

2. Chen Z, Jiang B, Ru X, Sun H, Sun D, Liu X, et al., Mortality of Stroke and Its Subtypes in China: Results from a Nationwide Population-Based Survey Neuroepidemiology, 2017. 48 (3-4): p.95-102. https://10.1159/000477494 PMID: 28586776

3. Kim A, Cahill E, Cheng N. Global Stroke Belt: Geographic Variation in Stroke Burden Worldwide Stroke, 2015. 46 (12): p.3564-70. https://10.1161/STROKEAHA.115.008226 PMID: 26486867

4. Jurgens C, Lee C, Aycock D, Masterson Creber R, Denfeld Q, DeVon H, et al., State of the Science: The Relevance of Symptoms in Cardiovascular Disease and Research: A Scientific Statement From the American Heart Association Circulation, 2022. 146 (12): p.e173-e84. https://10.1161/CIR.0000000000001089 PMID: 35979825

5. Labeit B, Michou E, Hamdy S, Trapl-Grundschober M, Suntrup-Krueger S, Muhle P, et al., The assessment of dysphagia after stroke: state of the art and future directions Lancet Neurol, 2023. 22 (9): p.858-70. https://10.1016/S1474-4422(23)00153-9 PMID: 37596008

6. Braun L, Grady K, Kutner J, Adler E, Berlinger N, Boss R, et al., Palliative Care and Cardiovascular Disease and Stroke: A Policy Statement From the American Heart Association/American Stroke Association Circulation, 2016. 134 (11): p.e198-e225. https://10.1161/CIR.0000000000000438 PMID: 27503067

7. Turana Y, Tengkawan J, Chia Y, Nathaniel M, Wang J, Sukonthasarn A, et al., Hypertension and stroke in Asia: A comprehensive review from HOPE Asia J Clin Hypertens (Greenwich), 2021. 23 (3): p.513-21. https://10.1111/jch.14099 PMID: 33190399

8. Nor A M N, Noor A A A, Saperi S, Syed M A. Functional limitation and health related quality of life and associated factors among long term stroke survivors in a

Malaysian community Med J Malaysia, 2016. 71 (6): p.313-21.

9. Haghgoo H, Pazuki E, Hosseini A, Rassafiani M. Depression, activities of daily living and quality of life in patients with stroke J Neurol Sci, 2013. 328 (1-2): p.87-91. https://10.1016/j.jns.2013.02.027 PMID: 23522526

10. Muren M, Hutler M, Hooper J. Functional capacity and health-related quality of life in individuals post stroke Top Stroke Rehabil, 2008. 15 (1): p.51-8. https://10.1310/tsr1501-51 PMID: 18250074

11. Franco F, Fay H, Marco G, Antonio N, Andrea G. Using psychometric techniques to improve the Balance Evaluation Systems Test: the mini-BESTest J Rehabil Med, 2010. 42 (4): p.323-31. https://10.2340/16501977-0537 PMID: 20461334

12. Naghdi S, Ansari N, Mansouri K, Hasson S. A neurophysiological and clinical study of Brunnstrom recovery stages in the upper limb following stroke Brain Inj, 2010. 24 (11): p.1372-8. https://10.3109/02699052.2010.506860 PMID: 20715900

13. Xue Y, Yutong F, Hong F, Jibing O, Jimin C, Liqing Y. Exploring the complex pathways between somatosensory impairment, physical ability and affective symptoms following stroke: mediation analyses Annals of Palliative Medicine, 2022. 11 p.2395-409.

14. Di Monaco M, Trucco M, Di Monaco R, Tappero R, Cavanna A. The relationship between initial trunk control or postural balance and inpatient rehabilitation outcome after stroke: a prospective comparative study Clin Rehabil, 2010. 24 (6): p.543-54. https://10.1177/0269215509353265 PMID: 20511303

15. Arlene A S, Marieke Van P, Peter A A, Kristine K M, Stephanie A C, Stephen J P. Balance is associated with quality of life in chronic stroke Top Stroke Rehabil, 2013. 20 p.340-6.

16. Schmid A, Van Puymbroeck M, Knies K, Spangler-Morris C, Watts K, Damush T, et al., Fear of falling among people who have sustained a stroke: a 6-month longitudinal pilot study The American journal of occupational therapy : official publication of the American Occupational Therapy Association, 2011. 65 (2): p.125-32. https://10.5014/ajot.2011.000737 PMID: 21476359

17. Arlene A S, Sarah E A, Valerie A J, M Jane R, Stephanie A S, Marieke Van P. Fear of falling in people with chronic stroke The American journal of occupational therapy : official publication of the American Occupational Therapy Association, 2015. 69 (3): p.6903350020. https://10.5014/ajot.2015.016253 PMID: 25871606

18. Caryne T, Krista L B, William C M, Janice J E. Balance Confidence: A Predictor of Perceived Physical Function, Perceived Mobility, and Perceived Recovery 1 Year After Inpatient Stroke Rehabilitation Arch Phys Med Rehabil, 2016. 97 (7): p.1064-71. https://10.1016/j.apmr.2016.03.004 PMID: 27060032

19. Margaret A F, Allison M, Ryan T P, Darcy S R. Depressive Symptoms Moderate the Relationship Among Physical Capacity, Balance Self-Efficacy, and Participation in People After Stroke Phys Ther, 2021. 101 (12): p.1-10. https://10.1093/ptj/pzab224 PMID: 34636909

20. Margaret A F, Meghan F M, Ryan P, Darcy R. Self-efficacy Mediates the Relationship between Balance/Walking Performance, Activity, and Participation after Stroke Top Stroke Rehabil, 2016. 23 (2): p.77-83.

https://10.1080/10749357.2015.1110306 PMID: 26653764

21. Yannis Yan L, Lisha W, Ying Y, Yangkun C, Vincent C T M, Gabor S U, et al., Association Between Behavioral Dysexecutive Syndrome and the Health-Related Quality of Life Among Stroke Survivors Front Psychiatry, 2020. 11 p.563930. https://10.3389/fpsyt.2020.563930 PMID: 33101083

22. Hellstrom K, Lindmark B, Wahlberg B, Fugl-Meyer AR. Self-efficacy in relation to impairments and activities of daily living disability in elderly patients with stroke: a prospective investigation J Rehabil Med, 2003. 35 (5): p.202-7. https://10.1080/16501970310000836 PMID: 14582550

23. Szczepanska-Gieracha J, Mazurek J. The Role of Self-Efficacy in the Recovery Process of Stroke Survivors Psychol Res Behav Manag, 2020. 13 p.897-906. https://10.2147/PRBM.S273009 PMID: 33177896

24. Koleck M, Gana K, Lucot C, Darrigrand B, Mazaux JM, Glize B. Quality of life in aphasic patients 1 year after a first stroke Qual Life Res, 2017. 26 (1): p.45-54. https://10.1007/s11136-016-1361-z PMID: 27405871

25. Wei-Quan L, Ting-Yuan H, Lan L, Yun-Ou Y, Yao-Hui L, Min-Ying S, et al., Prevalence and related factors of depression and falls among the elderly living in rural communities of Guangzhou Psychology, health & medicine, 2020. 25 (8): p.980-8. https://10.1080/13548506.2020.1714064 PMID: 31967487

26. Lin C, Babiker A, Srdanovic N, Kocherginsky M, Harvey RL. Depressive symptoms after stroke are associated with worse recovery International journal of psychiatry in medicine, 2020. 55 (4): p.227-38. https://10.1177/0091217420905459 PMID: 32050815

27. León LPd, Mangin JPL, Ballesteros S. Psychosocial Determinants of Quality of Life and Active Aging. A Structural Equation Model International Journal of Environmental Research and Public Health, 2020. 17 (17): https://10.3390/ijerph17176023

28. R G Robinson, L B Starr, K L Kubos, T R Price. A Two-Year Longitudinal Study of Post-Stroke Mood Disorders: Findings During the Initial Evaluation Stroke, 1983. 14 p.736-41.

29. Heiland EG, Welmer AK, Wang R, Santoni G, Fratiglioni L, Qiu C. Cardiovascular Risk Factors and the Risk of Disability in Older Adults: Variation by Age and Functional Status J Am Med Dir Assoc, 2019. 20 (2): p.208-12 e3. https://10.1016/j.jamda.2018.05.013 PMID: 30006016

30. Tusa BS, Geremew BM, Tefera MA. Heath related quality of life and associated factors among adults with and without diabetes in Adama city East Shewa, Ethiopia 2019; using generalized structural equation modeling Health Qual Life Outcomes, 2020. 18 (1): p.83. https://10.1186/s12955-020-01337-9 PMID: 32228598

31. Li ZH, Lv YB, Kraus VB, Yin ZX, Liu SM, Zhang XC, et al., Trends in the Incidence of Activities of Daily Living Disability Among Chinese Older Adults From 2002 to 2014 J Gerontol A Biol Sci Med Sci, 2020. 75 (11): p.2113-8. https://10.1093/gerona/glz221 PMID: 31603986

32. Qian J, Ren X. Association between comorbid conditions and BADL/IADL disability in hypertension patients over age 45: Based on the China health and

retirement longitudinal study (CHARLS) Medicine (Baltimore), 2016. 95 (31): p.e4536. https://10.1097/MD.0000000000004536 PMID: 27495110

33. Katan M, Luft A. Global Burden of Stroke Seminars in neurology, 2018. 38 (2): p.208-11. https://10.1055/s-0038-1649503 PMID: 29791947

34. Zhang XP, Pan JH, Wan LH, Liu ZY, Mo MM, Wang MY, et al., Factors influencing health behaviour, blood pressure control, and disability in hypertensive ischaemic stroke patients after a comprehensive reminder intervention J Adv Nurs, 2020. 76 (6): p.1384-93. https://10.1111/jan.14340 PMID: 32128865

35. Sok SR, Yim E, Sim H, Sim HJ. Relationships among Self-Care Competency, Presence of Depressive Symptom, and Health-Related Quality of Life of Korean Stroke Patients Clin Nurs Res, 2021. 30 (5): p.670-9. https://10.1177/1054773820913984 PMID: 32207330

36. Kristopher J P, Derek D R, Andrew F H. Addressing moderated mediation hypotheses. Theory, methods, and prescriptions Multivariate Behav Res, 2007. 42 p.185-227.

37. Ira B. Wilson, Paul D. Cleary. Linking Clinical Variables With Health-Related Quality of Life JAMA, 1995. 273 p.59-65.

38. Saengsiri AO, Hacker ED. Conducting quality of life research in people with coronary artery disease in non-English-speaking countries: conceptual and operationalization issues J Cardiovasc Nurs, 2015. 30 (1): p.74-84. https://10.1097/JCN.0000000000000116 PMID: 24365869

39. Association WM. World Medical Association Declaration of Helsinki Ethical Principles for Medical Research Involving Human Subjects JAMA, 2013. 310 p.2191-4.

40. Sawner K JL. Brunnstrom movement therapy in hemiplegia: a neurophysilogical approach. 2nd ed. Philadelphia: JB Lippincott; 1992.

41. Huang CY, Lin GH, Huang YJ, Song CY, Lee YC, How MJ, et al., Improving the utility of the Brunnstrom recovery stages in patients with stroke: Validation and quantification Medicine (Baltimore), 2016. 95 (31): p.e4508. https://10.1097/MD.0000000000004508 PMID: 27495103

42. Tsang CS, Liao LR, Chung RC, Pang MY. Psychometric properties of the Mini-Balance Evaluation Systems Test (Mini-BESTest) in community-dwelling individuals with chronic stroke Phys Ther, 2013. 93 (8): p.1102-15. https://10.2522/ptj.20120454 PMID: 23559522

43. Florence I. Mahoney DWB. Functional evaluation: the Barthel index Maryland State Medical Journal, 1965. 14 p.56-61.

44. Collin C, Wade D T, Davies S, Horne V. The Barthel ADL Index: a reliability study Int Disabil Srudies, 1988. 10 p.61-3.

45. Powell L E, Myers A M. The Activities-specific Balance Confidence (ABC) Scale Journal of Gerontology, 1995. 50A p.M28-M34.

46. Zhang D, Tian F, Gao W, Huang Y, Huang H, Tan L. The Chinese Short Version of the Activities-Specific Balance Confidence Scale: Its Validity, Reliability, and Predictive Value for Future Falls in Community-Dwelling Older Adults Clin Interv Aging, 2022. 17 p.1483-91. https://10.2147/CIA.S380921 PMID: 36212511

47. Fava G, Kellner R, Munari F, Pavan L. The Hamilton Depression Rating Scale in normals and depressives Acta psychiatrica Scandinavica, 1982. 66 (1): p.26-32. https://10.1111/j.1600-0447.1982.tb00911.x PMID: 7124430
48. Matthias J. Mu¨ller AD. Standardized rater training for the Hamilton Depression Rating Scale (HAMD-17) in psychiatric novices J Affect Disord, 2003. 77 p.65 – 9. https://10.1016/S0165-0327(02)00097-6
49. Linda K. Muthen BOM. Mplus User's Guide. Los Angeles: CA: Muth´en & Muth´en; 2012.
50. Yiran D, Chao-Ying J P. Principled missing data methods for researchers SpringerPlus, 2013. 2 p.222.
51. Daire Hooper J C, Michael R M. Structural Equation Modelling Guidelines for Determining Model Fit Journal of Business Research Methods, 2008. 6 p.53-60.
52. Kline R B. Principles and Practice of Structual Equation Modeling, 4th ed 4th ed. New York: Guilford Press; 2016.
53. Scalco A Z, Mônica Z S, João B S A, Francisco L N. hypertension and depression CLINICS, 2005. 60 p.241-50.
54. Miller C R, Wactawski-Wende J, Manson J E, Haring B, Hovey K M, Laddu D, et al., Walking Volume and Speed Are Inversely Associated With Incidence of Treated Hypertension in Postmenopausal Women Hypertension, 2020. 76 (5): p.1435-43. https://10.1161/HYPERTENSIONAHA.120.15839 PMID: 32981366
55. Yao H, Zhang J, Wang Y, Wang Q, Zhao F, Zhang P. Stroke risk and its association with quality of life: a cross-sectional study among Chinese urban adults Health Qual Life Outcomes, 2021. 19 (1): p.236. https://10.1186/s12955-021-01868-9 PMID: 34627278
56. Alotaibi S M, Alotaibi H M, Alolyani A M, Abu Dali F A, Alshammari A K, Alhwiesh A A, et al., Assessment of the stroke-specific quality-of-life scale in KFHU, Khobar: A prospective cross-sectional study Neurosciences (Riyadh), 2021. 26 (2): p.171-8. https://10.17712/nsj.2021.2.20200126 PMID: 33814370
57. Silva S M, Correa F I, Faria C D, Pereira G S, Attie E A, Correa J C. Reproducibility of the items on the Stroke Specific Quality of Life questionnaire that evaluate the participation component of the International Classification of Functioning, Disability and Health Disabil Rehabil, 2016. 38 (24): p.2413-8. https://10.3109/09638288.2015.1130178 PMID: 26879175

Figure

Click here to access/download;Figure;Fig2.tif

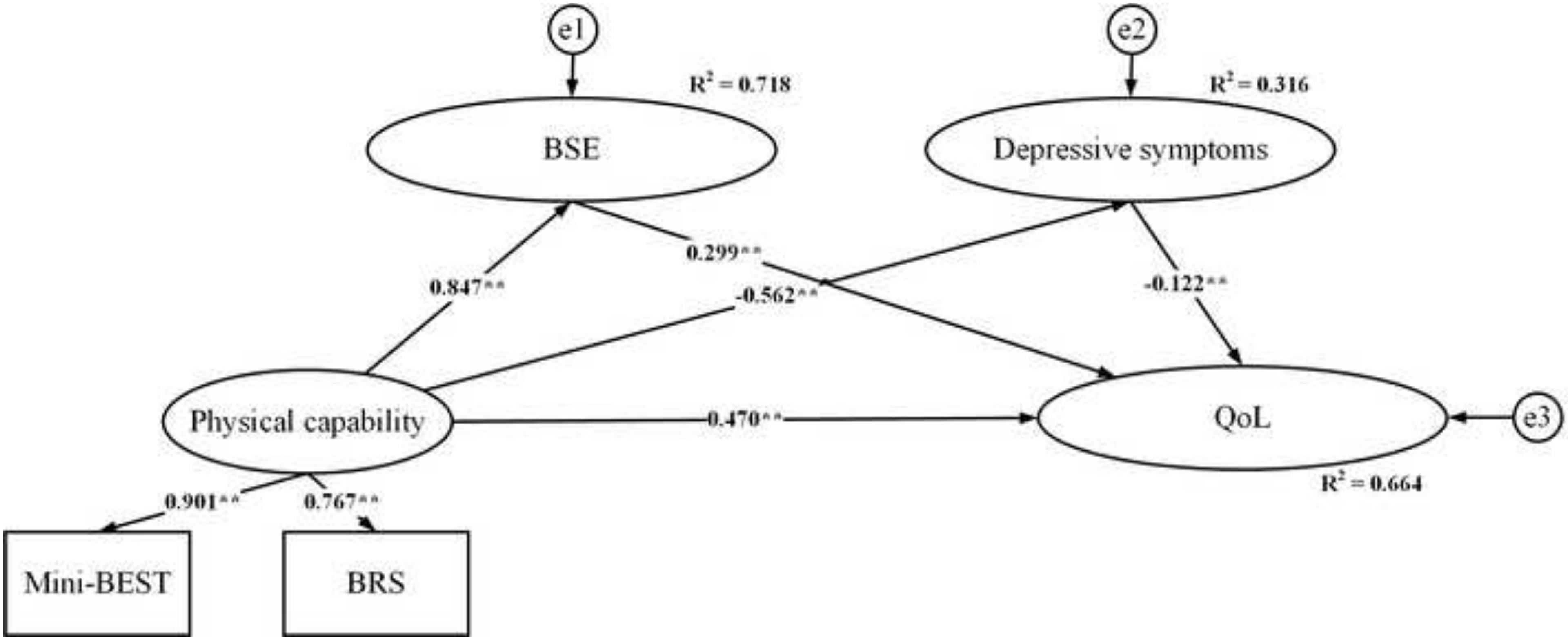

Figure

Click here to access/download;Figure;Fig1.tif

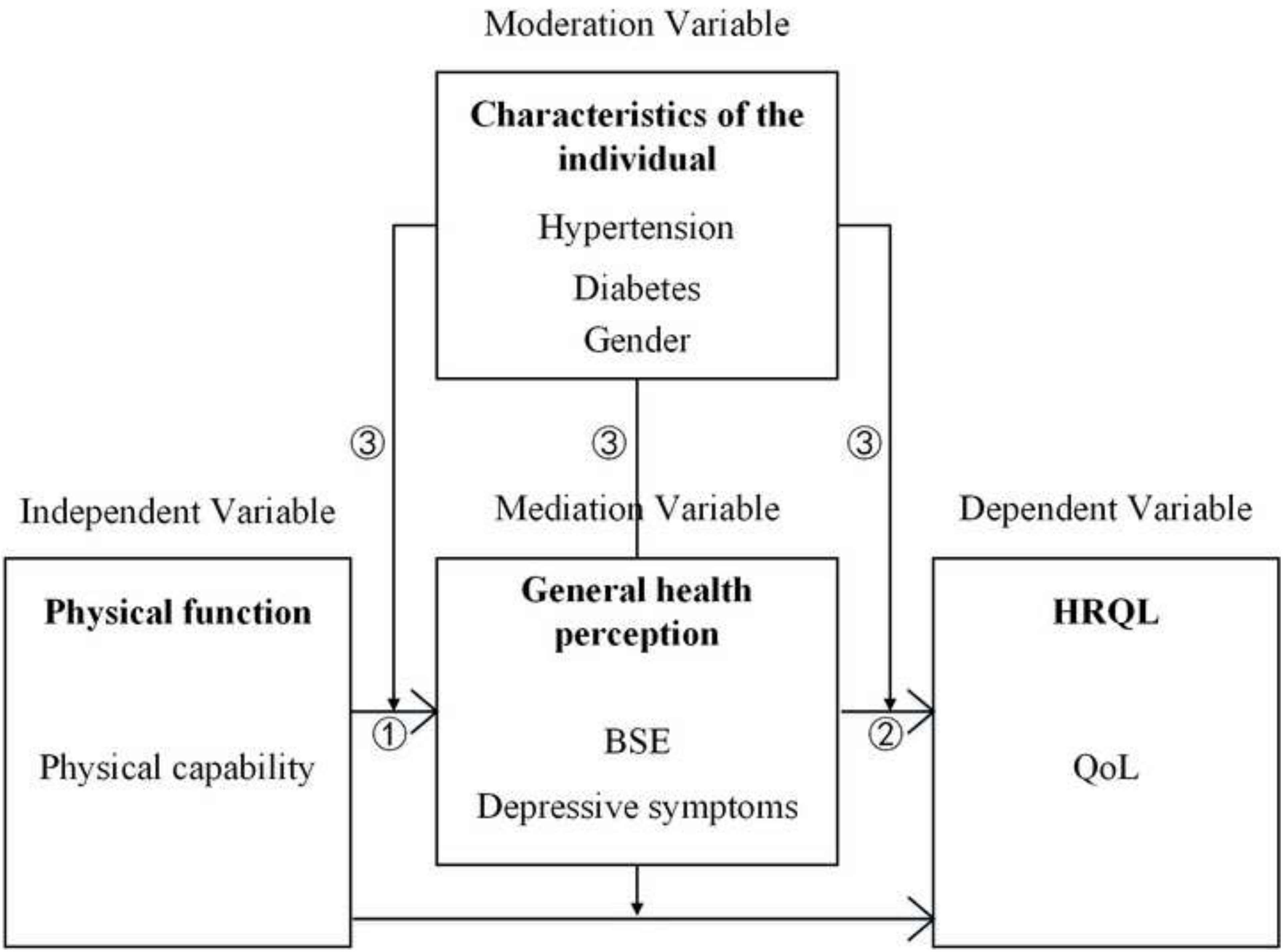

Click here to access/download;Figure;Fig3.tif

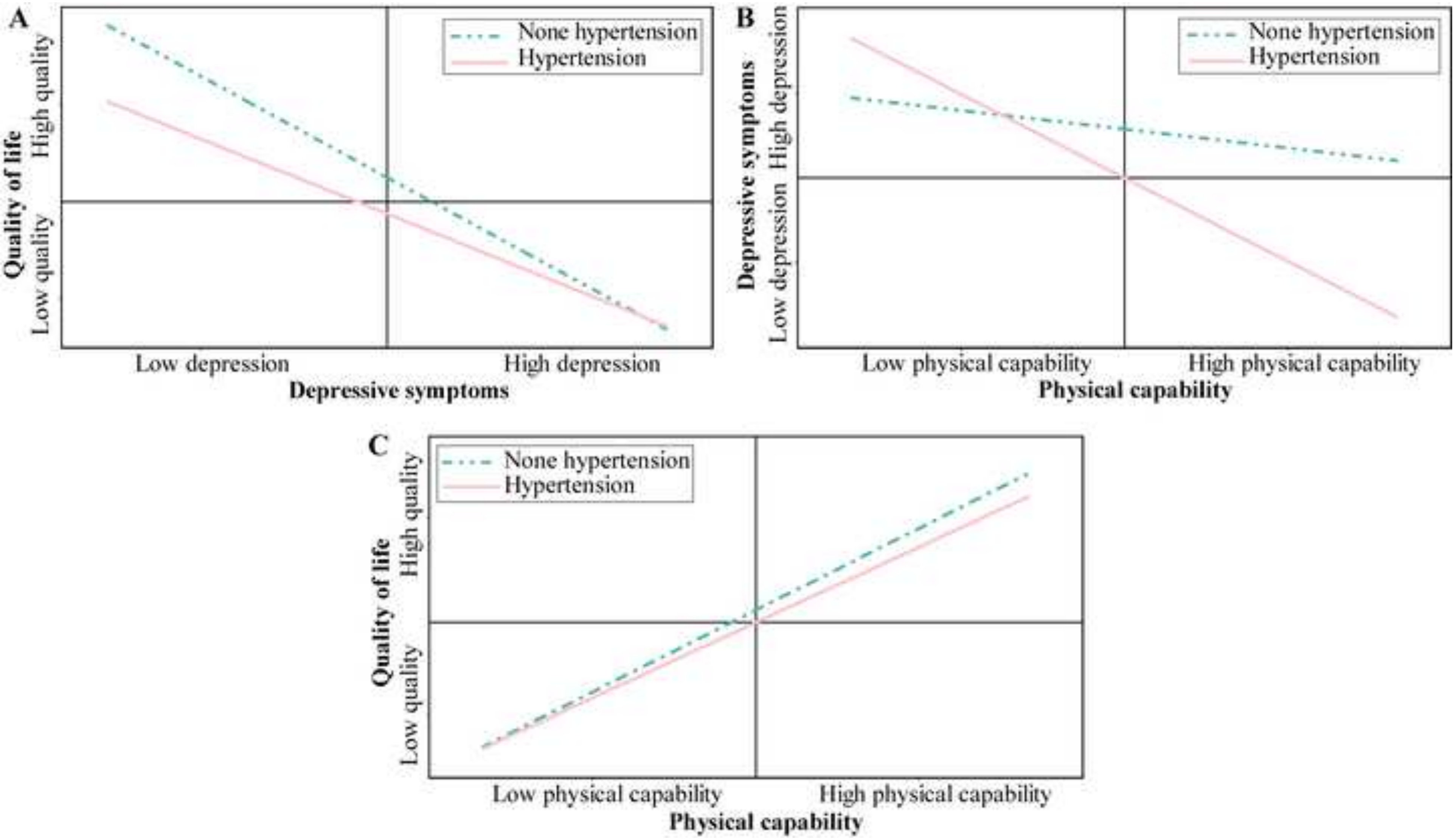

Click here to access/download
Supporting Information
Mplus_moderation.xlsx

Click here to access/download
Supporting Information
data - revision.xlsx

Mediation and moderation analysis of the association between physical capability and quality of life among stroke patients

Xue Yang [1¶], Hongmei Zhang [1¶], Yihuan Lu [1], Qian Liu [1], Liqing Yao [1*]

1 Department of Rehabilitation Medicine, the Second Affiliated Hospital of Kunming Medical University, Kunming, Yunnan, China

* Corresponding author

E-mail: yaoliqing98731@163.com(LY)

¶These authors contributed equally to this work.

Abstract

To estimate the extent to which physical capability, depressive symptoms, balanced self-efficacy (BSE), and other risk factors, that are interrelated with stroke, influence the quality of life (QoL) in stroke patients. A theoretical model based on Wilson and Cleary's model tested the specific hypotheses: 1) physical capability has an indirect effect on QoL mediated by BSE; 2) physical capability has an indirect effect on QoL by depressive symptoms; 3) stroke risk factors (hypertension/diabetes/gender) moderate the above relationship. Six hundred and seventy stroke patients were enrolled from ten different hospitals in Yunnan province from 2019 to 2021. Patients' mental and physical function was assessed using the Brunnstrom recovery stage (BRS), mini-balance evaluation system test (Mini-BEST), Barthel index (BI), Activities-specific Balance Confidence scale (ABC), and Hamilton depression scale (HAM-D). The structural equation model (SEM) was used to test the moderated mediation model in Mplus 8.0 software. The model showed a good fit (RMSEA = 0.075, SRMR = 0.010). BSE significantly mediated the relationship between physical capability and QoL (β = 0.322, p = 0.002). Hypertension was found a significant moderator of all the direct paths from physical capability to QoL through depressive symptoms (**B** = 0.412, p = 0.015; **B** = 0.831, p = 0.020, respectively). This study provides a better insight into the relationship between physical capability and QoL via BSE in stroke patients, which may help establish appropriate treatment for these individuals.

Introduction

Stroke is a primary cause of long-term neurological disability, with more than 80 million stroke survivors globally in 2016 [1]. Compared to Europe, America, and Australia, Asia has the largest stroke burden, especially in China due to its large and aging population [2, 3]. Stroke decreases the survivors' quality of life (QoL) because it

can cause physical and cognitive impairments, emotional distress, and fatigue[4, 5]. Additionally, it can also lead to social isolation and a loss of independence[6]. Therefore, it is a serious health problem worldwide, especially in Asia [7]. The reduction in QoL often accompanies the increased risk of lifestyle diseases, including hypertension, diabetes, and an array of physical dysfunctions. Thus, improving QoL can improve the overall health and physical ability of stroke survivors. Various factors, including physical [8] and psysical [9], affect the QoL of stroke survivors, which must be taken into account by rehabilitation professionals to improve the QoL.

A recent study showing a strong relationship between physical capability and QoL in stroke patients has raised serious concerns [10]. Physical capability denotes one’s capacity to completely undertake a task. Normally, it is evaluated based on the assessment of an individual’s balance and motor functions, such as the MiniBest scale [11], and Brunnstrom stages of stroke recovery [12]. Earlier research has shown that better physical capability is an important predictor of stroke survivors’ QoL [8]. Additionally, physical capability is associated with balance function [13], which may also pose risks to patients’ QoL [14, 15].

Balance impairment could lead stroke patients to develop anxiety-related or depression-related distress behaviors such as fear of falling [16, 17]. The fear of falling stroke survivors frequently leads to activity and participation limitations as a consequence of poststroke physical disability [17]. Therefore, enhancing the balance confidence may improve patients’ QoL [18]. Balance confidence can be assessed using the “Activities-specific Balance Confidence Scale (ABC)” questionnaire [19]. It is also called Balance self-efficacy (BSE), which means an individual’s belief in his or her ability to accomplish a task [20]. Studies in the last few decades have produced fairly convergent results, indicating that higher BSE is linked to better QoL of stroke patients [21-23]. French et al. proposed a theoretical model to explain the mediating role of BSE on the relationship between the patient's physical capability and participation [20]. Using the above theoretical model, we propose the first hypothesis, considering the mediation effect of BSE on the relationship between physical capability and QoL.

Additionally, poor QoL has been found strongly associated with severe depressive symptoms [24, 25]. Evidence suggests that depressive symptoms after stroke harm motor outcomes [26]. One research study that there is a negative relationship between depression and the perception of QoL [27]. Conversely, physical impairments contribute to post-stroke depressive disorders [28]. Thus, we propose the second hypothesis that the relationship between physical capabilities and QoL may also be mediated by depressive symptoms.

Besides, other risk factors such as hypertension, diabetes, and gender may further impact these relationships among physical capability, depressive symptoms, BSE, and QoL [29, 30]. Hypertension is a leading risk factor for stroke in Asia [3, 7], significantly increasing the risk of ADL disability [31]. Hypertension with other comorbid conditions lowers the patient's QoL and physical capability [32, 33]. Interestingly, one study demonstrated that enhancing the self-efficacy of stroke patients can help them improve blood pressure [34]. Concerning diabetes, ischemic stroke is a well-known macrovascular complication of diabetes. A study reported that diabetic stroke patients have poorer outcomes than non-diabetic stroke patients [35]. Notably, one study indicated gender association with health-related QoL [35]. Therefore, this study also examined the effect of these moderators (hypertension, diabetes, gender) on the relationships among physical capability, BSE, depressive symptoms, and QoL. Accordingly, here, we propose the third hypothesis that hypertension/diabetes/gender may act as moderators affecting the mediated relationship in stroke patients [36].

To fully elucidate the complex relationships among factors that contribute to QoL in stroke, Wilson and Cleary's 1995 model (W-C model) is applied to link physical function (physical capability), general health perception (BSE, depressive symptoms), and QoL [37]. General health perception refers to self-reported health assessment [38]. A hypothetic and theoretic model of the expected moderated mediation is described in Fig 1. The model tests specific hypotheses: (1) physical capability has an indirect effect on QoL mediated by BSE; (2) physical capability has an indirect effect on QoL by depressive symptoms; (3) stroke risk factors (hypertension/diabetes/gender) moderate

the above relationship.

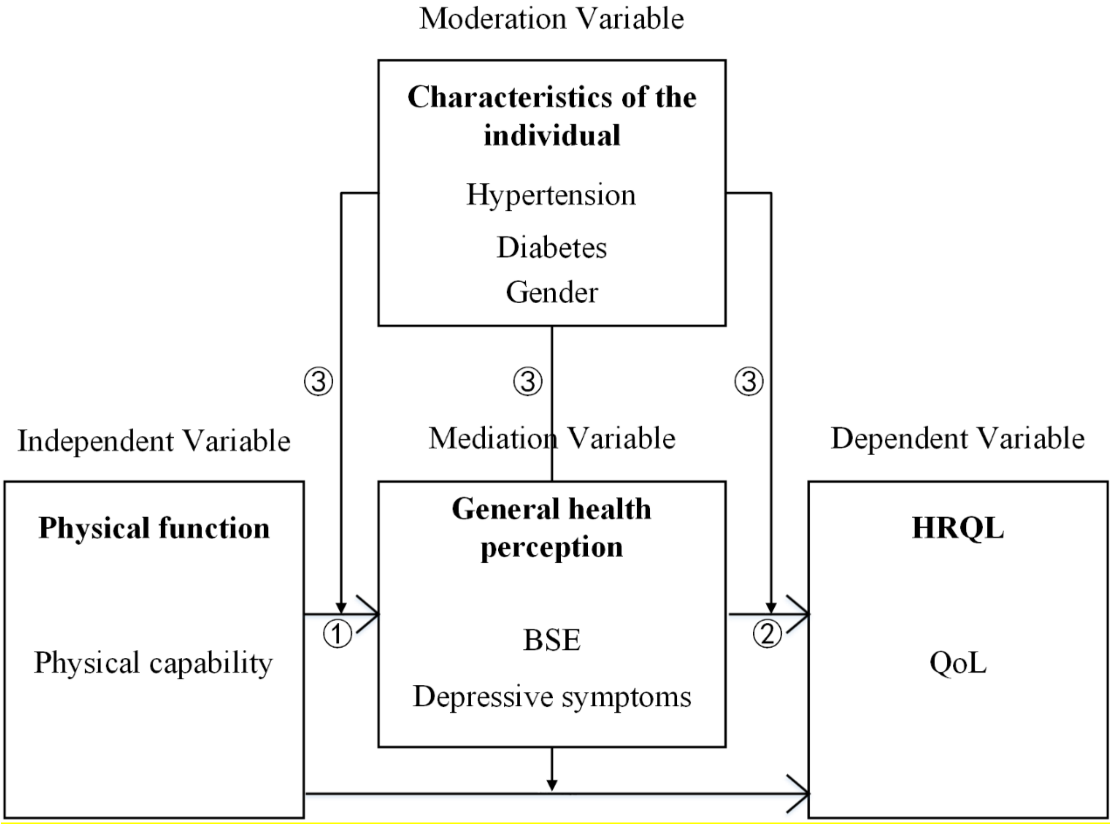

Fig 1. The theoretical model is based on Wilson and Cleary's model. ①: Hypothesis 1 of the current study; ②: Hypothesis 2 of the current study; ③: Hypothesis 3 of the current study. BSE: Balance Self-Efficacy; HRQL: Health-Related Quality of Life; QoL: Quality of Life. The arrows represent the hypothesized directional relationships between the variables in the model.

Methods

Study design

The research was a multicenter cross-sectional study conducted from April 2019 to April 2020. In total, 1,765 participants were recruited using the consecutive sampling method, consisting of inpatients from ten different rehabilitation institutions. Ethical approval was obtained from the local ethics committees (approval No. PJ-2019-04) and the research was performed according to the Declaration of Helsinki [39]. Written

informed consent was obtained from all patients before participation.

Participants

Patients' participation criteria were as follows: (I) age, >18 years; (II) diagnosis of hemorrhagic or ischemic stroke for > 1 month; (III) stable vital signs, and (IV) signed informed consent. The participant exclusion criteria were as follows: (I) presence of any mental diagnosis before stroke according to the Diagnostic and Statistical Manual of Mental Disorders classification (i.e., major depressive disorder, bi-directional affective disorder, and obsessive-compulsive disorder, etc.); (II) malignant tumors, and/or; (III) dementia, loss of consciousness, or severe cardiopulmonary, liver, or kidney dysfunctions; (IV) pre-existing physical disability from any cause, such as fracture, osteoarthritis, etc. Out of the 1,765 participants, 245 did not meet the inclusion criteria, and 850 were excluded based on exclusion criteria. Finally, 670 participants were included in the structural equation model analysis.

Procedure

The case data used in this cross-sectional study were obtained from stroke patients across ten rehabilitation facilities in Yunnan Province, China. Initially, ten hospitals were invited to participate in a training program conducted every three months. Subsequently, two trained researchers visited various hospitals periodically to collect data and perform quality control [13]. After patients provided informed consent, data collectors conducted a standardized assessment, which included a neurological evaluation covering demographic information, clinical data, and both neurological and neuropsychological functional assessments. Assessment outcomes and patient information were entered into the electronic database of our hospital department (Department of Rehabilitation, the Second Affiliated Hospitals of Kunming Medical University).

Measurement

Physical capability: The Brunnstrom recovery stage (BRS) [40] is conducted to

evaluate post-stroke motor recovery in the affected limbs [12]. The evaluation consists of six levels, Stage 1 (flaccid paresis) to Stage 6 (approximately normal paresis). The total score is the summation of all scores of the limbs. A high score suggests a positive recovery. The Rasch reliability coefficient was above 0.90 [41]. Mini-BEST, a mini-balance evaluation system test [11], assesses the dynamic balance of the patient. The mini-BEST had high internal consistency and interrater reliability [42]. It includes 14 items, each rated from 0 (severe) to 2 (normal). The maximum score is 28 points. A higher score indicates better balance and postural control.

Quality of life (QoL): The Barthel index (BI) [43], a functionally independent tool, evaluates the patient's performance in daily life activities, with a total score ranging from 0 to 100. A high score indicates a better ability to live independently. BI demonstrates good validity and reliability [44].

Balance self-efficacy (BSE): ABC, an Activities-specific Balance Confidence scale, evaluates individuals' balance confidence while carrying out specific activities [45]. ABC shows excellent internal consistency and test-retest reliability [46]. This 11-point scale ranges from 0 (no confidence) to 100 (complete confidence). The total score denotes the average score.

Depressive symptoms: The Hamilton depression scale (HAM-D) is widely used to assess the severity of depression [47]. HAM-D demonstrates satisfactory reliability [48]. It included 17 items that were scored on a scale of 0 to 4. A higher score suggests a higher severity of depression.

Statistics analysis

Descriptive statistics and bivariate correlation analysis were conducted with IBM SPSS 22.0. Normal data distribution was confirmed from the normal probability plots. Continuous data, with normal or near normal distribution, are expressed as mean ± SD; median and inter-quartile ranges [P_{25}-P_{75}] are reported in case of nonnormal data distribution. Categorical data are expressed as rates and percentages. Pearson or Spearman correlation analyses were conducted to examine the correlations among BRS, Mini-BEST, QoL, BSE, and depressive symptoms.

The Mplus 8.0 program [49] was used to employ structural equation modeling (SEM) to test the hypothesized model. Some variables (<5%) had missing data. Missing data

imputation uses a process of multiple imputations assuming data missing at random [50]. The maximum likelihood estimation method was applied to assess the structural equation model. The goodness-of-fit indices are widely used for structural equation models [51]. χ^2/df ≤ 5 (the ratio of χ^2 to the degree of freedom) indicates an acceptable fitting of the data. The comparative five indexes (CFI) and the Tucker-Lewis index (TLI) of > 0.95 represent an excellent model fit. The SRMR (standardized root mean square residual) and RMSEA (root mean square error of approximation) indices of ≤ 0.08 indicate an acceptable fit of the model. $p < 0.05$ denotes the statistical significance of the data.

Additionally, a recommended bias-corrected bootstrapped method was employed to examine the mediators of BSE and depressive symptoms on the relationship between physical capability and QoL and generate 95% bias-corrected confidence intervals (CIs) [52], with the procedure of 5,000 samples. A multi-group comparison test was employed to examine the indirect effects of physical capability and QoL differed between BSE and depressive symptoms. Also, the possible moderating effects of hypertension, diabetes, and gender among simple paths in the multiple-mediation model were examined. A statistically significant difference in the path coefficients between subgroups indicates the moderating effect of the path [36].

Results

Demographic characteristics

Six hundred and seventy participants (438 males, 232 females, mean age 61.4 ± 11.9 years) were involved in this study. The majority of participants (556, 83.0%) had an ischemic stroke, while hemorrhagic- and other strokes were reported in 101 (15.1%) and 13 (1.9%) patients respectively. Of the total number, 292 (43.6%) had a left-sided stroke (right hemiparesis), and 297 (44.3%) had a right-sided stroke (left hemiparesis). Patients with hypertension accounted for 68.5% (459), while patients with diabetes accounted for 11.3% (76).

Correlations

The median score of the ABC was 88.13, whereas the mean assessment scales of mini-BEST, BRS, BI, and HAM-D are outlined in Table 1. We found significant correlations between BRS, Mini-BEST, QoL, BSE, and depressive symptoms ($p < 0.001$) (Table 2). BRS showed a positive correlation with Mini-BEST (r = 0.696), QoL (r = 0.633), and BSE (r = 0.644), but was negatively correlated with depressive symptoms (r = -0.438). Additionally, Mini-BEST showed a significantly positive correlation with BSE, and QoL (r =0.789, r = 0.701), but a negative correlation with depressive symptoms (r = -0.492). BSE was positively correlated with QoL (r = 0.758) and negatively correlated with depressive symptoms (r = -0.496). QoL was negatively associated with depressive symptoms (r = -0.535).

Table 1. Demographic and clinical information.

**Variables**	**N (%)/mean±SD**
**Age, mean (SD)**	61.4 (11.9)
**Gender, N (%)**	
Male	438 (65.4)
Female	232 (34.6)
**Time since stroke, mean (SD)**	143.8 (530.2)
**Type of stroke, N (%)**	
Ischemic	556 (83.0)
Hemorrhagic	101 (15.1)
Others	13 (1.9)
**Paretic side, N (%)**	
Left	292 (43.6)
Right	297 (44.3)
Unknown	81 (12.1)
**Dysfunction, N (%)**	
Limb functional disorder	571 (85.2)

Speech dysfunction	223 (33.3)
Cognitive disorder	15 (2.2)
Balance disorder	52 (7.8)
**Complication, N (%)**	
Hypertension	459 (68.5)
Diabetes	76 (11.3)
Cancer	2 (0.3)
**Previous history, N (%)**	
Operation History	6 (0.9)
Stroke History	153 (22.8)
**Assessment scales, mean (SD)**	
Mini-BEST	24.17（7.247）
BRS	15.18 (3.346)
ABC, Median (inter-quartile range)	88.13（64.38 95.63）[a]
Barthel index	91.21（13.740）
HAM-D	3.67 (4.68)

ABC: Activity-specific Balance Confidence; HAM-D: Hamilton Depression Scale; Mini-BEST: Mini-Balance Evaluation Systems Test; BRS: Brunnstrom; [a]: Median and inter-quartile range.

Table 2. Correlations among variables.

	**BRS**	**Mini-BEST**	**BSE**	**QoL**	**Depressive Symptoms**	**Mean**	**SD**
**BRS**	1					15.18	3.346
**Mini-BEST**	0.696[aa]	1				24.17	7.247
**BSE**	0.644[aab]	0.789[aab]	1			76.90	25.61
**QoL**	0.633[aa]	0.701[aa]	0.758[aa]	1		91.21	13.74
**Depressive Symptoms**	-0.438[aa]	-0.492[aa]	-0.496[aa]	0.535[aa]	1	3.67	4.68

[aa]: p <0.001; [b]: Spearman correlation.

BRS: Brunnstrom; BSE: Balance Self-efficacy; QoL: Quality of Life; Mini-BEST: Mini-Balance Evaluation Systems Test; SD: Standard Deviation.

Fitting of the theoretical model

The SEM analysis showed that the study model fit indices indicated satisfactory fit (χ^2 = 14.233, χ^2/df = 4.744, *p* =0.002. RMSEA = 0.075, SRMR = 0.010, CFI = 0.994, TLI = 0.981).

Path analysis

As shown in Fig 1, physical capability was the independent variable; BSE and depressive symptoms were the mediators and QoL was the dependent variable in the multiple mediation model. The standardized path coefficients, interpreted similarly to regression coefficients, are presented in Fig 2. All the pathways between physical capability and QoL were significant in the model. This model explained a total of 66.4% of the variance in QoL. Physical capability was a positive predictive factor of QoL (β = 0.470, $p < 0.000$). BSE contributed most to the total variance of QoL (71.8%), while Depressive symptoms accounted for 31.6% of the variance of QoL. The mediator of BSE positively predicted QoL (β = 0.299, $p < 0.000$). However, depressive symptoms were the negative predictor of QoL (β = -0.122, $p < 0.000$). Additionally, physical capability positively predicted self-efficacy (β = 0.847, $p < 0.000$) but was a negative of depressive symptoms (β = -0.562, $p < 0.000$) (Table 3).

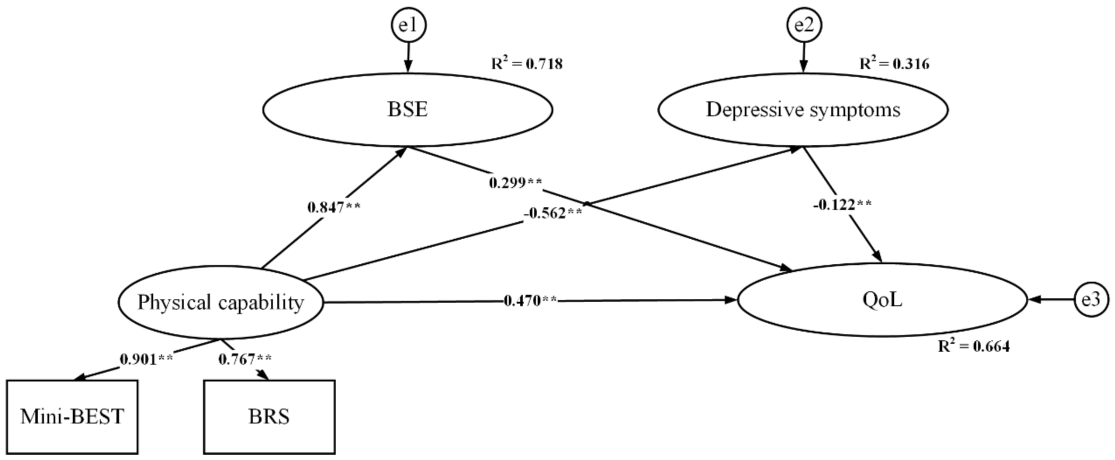

Fig 2. Model fit (χ^2 = 14.233, χ^2/df = 4.744, *p* = 0.002. RMSEA = 0.075, SRMR = 0.010, TLI = 0.981, CFI = 0.994). Mini-BEST: mini-Balance Evaluation Systems Test; BRS: Brunnstrom; BSE: Balance self-efficacy; QoL: Quality of Life. This model is consistent with hypotheses 1 and 2 of the theoretical model. BSE and Depressive symptoms are mediating variables between Physical capability and QoL.

Table 3. Path analysis.

**Variables**	**Point estimate**		**Product of coefficients**		***p***
	**B**	***β***	**SE**	**Z**	
Physical capability---> QoL	0.989	0.470	0.143	6.916	0.000 [aa]
Physical capability---> BSE	3.324	0.847	0.124	26.806	0.000 [aa]
Physical capability---> Depressive symptoms	-0.403	-0.562	0.026	-15.500	0.000 [aa]
BSE---> QoL	0.16	0.299	0.031	5.161	0.000 [aa]
Depressive symptoms---> QoL	-0.357	-0.122	0.087	-4.103	0.000 [aa]

B: Unstandardized coefficients; *β*: Standardized coefficients; SE: Standard error; QoL: Quality of Life; BSE: Balance Self-efficacy; [aa]: $p < 0.001$.

Mediation effect analysis

As shown in Table 4, we found significant total, direct, and indirect effects in the multiple mediation model. The specific indirect effect of physical capability on QoL via BSE was statistically significant (*β* = 0.254, *p* = 0.003). However, the other mediations of depressive symptoms were not statistically significant (*β* = 0.069, *p* =

0.136). These findings negated the hypothesis that depressive symptoms mediate the relationship between physical capability and QoL. Thus, BSE significantly mediates the association between physical capability and QoL (β = 0.322, p = 0.002).

Table 4. The total, direct, and indirect effects.

**Effects**	**Variables**	**Point estimate**		**Product of coefficients**		**Bias-corrected percentile method**		***p***
		**B**	***β***	**SE**	**Z**	**Lower bounds**	**Upper bounds**	
Total effect	Physical capability--->QoL	4.242	0.793	0.265	16.108	3.756	4.805	0.000[aa]
Direct effect	Physical capability--->QoL	2.517	0.470	0.610	4.125	1.325	3.720	0.000[aa]
Total indirect effect	Physical capability---> BSE--->QoL<br>Physical capability--->Depressive symptoms--->QoL	1.725	0.322	0.559	3.086	0.693	2.887	0.002[a]
Specific indirect effect	Physical capability--->BSE--->QoL	1.357	0.254	0.456	2.976	0.497	2.291	0.003[a]
Specific indirect effect	Physical capability--->Depressive symptoms--->QoL	0.368	0.069	0.247	1.490	-0.069	0.891	0.136

B: Unstandardized coefficients; β: Standardized coefficients; SE: Standard error; Z: Z-

value; QoL: Quality of Life; BSE: Balance Self-efficacy; [a]: p<0.05; [aa]: p<0.001.

Moderation effect analysis

The analysis presented in Table 5 shows that hypertension significantly moderated the indirect effect of physical capability on QoL (B = -0.953, p = 0.014). We examined the moderation effect using the pick-a-point approach to estimate the conditional direct effects of physical capability on QoL via BSE and depressive symptoms on QoL at different levels of hypertension. The results indicated that the interaction terms (physical capability × hypertension and depressive × hypertension) in the pathways from physical capability to QoL through depressive symptoms were significant (B = 0.412, p = 0.015; B = 0.831, p = 0.020, respectively). The plots of the interactions between physical capability, depressive symptoms, and QoL are presented in Fig 3. In all, hypertension significantly moderates the association between physical capability and QoL toward depressive symptoms, supporting our hypothesis. However, diabetes and gender showed no significant effect on the indirect path (B = 0.943, p = 0.570; B = 0.022, p = 0.961, respectively). Thus, these findings do not support the hypothesis that diabetes/gender moderates the aforementioned association.

Table 5. Moderated analysis.

**Variables**	**Point estimate**	**Product of coefficients**		**Bias-corrected percentile method**		***p***
	**B**	**SE**	**Z**	**Lower bounds**	**Upper bounds**	
Hypertension	-0.953	0.404	-2.359	-1.793	-0.215	0.014 [a]
Physical capability-->BSE	-0.956	0.925	-1.034	-2.765	0.844	0.301

BSE--->QoL	-0.192	0.103	-1.862	-0.406	0.192	0.063
Physical capability-->Depressive symptoms	0.412	0.169	2.442	0.08	0.132	0.015 [a]
Depressive symptoms-->QoL	0.831	0.357	2.325	0.2	1.575	0.020 [a]
Physical capability-->QoL	2.897	1.239	2.339	0.574	5.462	0.019 [a]
Diabetes	0.943	1.660	0.568	-1.455	3.683	0.570
Gender	0.022	0.476	0.046	-0.835	1.015	0.961

B: Unstandardized coefficients; β: Standardized coefficients; SE: Standard error; Z: Z-value; QoL: Quality of Life; BSE: Balance Self-efficacy; [a]: $p<0.05$.

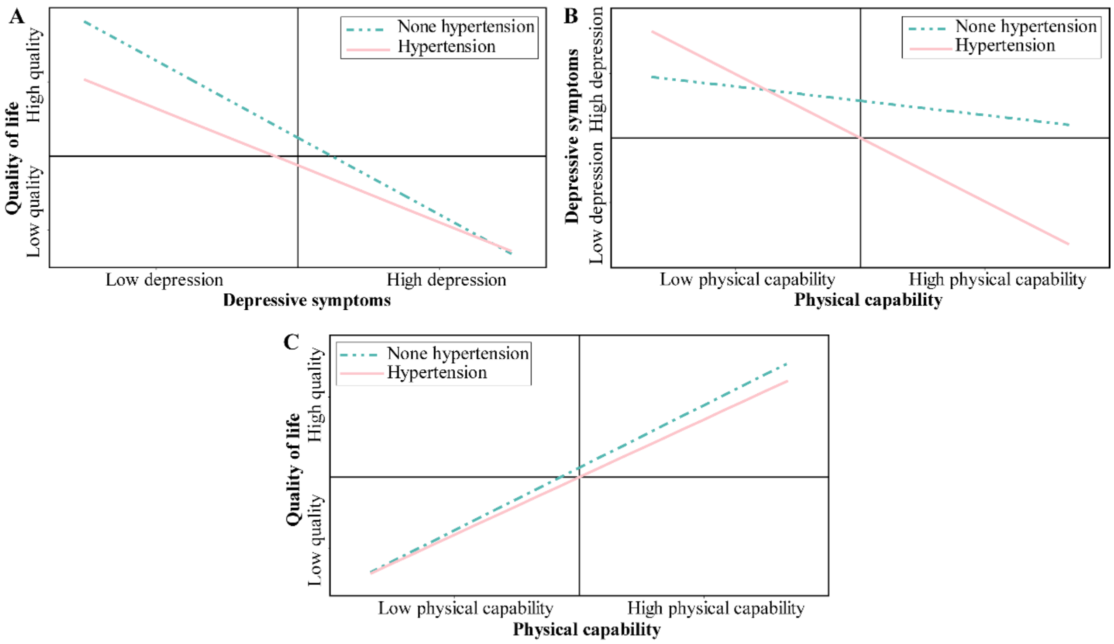

Fig 3. Interactive effect of hypertension on the relationship between physical capability, QoL, and depressive symptoms. This is consistent with the assumptions of Theoretical Model 3. Hypertension acts as a moderating variable between Physical capability and QoL.

Discussion

This study primarily explored a multiple mediation model of the ‘physical capability to QoL via BSE and depressive symptoms’ pathway in a group of stroke patients from China based on the Wilson and Cleary model. Our findings are consistent with a previous study, showing the significant mediation effect of BSE on the relationship between physical capability and QoL [20]. As hypothesized, the physical capability showed an indirect positive correlation with QoL through BSE; a higher level of physical capability indicated higher BSE, thereby improving the patient’s QoL.

However, we found no mediation effect of depressive symptoms on the relationship between physical capability and QoL. According to French et al. [19], depressive symptoms moderate the relationships between physical capability, self-efficacy, and participation in stroke patients. Although the hypothesis about depressive symptoms mediating the relationship between physical capability and QoL was not supported, all the paths in our model were significant. The present findings suggest that a high level of physical capability can predict a lower severity of depressive symptoms and a better QoL, while a high severity of depressive symptoms predicts a lower QoL. This supports our hypothesis.

Our results also demonstrated that hypertension moderated the relationship between physical capability, depressive symptoms, and QoL in stroke patients. The patients with hypertension had decreased physical capability, which in turn increased depressive symptoms. This result is consistent with Sok et al. showing a strong association between depressive symptoms and hypertension [35]. Accordingly, another study reported that depressed patients with hypertension had poor control of blood pressure [53]. The possibility exists that antidepressant therapy interferes with blood pressure control in hypertension patients, but further research on this topic is warranted. Additionally, a recent study showed that increased walking (physical) activity lowers hypertension risk in postmenopausal women [54]. Our study also indicated that physical activity positively influences hypertension risk, even in a mixed-gender population. Conversely,

patients with high blood pressure were found to be less active than those without hypertension. Furthermore, QoL negatively correlates with stroke risk factors such as hypertension [55, 56]. In general, hypertension is an important moderator of relationships between physical capability, depressive symptoms, and QoL. However, there is a lack of literature examining the relationship between hypertension and BSE, which must be explored in both a qualitative and quantitative manner. Similarly, we found no evidence that hypertension moderated the 'physical capability to QoL via BSE' pathway. Although our findings did not show that diabetes and gender moderated the relationship between physical capability and QoL, this may be attributable to the relatively small number of individuals with diabetes and females in this cohort. Therefore, further research is needed with a larger and more diverse sample. The theoretical model in this study was built based on a previous model suggesting that BSE is a significant mediator and hypertension has a moderating effect on the 'physical capability to QoL through depressive symptoms' pathway. The mediating effect of BSE and the moderating effect of hypertension may help interpret the mechanisms of the relationship between physical capability and QoL. Our conceptual model facilitates the understanding of these associations to make targeted strategies promoting QoL and rehabilitation. Patients show improved balance function, which naturally enhances balance confidence, leading to a significant improvement in QoL [18]. Health professionals should incorporate interventions to enhance balance confidence and the control of blood pressure in their management of physical and mental rehabilitation.

Limitations

There are some limitations to our research. First, we did not consider the complex nature of the QoL index, which may complicate relationships among the various missing data of clinical variables. Researchers should implement rigorous strategies to minimize missing data and place greater emphasis on exploring the intricacies of the QoL index in more detail, taking into account its multiple dimensions. Second, we did

not take into account the impact of stroke subtypes, a possible confounding parameter in this study. Future research should consider stratifying stroke patients by subtype, which would facilitate a more precise analysis of how different stroke types may influence the outcomes. Third, the cross-sectional study design restricts the interpretation and generalization of our results. An additional longitudinal study may help verify the conclusions of this study.

Conclusion

This study provided a better insight into the relationship between physical capability and QoL via BSE in stroke patients, which may help establish appropriate treatment for these individuals. Our results uncovered the underlying moderating effect of hypertension on relationships between physical capability, depressive symptoms, and QoL. An effective rehabilitation strategy can achieve concrete targets for stroke risk patients via BSE, improving their QoL [57].

Acknowledgment

The authors would like to thank all the participants in the study and MJEditor (www.mjeditor.com) for their linguistic assistance during the preparation of this manuscript.

Author Contributions

Conceptualization: Xue Yang.

Formal analysis: Qian Liu.

Investigation: Yihuan Lu.

Methodology: Xue Yang.

Supervision: Liqing Yao.

Validation: Xue Yang, Hongmei Zhang, Qian Liu.

Writing – original draft: Xue Yang.

Writing – review & editing: Xue Yang, Hongmei Zhang.

Consent to participate

Written informed consent was obtained from all patients before participation.

Reference

1. Global, regional, and national burden of stroke, 1990-2016: a systematic analysis for the Global Burden of Disease Study 2016 Lancet Neurol, 2019. 18 (5): p.439-58. https://10.1016/s1474-4422(19)30034-1 PMID: 30871944
2. Chen Z, Jiang B, Ru X, Sun H, Sun D, Liu X, et al., Mortality of Stroke and Its Subtypes in China: Results from a Nationwide Population-Based Survey Neuroepidemiology, 2017. 48 (3-4): p.95-102. https://10.1159/000477494 PMID: 28586776
3. Kim A, Cahill E, Cheng N. Global Stroke Belt: Geographic Variation in Stroke Burden Worldwide Stroke, 2015. 46 (12): p.3564-70. https://10.1161/STROKEAHA.115.008226 PMID: 26486867
4. Jurgens C, Lee C, Aycock D, Masterson Creber R, Denfeld Q, DeVon H, et al., State of the Science: The Relevance of Symptoms in Cardiovascular Disease and Research: A Scientific Statement From the American Heart Association Circulation, 2022. 146 (12): p.e173-e84. https://10.1161/CIR.0000000000001089 PMID: 35979825
5. Labeit B, Michou E, Hamdy S, Trapl-Grundschober M, Suntrup-Krueger S, Muhle P, et al., The assessment of dysphagia after stroke: state of the art and future directions Lancet Neurol, 2023. 22 (9): p.858-70. https://10.1016/S1474-4422(23)00153-9 PMID: 37596008
6. Braun L, Grady K, Kutner J, Adler E, Berlinger N, Boss R, et al., Palliative Care and Cardiovascular Disease and Stroke: A Policy Statement From the American Heart Association/American Stroke Association Circulation, 2016. 134 (11): p.e198-e225. https://10.1161/CIR.0000000000000438 PMID: 27503067
7. Turana Y, Tengkawan J, Chia Y, Nathaniel M, Wang J, Sukonthasarn A, et al., Hypertension and stroke in Asia: A comprehensive review from HOPE Asia J Clin Hypertens (Greenwich), 2021. 23 (3): p.513-21. https://10.1111/jch.14099 PMID: 33190399
8. Nor A M N, Noor A A A, Saperi S, Syed M A. Functional limitation and health related quality of life and associated factors among long term stroke survivors in a

Malaysian community Med J Malaysia, 2016. 71 (6): p.313-21.
9. Haghgoo H, Pazuki E, Hosseini A, Rassafiani M. Depression, activities of daily living and quality of life in patients with stroke J Neurol Sci, 2013. 328 (1-2): p.87-91. https://10.1016/j.jns.2013.02.027 PMID: 23522526
10. Muren M, Hutler M, Hooper J. Functional capacity and health-related quality of life in individuals post stroke Top Stroke Rehabil, 2008. 15 (1): p.51-8. https://10.1310/tsr1501-51 PMID: 18250074
11. Franco F, Fay H, Marco G, Antonio N, Andrea G. Using psychometric techniques to improve the Balance Evaluation Systems Test: the mini-BESTest J Rehabil Med, 2010. 42 (4): p.323-31. https://10.2340/16501977-0537 PMID: 20461334
12. Naghdi S, Ansari N, Mansouri K, Hasson S. A neurophysiological and clinical study of Brunnstrom recovery stages in the upper limb following stroke Brain Inj, 2010. 24 (11): p.1372-8. https://10.3109/02699052.2010.506860 PMID: 20715900
13. Xue Y, Yutong F, Hong F, Jibing O, Jimin C, Liqing Y. Exploring the complex pathways between somatosensory impairment, physical ability and affective symptoms following stroke: mediation analyses Annals of Palliative Medicine, 2022. 11 p.2395-409.
14. Di Monaco M, Trucco M, Di Monaco R, Tappero R, Cavanna A. The relationship between initial trunk control or postural balance and inpatient rehabilitation outcome after stroke: a prospective comparative study Clin Rehabil, 2010. 24 (6): p.543-54. https://10.1177/0269215509353265 PMID: 20511303
15. Arlene A S, Marieke Van P, Peter A A, Kristine K M, Stephanie A C, Stephen J P. Balance is associated with quality of life in chronic stroke Top Stroke Rehabil, 2013. 20 p.340-6.
16. Schmid A, Van Puymbroeck M, Knies K, Spangler-Morris C, Watts K, Damush T, et al., Fear of falling among people who have sustained a stroke: a 6-month longitudinal pilot study The American journal of occupational therapy : official publication of the American Occupational Therapy Association, 2011. 65 (2): p.125-32. https://10.5014/ajot.2011.000737 PMID: 21476359
17. Arlene A S, Sarah E A, Valerie A J, M Jane R, Stephanie A S, Marieke Van P. Fear of falling in people with chronic stroke The American journal of occupational therapy : official publication of the American Occupational Therapy Association, 2015. 69 (3): p.6903350020. https://10.5014/ajot.2015.016253 PMID: 25871606
18. Caryne T, Krista L B, William C M, Janice J E. Balance Confidence: A Predictor of Perceived Physical Function, Perceived Mobility, and Perceived Recovery 1 Year After Inpatient Stroke Rehabilitation Arch Phys Med Rehabil, 2016. 97 (7): p.1064-71. https://10.1016/j.apmr.2016.03.004 PMID: 27060032
19. Margaret A F, Allison M, Ryan T P, Darcy S R. Depressive Symptoms Moderate the Relationship Among Physical Capacity, Balance Self-Efficacy, and Participation in People After Stroke Phys Ther, 2021. 101 (12): p.1-10. https://10.1093/ptj/pzab224 PMID: 34636909
20. Margaret A F, Meghan F M, Ryan P, Darcy R. Self-efficacy Mediates the Relationship between Balance/Walking Performance, Activity, and Participation after Stroke Top Stroke Rehabil, 2016. 23 (2): p.77-83.

https://10.1080/10749357.2015.1110306 PMID: 26653764
21. Yannis Yan L, Lisha W, Ying Y, Yangkun C, Vincent C T M, Gabor S U, et al., Association Between Behavioral Dysexecutive Syndrome and the Health-Related Quality of Life Among Stroke Survivors Front Psychiatry, 2020. 11 p.563930. https://10.3389/fpsyt.2020.563930 PMID: 33101083
22. Hellstrom K, Lindmark B, Wahlberg B, Fugl-Meyer AR. Self-efficacy in relation to impairments and activities of daily living disability in elderly patients with stroke: a prospective investigation J Rehabil Med, 2003. 35 (5): p.202-7. https://10.1080/16501970310000836 PMID: 14582550
23. Szczepanska-Gieracha J, Mazurek J. The Role of Self-Efficacy in the Recovery Process of Stroke Survivors Psychol Res Behav Manag, 2020. 13 p.897-906. https://10.2147/PRBM.S273009 PMID: 33177896
24. Koleck M, Gana K, Lucot C, Darrigrand B, Mazaux JM, Glize B. Quality of life in aphasic patients 1 year after a first stroke Qual Life Res, 2017. 26 (1): p.45-54. https://10.1007/s11136-016-1361-z PMID: 27405871
25. Wei-Quan L, Ting-Yuan H, Lan L, Yun-Ou Y, Yao-Hui L, Min-Ying S, et al., Prevalence and related factors of depression and falls among the elderly living in rural communities of Guangzhou Psychology, health & medicine, 2020. 25 (8): p.980-8. https://10.1080/13548506.2020.1714064 PMID: 31967487
26. Lin C, Babiker A, Srdanovic N, Kocherginsky M, Harvey RL. Depressive symptoms after stroke are associated with worse recovery International journal of psychiatry in medicine, 2020. 55 (4): p.227-38. https://10.1177/0091217420905459 PMID: 32050815
27. León LPd, Mangin JPL, Ballesteros S. Psychosocial Determinants of Quality of Life and Active Aging. A Structural Equation Model International Journal of Environmental Research and Public Health, 2020. 17 (17): https://10.3390/ijerph17176023
28. R G Robinson, L B Starr, K L Kubos, T R Price. A Two-Year Longitudinal Study of Post-Stroke Mood Disorders: Findings During the Initial Evaluation Stroke, 1983. 14 p.736-41.
29. Heiland EG, Welmer AK, Wang R, Santoni G, Fratiglioni L, Qiu C. Cardiovascular Risk Factors and the Risk of Disability in Older Adults: Variation by Age and Functional Status J Am Med Dir Assoc, 2019. 20 (2): p.208-12 e3. https://10.1016/j.jamda.2018.05.013 PMID: 30006016
30. Tusa BS, Geremew BM, Tefera MA. Heath related quality of life and associated factors among adults with and without diabetes in Adama city East Shewa, Ethiopia 2019; using generalized structural equation modeling Health Qual Life Outcomes, 2020. 18 (1): p.83. https://10.1186/s12955-020-01337-9 PMID: 32228598
31. Li ZH, Lv YB, Kraus VB, Yin ZX, Liu SM, Zhang XC, et al., Trends in the Incidence of Activities of Daily Living Disability Among Chinese Older Adults From 2002 to 2014 J Gerontol A Biol Sci Med Sci, 2020. 75 (11): p.2113-8. https://10.1093/gerona/glz221 PMID: 31603986
32. Qian J, Ren X. Association between comorbid conditions and BADL/IADL disability in hypertension patients over age 45: Based on the China health and

retirement longitudinal study (CHARLS) Medicine (Baltimore), 2016. 95 (31): p.e4536. https://10.1097/MD.0000000000004536 PMID: 27495110

33. Katan M, Luft A. Global Burden of Stroke Seminars in neurology, 2018. 38 (2): p.208-11. https://10.1055/s-0038-1649503 PMID: 29791947

34. Zhang XP, Pan JH, Wan LH, Liu ZY, Mo MM, Wang MY, et al., Factors influencing health behaviour, blood pressure control, and disability in hypertensive ischaemic stroke patients after a comprehensive reminder intervention J Adv Nurs, 2020. 76 (6): p.1384-93. https://10.1111/jan.14340 PMID: 32128865

35. Sok SR, Yim E, Sim H, Sim HJ. Relationships among Self-Care Competency, Presence of Depressive Symptom, and Health-Related Quality of Life of Korean Stroke Patients Clin Nurs Res, 2021. 30 (5): p.670-9. https://10.1177/1054773820913984 PMID: 32207330

36. Kristopher J P, Derek D R, Andrew F H. Addressing moderated mediation hypotheses. Theory, methods, and prescriptions Multivariate Behav Res, 2007. 42 p.185-227.

37. Ira B. Wilson, Paul D. Cleary. Linking Clinical Variables With Health-Related Quality of Life JAMA, 1995. 273 p.59-65.

38. Saengsiri AO, Hacker ED. Conducting quality of life research in people with coronary artery disease in non-English-speaking countries: conceptual and operationalization issues J Cardiovasc Nurs, 2015. 30 (1): p.74-84. https://10.1097/JCN.0000000000000116 PMID: 24365869

39. Association WM. World Medical Association Declaration of Helsinki Ethical Principles for Medical Research Involving Human Subjects JAMA, 2013. 310 p.2191-4.

40. Sawner K JL. Brunnstrom movement therapy in hemiplegia: a neurophysilogical approach. 2nd ed. Philadelphia: JB Lippincott; 1992.

41. Huang CY, Lin GH, Huang YJ, Song CY, Lee YC, How MJ, et al., Improving the utility of the Brunnstrom recovery stages in patients with stroke: Validation and quantification Medicine (Baltimore), 2016. 95 (31): p.e4508. https://10.1097/MD.0000000000004508 PMID: 27495103

42. Tsang CS, Liao LR, Chung RC, Pang MY. Psychometric properties of the Mini-Balance Evaluation Systems Test (Mini-BESTest) in community-dwelling individuals with chronic stroke Phys Ther, 2013. 93 (8): p.1102-15. https://10.2522/ptj.20120454 PMID: 23559522

43. Florence I. Mahoney DWB. Functional evaluation: the Barthel index Maryland State Medical Journal, 1965. 14 p.56-61.

44. Collin C, Wade D T, Davies S, Horne V. The Barthel ADL Index: a reliability study Int Disabil Srudies, 1988. 10 p.61-3.

45. Powell L E, Myers A M. The Activities-specific Balance Confidence (ABC) Scale Journal of Gerontology, 1995. 50A p.M28-M34.

46. Zhang D, Tian F, Gao W, Huang Y, Huang H, Tan L. The Chinese Short Version of the Activities-Specific Balance Confidence Scale: Its Validity, Reliability, and Predictive Value for Future Falls in Community-Dwelling Older Adults Clin Interv Aging, 2022. 17 p.1483-91. https://10.2147/CIA.S380921 PMID: 36212511

47. Fava G, Kellner R, Munari F, Pavan L. The Hamilton Depression Rating Scale in normals and depressives Acta psychiatrica Scandinavica, 1982. 66 (1): p.26-32. https://10.1111/j.1600-0447.1982.tb00911.x PMID: 7124430

48. Matthias J. Mu¨ller AD. Standardized rater training for the Hamilton Depression Rating Scale (HAMD-17) in psychiatric novices J Affect Disord, 2003. 77 p.65 – 9. https://10.1016/S0165-0327(02)00097-6

49. Linda K. Muthen BOM. Mplus User's Guide. Los Angeles: CA: Muth´en & Muth´en; 2012.

50. Yiran D, Chao-Ying J P. Principled missing data methods for researchers SpringerPlus, 2013. 2 p.222.

51. Daire Hooper J C, Michael R M. Structural Equation Modelling Guidelines for Determining Model Fit Journal of Business Research Methods, 2008. 6 p.53-60.

52. Kline R B. Principles and Practice of Structual Equation Modeling, 4th ed 4th ed. New York: Guilford Press; 2016.

53. Scalco A Z, Mônica Z S, João B S A, Francisco L N. hypertension and depression CLINICS, 2005. 60 p.241-50.

54. Miller C R, Wactawski-Wende J, Manson J E, Haring B, Hovey K M, Laddu D, et al., Walking Volume and Speed Are Inversely Associated With Incidence of Treated Hypertension in Postmenopausal Women Hypertension, 2020. 76 (5): p.1435-43. https://10.1161/HYPERTENSIONAHA.120.15839 PMID: 32981366

55. Yao H, Zhang J, Wang Y, Wang Q, Zhao F, Zhang P. Stroke risk and its association with quality of life: a cross-sectional study among Chinese urban adults Health Qual Life Outcomes, 2021. 19 (1): p.236. https://10.1186/s12955-021-01868-9 PMID: 34627278

56. Alotaibi S M, Alotaibi H M, Alolyani A M, Abu Dali F A, Alshammari A K, Alhwiesh A A, et al., Assessment of the stroke-specific quality-of-life scale in KFHU, Khobar: A prospective cross-sectional study Neurosciences (Riyadh), 2021. 26 (2): p.171-8. https://10.17712/nsj.2021.2.20200126 PMID: 33814370

57. Silva S M, Correa F I, Faria C D, Pereira G S, Attie E A, Correa J C. Reproducibility of the items on the Stroke Specific Quality of Life questionnaire that evaluate the participation component of the International Classification of Functioning, Disability and Health Disabil Rehabil, 2016. 38 (24): p.2413-8. https://10.3109/09638288.2015.1130178 PMID: 26879175

Dear editors and reviewers:

Re: Manuscript ID: PONE-D-24-45706 and Title: Mediation and moderation analysis of the association between physical capability and quality of life among stroke patients

Thank you for your letter and the reviewers' comments concerning our manuscript entitled "PONE-D-24-45706" (ID). Those comments are valuable and very helpful. We have read through the comments carefully and have made corrections. Based on the instructions provided in your letter, we uploaded the file of the revised manuscript. Revisions in the main text are highlighted in yellow as additions or modifications. The responses to the reviewer's comments are marked in yellow and presented following.

We would love to thank you for allowing us to resubmit a revised copy of the manuscript and we highly appreciate your time and consideration.

Sincerely.
Liqing-Yao

Editorial opinion:
1) ***If applicable, we recommend that you deposit your laboratory protocols in protocols.io to enhance the reproducibility of your results. Protocols.io assigns your protocol its identifier (DOI) so that it can be cited independently in the future. For instructions see: https://journals.plos.org/plosone/s/submission-guidelines#loc-laboratory-protocols. Additionally, PLOS ONE offers an option for publishing peer-reviewed Lab Protocol articles, which describe protocols hosted on protocols.io. Read more information on sharing protocols at https://plos.org/protocols?utm_medium=editorial-email&utm_source=authorletters&utm_campaign=protocols.***

Response: Thanks for the editorial suggestions.

Journal Requirements:

1)***Please ensure that your manuscript meets PLOS ONE's style requirements, including those for file naming. The PLOS ONE style templates can be found at**

https://journals.plos.org/plosone/s/file?id=wjVg/PLOSOne_formatting_sample_main_body.pdf and https://journals.plos.org/plosone/s/file?id=ba62/PLOSOne_formatting_sample_title_authors_affiliations.pdf

Response: Thanks to the editorial advice, I have downloaded the PLOS ONE style

template and have modified it in this way, e.g. the title page in the manuscript as well.

Mediation and moderation analysis of the association between physical capability and quality of life among stroke patients

Xue Yang [1¶], Hongmei Zhang [1¶], Yihuan Lu [1], Qian Liu [1], Liqing Yao [1*]

1 Department of Rehabilitation Medicine, the Second Affiliated Hospital of Kunming Medical University, Kunming, Yunnan, China

2)* **Thank you for stating the following financial disclosure:**

"The work was supported by the Major Science and Technology Project of Yunnan Province (Grant numbers[2018zf016]), Rehabilitation Clinical Medical Centre of Yunnan Province (Grant numbers[zx2019-04-02]), National Key Research and Development Program (Grant numbers[2018YFC2002301]), and Jiajie Expert Workstation of Yunnan Province (Grant numbers[2019IC034]), and Kunming Medical University Postgraduate Innovation Fund (Grant numbers[2022B11])."

Please state what role the funders took in the study. If the funders had no role, please state: "The funders had no role in study design, data collection and analysis, decision to publish, or preparation of the manuscript." If this statement is not correct you must amend it as needed.

Please include this amended Role of Funder statement in your cover letter; we will change the online submission form on your behalf.

Response: We have clarified the role of the funder in the study and have removed the funding section from the manuscript and added a funding section to the cover letter. This corresponds to lines 12 to 19 of the cover letter.

The work was supported by the Major Science and Technology Project of Yunnan Province (Grant numbers[2018zf016]), Rehabilitation Clinical Medical Centre of Yunnan Province (Grant numbers[zx2019-04-02]), National Key Research and Development Program (Grant numbers[2018YFC2002301]), and Jiajie Expert Workstation of Yunnan Province (Grant numbers[2019IC034]), and Kunming Medical University Postgraduate Innovation Fund (Grant numbers[2022B11]). The funders had no role in study design, data collection and analysis, decision to publish, or preparation of the manuscript.

3)***Please note that funding information should not appear in the Acknowledgments section or other areas of your manuscript. We will only publish funding information present in the Funding Statement section of the online submission form. Please remove any funding-related text from the manuscript.**

Response: We have removed any fund-related text from the manuscripts. We have added a funding section to the cover letter. This corresponds to lines 12 to 19 of the cover letter. This corresponds to lines 12 to 19 of the cover letter.

The work was supported by the Major Science and Technology Project of Yunnan Province (Grant numbers[2018zf016]), Rehabilitation Clinical Medical Centre of Yunnan Province (Grant numbers[zx2019-04-02]), National Key Research and Development Program (Grant numbers[2018YFC2002301]), and Jiajie Expert Workstation of Yunnan Province (Grant numbers[2019IC034]), and Kunming Medical University Postgraduate Innovation Fund (Grant numbers[2022B11]). The funders had no role in study design, data collection and analysis, decision to publish, or preparation of the manuscript.

4)* **We note that your Data Availability Statement is currently as follows:**

“All relevant data are within the manuscript and its Supporting Information files.”

Please confirm at this time whether or not your submission contains all the raw data required to replicate the results of your study. Authors must share the “minimal data set” for their submission. PLOS defines the minimal data set to consist of the data required to replicate all study findings reported in the article, as well as related metadata and methods (https://journals.plos.org/plosone/s/data-availability#loc-minimal-data-set-definition).

For example, authors should submit the following data:

- The values behind the means, standard deviations, and other measures reported;
- The values used to build graphs;
- The points extracted from images for analysis.

Authors do not need to submit their entire data set if only a portion of the data was used in the reported study.

If your submission does not contain these data, please either upload them as Supporting Information files or deposit them to a stable, public repository and provide us with the relevant URLs, DOIs, or accession numbers. For a list of recommended repositories, please see https://journals.plos.org/plosone/s/recommended-repositories.

If there are ethical or legal restrictions on sharing a de-identified data set, please explain them in detail (e.g., data contain potentially sensitive information, data are owned by a third-party organization, etc.) and who has imposed them (e.g., an ethics committee). Please also provide contact information for a data access committee, ethics committee, or other institutional body to which data requests may be sent. If data are owned by a third party, please indicate how others may request data access.

Response: Thanks to the suggestion of the editorial team, we have uploaded the supporting data files named "data.xlsx" and "Mplus_moderation.xlsx". The "data.xlsx" file is the full information of the 670 patients we included, while "Mplus_moderation.xlsx" is used to draw the patient information for structural equation modeling.

5)***PLOS requires an ORCID iD for the corresponding author in Editorial Manager on papers submitted after December 6th, 2016. Please ensure that you have an ORCID iD and that it is validated in Editorial Manager. To do this, go to 'Update my Information' (in the upper left-hand corner of the main menu), and click on the Fetch/Validate link next to the ORCID field. This will take you to the ORCID site and allow you to create a new ID or authenticate a pre-existing iD in Editorial Manager.**

Response: Thanks to the editorial board's suggestion, we submitted this manuscript using the corresponding author's ORCID iD login to the PLOS ONE website.

6)***Your ethics statement should only appear in the Methods section of your manuscript. If your ethics statement is written in any section besides the Methods, please delete it from any other section.**

Response: I have removed any part of the ethics statement written outside of the method.

7)* **Please include a separate caption for each figure in your manuscript.**

Response: I have captioned each figure separately in the manuscript as shown in Figure 1.

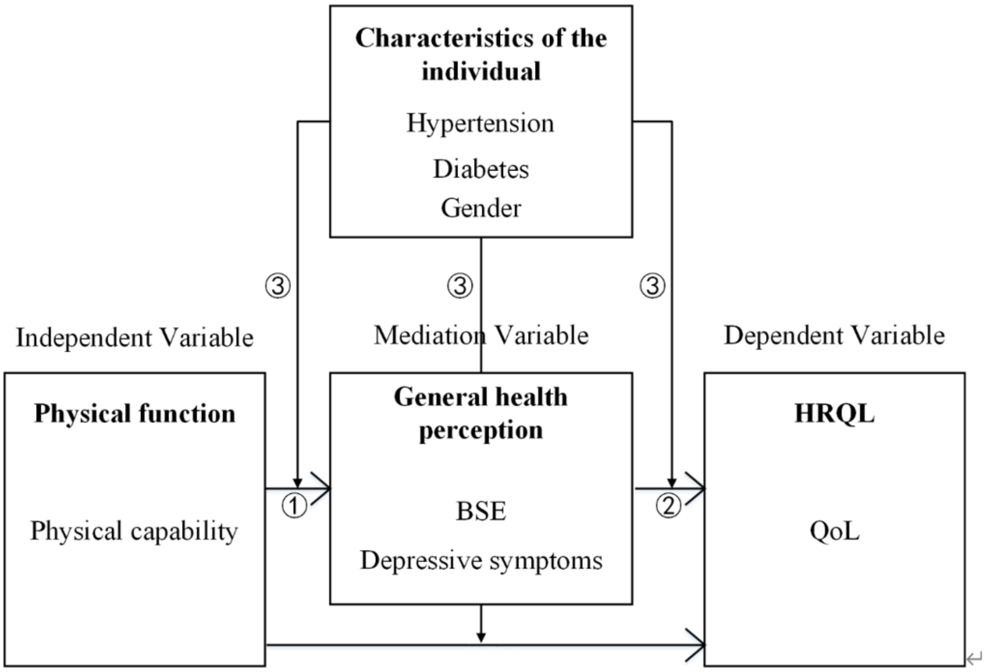

Fig 1. The theoretical model is based on Wilson and Cleary's model. ①: Hypothesis 1 of the current study; ②: Hypothesis 2 of the current study; ③: Hypothesis 3 of the current study. BSE, Balance Self-Efficacy; HRQL, Health-Related Quality of Life; QoL, Quality of Life. The arrows represent the hypothesized directional relationships between the variables in the model.

8)* **Please review your reference list to ensure that it is complete and correct. If you have cited papers that have been retracted, please include the rationale for doing so in the manuscript text, or remove these references and replace them with relevant current references. Any changes to the reference list should be mentioned in the rebuttal letter that accompanies your revised manuscript. If you need to cite a retracted article, indicate the article's retracted status in the References list and also include a citation and full reference for the retraction notice.**

Response: I have checked the reference list.

Reviewer #1:

1)***The manuscript was well written. There are a few minor corrections to be made. These are in the track-changed document attached.**
It will be great to discuss how stroke affects the quality of life of stroke survivors. Also, the results on BRS, MiniBest, QoL, BSE, and depression have to be presented first before the correlations.

Response: We thank the reviewers for their suggestions and we have revised this section.

aging population [2, 3]. Stroke decreases the survivors' quality of life (QoL) because it can cause physical and cognitive impairments, emotional distress, and fatigue[4, 5].

2

Additionally, it can also lead to social isolation and a loss of independence[6]. Therefore, it is a serious health problem worldwide, especially in Asia [7]. The reduction in QoL

The median score of the ABC was 88.13, whereas the mean assessment scales of mini-

8

BEST, BRS, BI, and HAM-D are outlined in Table 1. We found significant correlations

Reviewer #2:
1)***Throughout the document, 'capacity' and 'capability' are used interchangeably. Although these words do have the same meaning, it will be easier to read and process the information if the same term is used throughout (I would recommend using 'capability' as indicated in the title). If the researchers did not intend to use the words as synonyms, it would be helpful to include a definition or description of the difference between the terms in the introduction section.**

Response: I thank the reviewers for their careful suggestions. I have replaced “physical capacity” with “physical capability” throughout the manuscript and highlighted the changes in yellow.

To estimate the extent to which physical capability, depressive symptoms, balanced

better physical capability is an important predictor of stroke survivors’ QoL [8]. Additionally, physical capability is associated with balance function [13], which may

2)***In the abstract, under the results heading there is a ' before physical capability (last sentence of the results section). Please either remove the ' or complete the statement by adding an ' after depressive symptoms.**

Response:
I would like to thank the reviewers for their careful reading. I have revised and highlighted the text in yellow. This corresponds to lines 44 to 46 of the manuscript.

0.322, *p* = 0.002). Hypertension was found a significant moderator of all the direct paths from physical capability to QoL through depressive symptoms (B = 0.412, *p* = 0.015; B = 0.831, *p* = 0.020, respectively). This study provides a better insight into the

3)***In the introduction section, the first sentence is a bit difficult to read because of the punctuation used, my suggestion would be to change the sentence to: Stroke is a primary cause of long-term neurological disability, with >80 million stroke survivors globally in 2016. (so remove the , and change worldwide to globally to enhance the flow of the sentence).**

Response: Thank you to the reviewers for their honest advice. I have revised and highlighted the text in yellow. This corresponds to lines 50 to 51 of the manuscript.

Stroke is a primary cause of long-term neurological disability, with more than 80 million stroke survivors globally in 2016 [1]. Compared to Europe, America, and

4)***In the second paragraph in the introduction (balance), the second sentence is a bit unclear (i.e., is the sedentary lifestyle a result of the stroke or as a result of the fear of falling)? Please rephrase for clarity.**

Response: I would like to thank the reviewers for their careful reading. I have revised

and highlighted the text in yellow. This corresponds to lines 71 to 73 of the manuscript.

related distress behaviors such as fear of falling [16, 17]. The fear of falling stroke survivors frequently leads to activity and participation limitations as a consequence of poststroke physical disability [17]. Therefore, enhancing the balance confidence may

5）***In the balance paragraph, in the sentence after the ABC questionnaire, please change it to it is (i.e., it is also called)**

Response: I would like to thank the reviewers for their careful reading. I have revised and highlighted the text in yellow. This corresponds to lines 75 to 76 of the manuscript.

specific Balance Confidence Scale (ABC)" questionnaire [19]. It is also called Balance self-efficacy (BSE), which means an individual's belief in his or her ability to

6)***Please revise the sentence in the balance paragraph (after reference nr 17) for ease of reading, my suggestion would be: Studies in the last few decades have produced fairly convergent results, indicating that higher balance self-efficacy is linked to better QOL of stroke patients.**

Response: I would like to thank the reviewers for their careful reading. I have revised and highlighted the text in yellow. This corresponds to lines 77 to 79 of the manuscript.

accomplish a task [20]. Studies in the last few decades have produced fairly convergent results, indicating that higher balance self-efficacy is linked to better QoL of stroke patients [21-23]. French et al. proposed a theoretical model explain the mediating role

7)***In the sentence after reference 21, please remove the word 'here'.**

Response: I would like to thank the reviewers for their careful reading. I have revised and highlighted the text in yellow. This corresponds to lines 81 to 82 of the manuscript.

[20]. Using the above theoretical model, we propose the first hypothesis, considering the mediation effect of BSE on the relationship between physical capability and QoL.

8)***In the first sentence on page 11 (after reference nr 24), please change to 'One research study'. In the following sentence, please change contributes to contribute.**

Response: I would like to thank the reviewers for their careful reading. I have revised and highlighted the text in yellow. This corresponds to line 87 of the manuscript.

physical impairments contribute to post-stroke depressive disorders [28]. Thus, we

9)***In the introductory paragraph, regarding hypertension, please elaborate a bit more in terms of what you mean by 'significantly affecting daily lives' - are these activities of daily living?**

Response: We thank the reviewers for their careful suggestions, and we have revised them here. This corresponds to lines 92 to 93 of the manuscript.

QoL [29, 30]. Hypertension is a leading risk factor for stroke in Asia [3, 7], significantly increasing the risk of ADL disability [31]. Hypertension with other comorbid conditions

10)***At the conclusion of the introduction section, the information is a bit confusing (although I do see that you merged hypothesis one and two and reported it as one) - but this can be very confusing. I would recommend that you either adhere to the two hypotheses (as indicated at the end of the introduction section throughout) or that you write out all three (without merging the described hypotheses 1 and 2). Whichever you decide, please remember to adapt the abstract accordingly.**

Response: We thank the reviewers for their careful suggestions, and we have revised them here. We have added hypothesis 3. This corresponds to lines 109 to 112 of the manuscript.

Fig 1. The model tests specific hypotheses: (1) physical capability has an indirect effect on QoL mediated by BSE; (2) physical capability has an indirect effect on QoL by depressive symptoms; (3) stroke risk factors (hypertension/diabetes/gender) moderate the above relationship.

11)***In Figure 1, it would be nice to add either color or a bit more information, so that it is clear which factors are hypothesized to be mediators and which factors are moderators (to align more clearly with the written information).**

Response: We thank the reviewers for their careful suggestions and we have revised Figure 1. This corresponds to lines 113 to 118 of the manuscript.

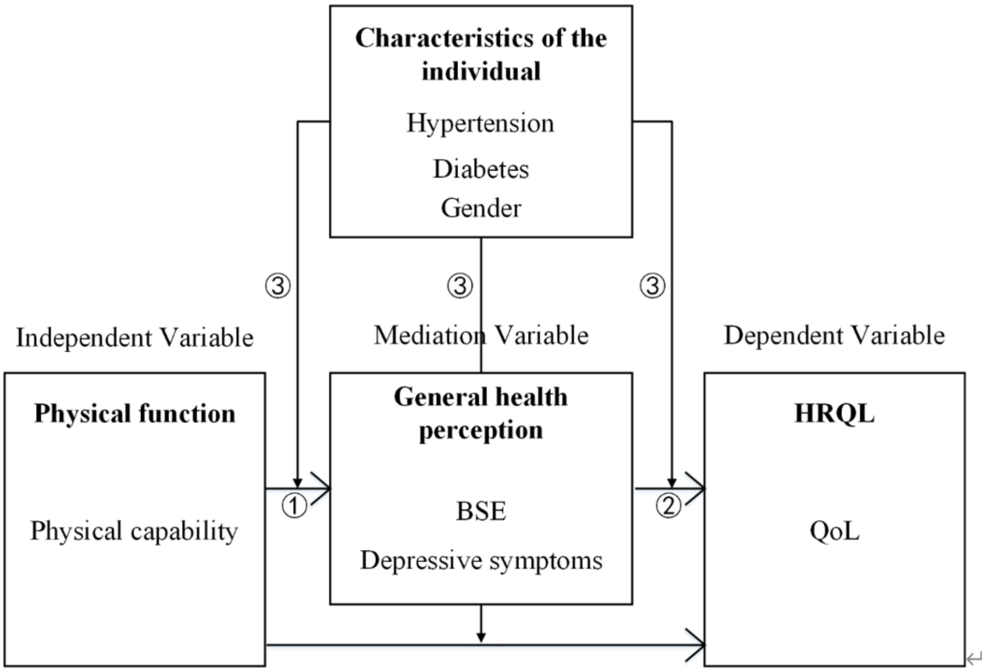

Fig 1. The theoretical model is based on Wilson and Cleary's model. ①: Hypothesis 1 of the current study; ②: Hypothesis 2 of the current study; ③: Hypothesis 3 of the current study. BSE, Balance Self-Efficacy; HRQL, Health-Related Quality of Life; QoL, Quality of Life. The arrows represent the hypothesized directional relationships between the variables in the model.

12)***Regarding the exclusion criteria, please be a bit more specific regarding 'several mental disorders' - are these disorders classified on the DSM, and how many were considered to be too many (for example, if a patient had anxiety and depression were they excluded, or were more than 2 diagnoses required for exclusion?). Were previous disability excluded as well? For example, if the patient had an amputation / severe orthopedic condition? (as these factors may influence the results of the study)**

Response: I thank the reviewers for their honest advice. I have revised the exclusion criteria and highlighted the text in yellow. We refined the inclusion-exclusion criteria. This corresponds to lines 130 to 136 of the manuscript.

informed consent. The participant exclusion criteria were as follows: (I) presence of any mental diagnosis before stroke according to the Diagnostic and Statistical Manual of Mental Disorders classification (i.e., major depressive disorder, bi-directional affective disorder, and obsessive-compulsive disorder, etc.); (II) malignant tumors, and/or; (III) dementia, loss of consciousness, or severe cardiopulmonary, liver, or kidney dysfunctions; (IV) pre-existing physical disability from any cause, such as fracture, osteoarthritis, etc. Out of the 1,765 participants, 245 did not meet the inclusion

13)***Figure 2 does not indicate any additional information from what was provided in the text and the researchers can consider omitting it from the manuscript.**

Response: Thank you to the reviewers for their suggestions. Since we have described the participant inclusion process in the Participants section of the methodology, the original Figure 2 has been removed.

14)***In the Procedure section (under the Methods heading) I referred to the referenced paper regarding quality controls, but it is not clear what the researchers are referring to - i.e., what quality controls were used? (This information is from the referenced study: Subsequently, 10 hospitals were invited to participate in relevant training programs every 3 months, and two trained investigators made irregular visits to different hospitals to collect information and conduct quality control. - is this what the researchers are referring to? Because it is not a lot of information, it might be useful to write it out in the current study (under review).**

Response: I would like to thank the reviewers for their careful reading. We describe the data collection quality control process in detail. I have revised and highlighted the text in yellow. This corresponds to lines 140 to 147 of the manuscript.

The case data used in this cross-sectional study are stroke patients from ten different rehabilitation facilities in Yunnan Province, China. First, ten hospitals were invited to participate in the training program every three months. Subsequently, two trained researchers visit different hospitals from time to time to collect information and perform quality control [13]. After patients sign an informed consent form, data collectors perform one standardized assessment, including a neurological assessment (demographic data, clinic data, neurological and neuropsychological functional assessments). Assessment outcomes and patient information were entered into the

15)***In the results section, please revise the second sentence, I suspect it is supposed to read: The majority of participants had an ischemic stroke, while hemorrhagic- and**

other strokes were reported in 101 and 13 patients respectively.

Response: I would like to thank the reviewers for their careful reading. I have revised and highlighted the text in yellow. This corresponds to lines 205 to 208 of the manuscript.

years) were involved in this study. The majority of participants (556, 83.0%) had an ischemic stroke, while hemorrhagic- and other strokes were reported in 101 (15.1%) and 13 (1.9%) patients respectively. Of the total number, 292 (43.6%) had a left-sided stroke (right hemiparesis), and 297 (44.3%) had a right-sided stroke (left hemiparesis).

16)***Please clarify whether left stroke and right stroke refer to left/right hemiplegia/paresis or left or right cerebral involvement.**

Response: Thank you to the reviewers for their suggestions. Left/right hemiparesis in the manuscript implies left/right paralysis or right/left stroke. This corresponds to lines 207 to 208 of the manuscript.

and 13 (1.9%) patients respectively. Of the total number, 292 (43.6%) had a left-sided stroke (right hemiparesis), and 297 (44.3%) had a right-sided stroke (left hemiparesis).

17)***Just for noting - I found the path analysis very interesting and Figure 3 assisted with interpreting and understanding all the information (which can sometimes be a bit overwhelming)**

Response: Thank you to the reviewers for their suggestions. We have revised this figure and added a note to explain it. This corresponds to lines 247 to 253 of the manuscript.

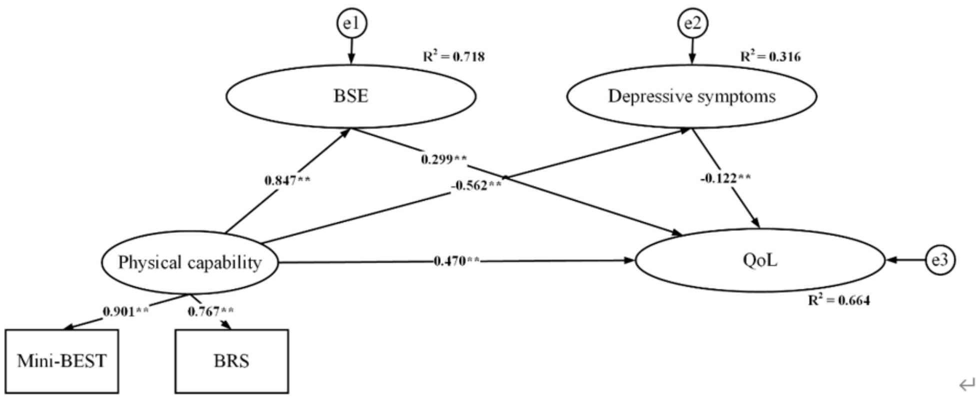

Fig 2. Model fit (χ^2 = 14.233, χ^2/df = 4.744, *p* = 0.002. RMSEA = 0.075, SRMR = 0.010, TLI = 0.981, CFI = 0.994). Mini-BEST, mini-Balance Evaluation Systems Test; BRS, Brunnstrom; BSE, Balance self-efficacy; QoL, Quality of Life. This model is consistent with hypotheses 1 and 2 of the theoretical model. BSE and Depressive symptoms are mediating variables between Physical capability and QoL.

18)***I think your interpretation of the diabetes/gender effect may be a bit harsh, i.e., that your hypothesis was rejected. I would recommend that you add that although the results from your study didn't support your hypothesis, the smaller (significantly so?) number of females (compared to males) and the small number of patients with diabetes may have influenced the results.**

Response: We thank the reviewers for their suggestions and we add a note of clarification. This corresponds to lines 326 to 329 of the manuscript.

to QoL via BSE' pathway. Although our findings did not show that diabetes and gender moderated the relationship between physical capability and QoL, this may be attributable to the relatively small number of individuals with diabetes and females in this cohort. Therefore, further research is needed with a larger and more diverse sample.

19)***In the discussion section, in the first sentence of the second paragraph (however, we found no...) please elaborate the sentence a bit more so that the 'relationship' you are referring to is clear.**

Response: Thanks to the reviewer's suggestion, we have revised this sentence. This corresponds to lines 299 to 300 of the manuscript.

However, we found no mediation effect of depressive symptoms on the relationship between physical capability and QoL. According to French et al. [19], depressive

20)***Discussion section: When using in-text references, you don't have to include the initials (so it will become Sok et al.)**

Response: I thank the reviewers for their honest advice. I have revised and highlighted the text in yellow. This corresponds to lines 311 to 312 of the manuscript.

symptoms. This result is consistent with Sok et al. showing a strong association between depressive symptoms and hypertension [35]. Accordingly, another study reported that

21)***Please reference the sentence on page 17, i.e., 'it could be that antidepressant therapy interferes with blood pressure control in hypertension patients' - alternatively, if this is not a proven fact, indicate it (for example, the possibility exists that antidepressant therapy interferes with blood pressure control in hypertensive patients, but further research on the topic is warranted).**

Response: We thank the reviewers for their suggestions and we apologize for the lack of rigor in the language of the manuscript. We have revised the language of the manuscript. This corresponds to lines 313 to 315 of the manuscript.

depressed patients with hypertension had poor control of blood pressure [53]. The possibility exists that antidepressant therapy interferes with blood pressure control in hypertension patients, but further research on this topic is warranted. Additionally, a

22)***The sentence directly after the one referred to in comment 21 needs revision - so that the link between postmenopausal women and the study becomes clear (for example: Additionally, a recent study showed that increased walking (physical) activity lowers hypertension risk in postmenopausal women [52]. Our study also indicated that physical activity positively influences hypertension risk, even in a mixed-gender population.**

Response: Thanks to the reviewer's suggestion, we have revised this sentence. This corresponds to lines 317 to 318 of the manuscript.

in postmenopausal women [54]. Our study also indicated that physical activity positively influences hypertension risk, even in a mixed-gender population. Conversely,

23)***The final sentence of the discussion needs to be preceded by a sentence regarding balance so that the linkage becomes clear.**

Response: Thanks to the careful suggestions of the reviewers, we have added a sentence on balanced functioning and QoL before the last sentence of the discussion to make the whole more logical and naturally articulated. This corresponds to lines 336 to 337 of the manuscript.

rehabilitation. Patients show improved balance function, which naturally enhances balance confidence, leading to a significant improvement in QoL [18]. Health

24)***Throughout the discussion section OR (alternatively) in the limitations section, the researchers should make some recommendations for future research (either to say how limitations can be minimized in future research or recommendations for future research based on the findings of the study.**

Response: We thank the reviewers for their suggestions and we have revised the limitations section. This corresponds to lines 341 to 351 of the manuscript.

There are some limitations to our research. First, we did not consider the complex nature of the QoL index, which may complicate relationships among the various missing data of clinical variables. Researchers should implement rigorous strategies to minimize missing data and place greater emphasis on exploring the intricacies of the QoL index in more detail, taking into account its multiple dimensions. Second, we did not take into account the impact of stroke subtypes, a possible confounding parameter in this study. Future research should consider stratifying stroke patients by subtype, which would facilitate a more precise analysis of how different stroke types may influence the outcomes. Third, the cross-sectional study design restricts the interpretation and generalization of our results. An additional longitudinal study may help verify the conclusions of this study.↵

25)***Please ensure the correct tense is used in the conclusion paragraph (the study**

has been conducted, so the section should be in the past tense).

Response: I thank the reviewers for their careful reading. I have modified the tense of the conclusion section to past tense. This corresponds to lines 353 to 358 of the manuscript.

This study provided a better insight into the relationship between physical capability and QoL via BSE in stroke patients, which may help establish appropriate treatment for these individuals. Our results uncovered the underlying moderating effect of hypertension on relationships between physical capability, depressive symptoms, and QoL. An effective rehabilitation strategy can achieve concrete targets for stroke risk patients via BSE, improving their QoL [57].
