# Supplementary material for: Mediation and moderation analysis of the association between physical capability and quality of life among stroke patients
Source: PLoS One. 2025 Jun 10;20(6):e0320833. doi: 10.1371/journal.pone.0320833 (PMC12151468; doi:10.1371/journal.pone.0320833)
Supplement: Attachment — Submitted filename: COMMENTS.docx [file pone.0320833.s006.docx]

**COMMENTS**

The manuscript has really improved and corrections have been well done. There are only two suggestions to be made.

1. There is the use of ‘stroke patients’ and ‘stroke survivors’ interchangeably. I think you would rather use ‘stroke survivors’. Kindly change all the stroke patients in the writing to survivors.
2. In the results section, there was a reference to fig1 but I couldn’t find fig1 in the results. Also, ensure that all the values in table 4 are presented such that the numbers (eg. 0.000) are in line instead of some coming under others. Therefore increase the width of the cells.
3. There are also a few corrections in track changes.
