# Supplementary material for: Mediation and moderation analysis of the association between physical capability and quality of life among stroke patients
Source: PLoS One. 2025 Jun 10;20(6):e0320833. doi: 10.1371/journal.pone.0320833 (PMC12151468; doi:10.1371/journal.pone.0320833)
Supplement: Attachment — Submitted filename: Response_to_Reviewers_auresp_2.docx [file pone.0320833.s008.docx]

Dear editors and reviewers:

Re: Manuscript ID: PONE-D-24-45706R1 and Title: Mediation and moderation analysis of the association between physical capability and quality of life among stroke patients

Thank you for your letter and the reviewers’ comments concerning our manuscript entitled “PONE-D-24-45706R1” (ID). Those comments are valuable and very helpful. We have read through the comments carefully and have made corrections. Based on the instructions provided in your letter, we uploaded the file of the revised manuscript. Revisions in the main text are highlighted in yellow as additions or modifications. The responses to the reviewer's comments are marked in yellow and presented following.

We would love to thank you for allowing us to resubmit a revised copy of the manuscript and we highly appreciate your time and consideration.

Sincerely.

Liqing-Yao

Reviewer #1:

1)* **There is the use of ‘stroke patients’ and ‘****stroke survivors’ interchangeably. I think you would rather use ‘stroke survivors’. Kindly change all the stroke patients in the writing to survivors.**

Response: I thank the reviewers for their honest advice. We have changed “stroke patients” to “stroke survivors” throughout the manuscript. I have made changes to the text and highlighted them in yellow, some of which are shown below.

2)* **In the results section, there was a reference to fig1 but I couldn’t find fig1 in the results. Also, ensure that all the values in table 4 are presented such that the numbers (eg. 0.000) are in line instead of some coming under others. Therefore increase the width of the cells.**

Response: Thank you to the reviewers for their constructive comments. I have changed the description in the results section. And I have revised Table 4 to make the table presentation more straightforward and aesthetically pleasing.

3)* **There are also a few corrections in track changes.**

Response: I thank the reviewers for their careful suggestions. We have revised all the marked places throughout the manuscript, some of which are shown below.
